# Computational models of compound nerve action potentials: Efficient filter-based methods to quantify effects of tissue conductivities, conduction distance, and nerve fiber parameters

**DOI:** 10.1371/journal.pcbi.1011833

**Published:** 2024-03-01

**Authors:** Edgar Peña, Nicole A. Pelot, Warren M. Grill

**Affiliations:** 1 Department of Biomedical Engineering, Duke University, Durham, North Carolina, United States of America; 2 Department of Electrical and Computer Engineering, Duke University, Durham, North Carolina, United States of America; 3 Department of Neurobiology, Duke University School of Medicine, Durham, North Carolina, United States of America; 4 Department of Neurosurgery, Duke University School of Medicine, Durham, North Carolina, United States of America; The University of Iowa College of Engineering, UNITED STATES

## Abstract

**Background:**

Peripheral nerve recordings can enhance the efficacy of neurostimulation therapies by providing a feedback signal to adjust stimulation settings for greater efficacy or reduced side effects. Computational models can accelerate the development of interfaces with high signal-to-noise ratio and selective recording. However, validation and tuning of model outputs against in vivo recordings remains computationally prohibitive due to the large number of fibers in a nerve.

**Methods:**

We designed and implemented highly efficient modeling methods for simulating electrically evoked compound nerve action potential (CNAP) signals. The method simulated a subset of fiber diameters present in the nerve using NEURON, interpolated action potential templates across fiber diameters, and filtered the templates with a weighting function derived from fiber-specific conduction velocity and electromagnetic reciprocity outputs of a volume conductor model. We applied the methods to simulate CNAPs from rat cervical vagus nerve.

**Results:**

Brute force simulation of a rat vagal CNAP with all 1,759 myelinated and 13,283 unmyelinated fibers in NEURON required 286 and 15,860 CPU hours, respectively, while filtering interpolated templates required 30 and 38 seconds on a desktop computer while maintaining accuracy. Modeled CNAP amplitude could vary by over two orders of magnitude depending on tissue conductivities and cuff opening within experimentally relevant ranges. Conduction distance and fiber diameter distribution also strongly influenced the modeled CNAP amplitude, shape, and latency. Modeled and in vivo signals had comparable shape, amplitude, and latency for myelinated fibers but not for unmyelinated fibers.

**Conclusions:**

Highly efficient methods of modeling neural recordings quantified the large impact that tissue properties, conduction distance, and nerve fiber parameters have on CNAPs. These methods expand the computational accessibility of neural recording models, enable efficient model tuning for validation, and facilitate the design of novel recording interfaces for neurostimulation feedback and understanding physiological systems.

## Author summary

Nerves transmit signals that carry information about organ function, and this information could help improve our understanding and treatment of diseases like high blood pressure and diabetes. Unfortunately, the signals are noisy, and developing ways to avoid or cancel out this noise is slow because physically building and testing each possible solution is time-consuming and resource-intensive. We developed a software tool to simulate recorded neural signals very efficiently from realistic nerve models using the computational power of a standard desktop. Our new tool showed that nerve signals are very sensitive to the details of what is being recorded and how it is being recorded, suggesting ways to enhance signal quality and thus reduce the impact of noise. The software design will make models of nerve recordings accessible to individuals with low computational resources. Our tool will accelerate the ability of researchers to develop new methods of extracting information from peripheral nerves, including in preclinical and clinical studies of different disease targets.

## 3 Introduction

Nerves transmit information about organ function that can enhance our understanding of physiological systems and serve as feedback for closed-loop electrical stimulation devices to treat hypertension [1,2], diabetes [3,4], inflammatory disorders [5], and other diseases [6,7]. Given the prevalence of recording artifacts [8] and limited recording selectivity and longevity of conventional recording methods [9], a range of new electrode interfaces [10–12] and signal analysis methods [13–15] have emerged to extract information with reduced noise, greater selectivity, and improved stability over time. Computational modeling has great potential to accelerate the design of effective interfaces by allowing high throughput parameter evaluation before manufacturing electrodes: modeling can enable quantitative prediction of the effects of design parameters and other interface characteristics on the recorded electric potentials from a nerve target. We designed and implemented a highly efficient computational modeling method to simulate recorded neural signals from large populations of fibers, and we applied this pipeline to conduct a comprehensive sensitivity analysis of the effects of model parameters on the simulated nerve response.

Electrically evoked compound nerve action potentials (CNAPs) are recorded clinically in nerve conduction studies to diagnose nerve health [16,17] and are ubiquitous outcome measures in electrophysiological studies. Studies since the pioneering work of Gasser, Erlanger, and Grundfest [18,19] modeled CNAPs as summations of single fiber action potentials (SFAPs) propagating at a distinct conduction velocity (CV) for each nerve fiber. Early models used measured or assumed SFAP time courses (Table 1). More sophisticated models calculated SFAP time courses using measured or assumed transmembrane potentials and a cylindrical, axially symmetric volume conductor model. Most models since [20] used numerical rather than analytical methods to extract action potential transmembrane currents from biophysical models of nerve fibers and used these currents as point sources within a numerically-solved volume conductor model.

**Table 1:** Summary of different computational methods used to construct recordings of SFAPs (single fiber action potentials) and CNAPs (compound nerve action potentials) since the seminal work of Stegeman and colleagues [21], excluding studies that solely aimed to reconstruct fiber diameter distributions. Studies from the same authors that used the same type of model are grouped. Vm(t): time course of transmembrane potential; Im(t): time course of transmembrane current.

Source	SFAP	Source of or equation for Vm(t) or Im(t)	Volume conductor model
Olson and BeMent, 1981 [22]	triangular (monophasic)	N/A	N/A
Kincaid et al., 1988 [23]	triangular (monophasic, biphasic, and triphasic)	N/A	N/A
Okajima et al., 1994 [24]	triangular (triphasic)	N/A	N/A
Wijesinghe et al., 1991 [25] Wijesinghe and Wikswo, 1991 [26]	from Vm(t) and volume conductor model	Empirical waveform from literature	analytical
Stegeman and De Weerd, 1982a [27] Schoonhoven et al., 1986a [28] Schoonhoven et al., 1986b [29] Stegeman and De Weerd, 1982b [30]	from Vm(t) and volume conductor model	Empirical waveform from literature	analytical
Donohoe et al., 2019 [31]	from Vm(t) and volume conductor model	Vint = (36,864 mV)*(t^3)*exp(-8*t)-70 mV	analytical
Struijk, 1997 [20] Andreasen et al., 2000 [32] Andreasen and Struijk, 2002 [33]	from Im(t) and volume conductor model	HH-style model	numerical (rotationally symmetric)
Sabetian et al., 2016 [34] Sabetian et al., 2017b [35] Sabetian et al., 2017a [36] Sabetian and Yoo, 2019 [37] Sabetian and Yoo, 2020 [38]	from Im(t) and volume conductor model	HH-style model	numerical
Lubba et al., 2019 [39]	from Im(t) and volume conductor model	HH-style model	numerical (rotationally symmetric)
Tarotin et al., 2019 [40]	from coupled volume conductor model	HH-style model coupled with volume conductor model	numerical
Eiber et al., 2021 [41]	from Im(t) and volume conductor model	HH-style model	numerical

**Fig 1:**
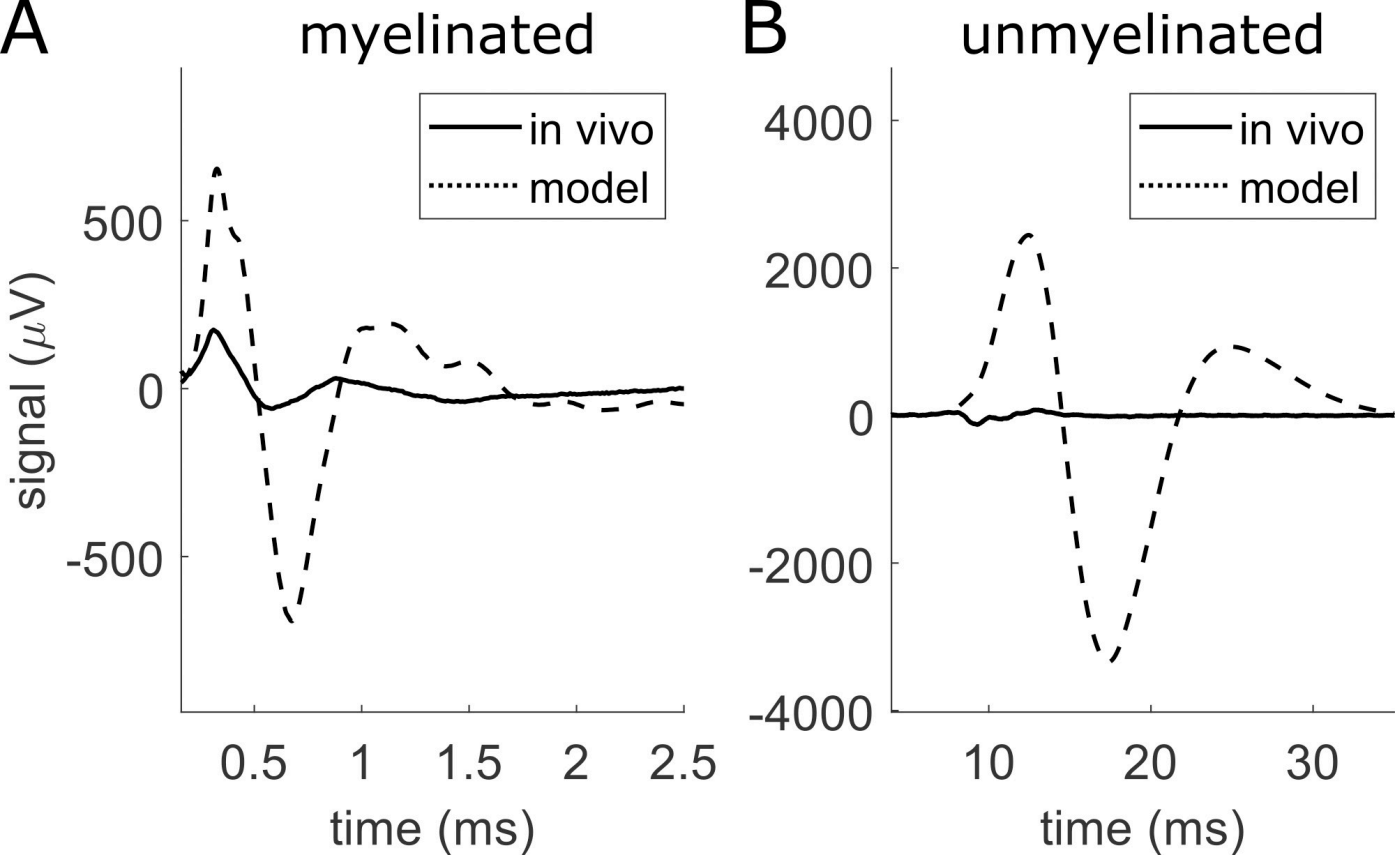
Comparison of rat cervical vagus nerve compound nerve action potentials (CNAPs) that we recorded in vivo (solid) and that we modeled computationally (dashed) from myelinated (A) and unmyelinated (B) fibers with a conduction distance of 11 mm. We simulated the model using values from literature, including 0.16 S/m for the surrounding medium, and using a fully sealed cuff.

Despite the power of numerical approaches and the range of studies that used those approaches to quantify the effects of tissue and electrical recording parameters on SFAP or CNAP amplitude, shape, or latency (Table 2), there are limited data validating modeled neural recordings against in vivo signals. Such model validation—and appropriate model tuning to match model outputs to in vivo data—are essential for neural interface design since model parameters based on literature values alone can produce outputs with large discrepancies in amplitude and latency relative to in vivo data (Fig 1). Unfortunately, model validation, model tuning, and application of models for design all require the generation of many modeled signals, which remains computationally infeasible with present methods. Each nerve may contain tens of thousands to hundreds of thousands of nerve fibers, and the transmembrane current time series for each compartment of each fiber must be simulated and stored. Several studies aimed to reduce this computational burden using methods such as defining action potential templates repeated over time to reconstruct transmembrane current matrices [20, 35, 42, 43], simulating a subset of representative fibers [41], or using filter-based methods to avoid transmembrane current matrices entirely [44]. However, such methods have not been compiled and validated for accuracy, and computing CNAPs for tens of thousands of fibers or more remains burdensome for most research and clinical environments. Here, we developed a highly efficient method that leverages and extends the range of previous computational techniques, we validated the method against a brute force approach, and we applied the method to quantify the CNAP sensitivity to a range of model parameters and to compare modeled CNAPs with in vivo CNAP recordings. Our study reveals key parameters relevant to modeling CNAPs and contributes important data for validating future models. The modeling framework presented here is publicly available.

**Table 2:** Summary of published computational sensitivity analyses of stimulation-evoked SFAPs and CNAPs. Where applicable, the summary indicates the correlation with SFAP or CNAP amplitude (i.e., ‘+’ if increasing the parameter increased the SFAP or CNAP amplitude; ‘-’ if the opposite occurred). σ_r_ is radial conductivity. σ_z_ is longitudinal conductivity.

Parameter	Correlation with Amplitude of SFAP or CNAP	Source	Finding
*Tissue Properties*			
σ_r_	+	Stegeman et al., 1979 [21]	decreasing the radial conductivity of the endoneurium decreased SFAP amplitude
σ_r_, σ_z_	+	Wijesinghe et al., 1991 [25]	increasing either endoneurium’s radial conductivity (6x) or the longitudinal conductivity (2x) increased the peak-to-peak CNAP amplitude (~1.5x or ~2.5x)
intracellular conductivity	+	Wijesinghe and Wikswo, 1991 [26]	“very sensitive”; increasing the intracellular conductivity from 0.1 to 2.5 S/m increased SFAP amplitude from ~0.02 to ~0.2 μV; also, increasing intracellular conductivity by 50% increased SFAP conduction velocity by 25%
extracellular conductivity	-	Wijesinghe and Wikswo, 1991 [26]	increasing the extracellular conductivity decreased SFAP amplitude
endoneurial anisotropy	-	Wijesinghe et al., 1991 [25]	increasing endoneurial anisotropy decreased SFAP amplitude
		Wijesinghe et al., 1991 [25]	frequency-dependent endoneurium conductivity produced CNAPs with similar or lower amplitudes than fixed endoneurium conductivity
perineurium thickness	-	Eiber et al., 2021 [41]	thicker perineurium reduced SFAP amplitude
*Temperature*			
	+	Wijesinghe and Wikswo, 1991 [26]	reducing the temperature from 30 to 25°C (and from 25 to 20°C) reduced CNAP peak-to-peak amplitude from 80 to 40 μV (and 40 to 20 μV)
		Stegeman and De Weerd, 1982a [27]	CNAP duration increased with temperature; conduction velocity increased linearly with temperature
*Cuff Geometry and Distances*			
cuff opening size	-	Struijk, 1997 [20]	63 μm cuff opening (~2% of the circumference of the inner cuff surface) reduced SFAP amplitude by 50% compared to no gap; the gap was modeled by reducing the conductivity of the entire axially symmetric cuff to ~1% of the surrounding medium)
	-	Struijk, 1997 [20]	SFAP peak-to-peak amplitude was an order of magnitude smaller in a homogeneous model (100% cuff opening) compared to a model with a fully sealed cuff
cuff insulation length	+	Struijk, 1997 [20]	SFAP peak-to-peak amplitude increased substantially with cuff length up to ~30 mm for 10 μm fibers (with monopolar electrode centered in the cuff)
	+	Andreasen and Struijk, 2002 [33]	for 10–20 μm fibers, shortening cuff length from 50 mm to 20 mm reduced SFAP peak-to-peak amplitude by up to 20% and reduced RMS of electroneurogram by up to 50%
	+	Lubba et al., 2019 [39]	SFAPs from myelinated fibers had larger peak-to-peak amplitudes at cuff lengths up to 1.3 mm (for 2 μm fibers) or up to 16 mm (for ~3.8 μm fibers), while the SFAPs from unmyelinated fibers had maximal peak-to-peak amplitude at a cuff length of ~1.1 mm
distance between edge and electrode	+	Struijk, 1997 [20]	SFAP negative peak amplitude became more negative as the electrode contact was placed farther away from the cuff edge (up to a plateau at ~15 mm)
		Struijk, 1997 [20]	phases of SFAP changed with electrode location
electrode-to-axons distance (R)	-	Schoonhoven et al., 1986a [28]	recordings at the surface of the skin were ~3-5x smaller and a bit broader than “near-nerve” recordings
	-	Schoonhoven et al., 1986b [29]	recordings at the surface of the skin were ~5x smaller and a bit broader than “near-nerve” recordings
	-	Wijesinghe et al., 1991 [25]	CNAP peak-to-peak decreased substantially from R = 2 mm to R = 24 mm
	-	Wijesinghe and Wikswo, 1991 [26]	increasing electrode-to-axon distance by 25% (0.8 to 1 mm) or by 50% (0.8 to 1.2 mm) decreased CNAP amplitude by 18% or by 22% (respectively)
	-	Struijk, 1997 [20]	in a homogeneous model, SFAP peak-to-peak amplitude decreased at 1/R for small R values and at 1/R^3^ for large R values
conduction distance	-	Olson and BeMent, 1981 [22]	increasing conduction distance by ~15% decreased the CNAP amplitude by ~15%
	-	Stegeman and De Weerd, 1982b [30]	conduction distances 6, 9, 12, 15, 18, 21, 24 cm produced CNAP amplitudes of 28, 20, 16, 12, 10, 8, 7 μV
	-	Schoonhoven et al., 1986b [29]	increasing conduction distance from 6 to 15 cm (2.5x) decreased CNAP amplitude by ~4x and made CNAP shape more jagged
	-	Wijesinghe et al., 1991 [25]	increasing conduction distance by ~50% decreased the CNAP amplitude by ~50%
	-	Wijesinghe and Wikswo, 1991 [26]	increasing conduction distance from 110 to 80 mm (and 80 to 50 mm) decreased CNAP peak-to-peak amplitude by 50 to 25 μV (and 25 to 18 μV)
	-	Tarotin et al., 2019 [40]	for conduction distances 10, 19, 25, 35 cm (unmyelinated fibers based on [45]) and 0.4, 0.8, 1, 1.4 cm (unmyelinated fibers based on [46]), negative peak magnitude of CNAP was ~7, 6, 4, 2.5 mV [45] and 1, 0.6, 0.5, 0.3 mV (C fibers)
*Fiber diameter sizes*, *distributions*, *and fiber geometry*			
mean fiber diameter	+	Olson and BeMent, 1981 [22]	~20% and ~25% decrease in mean fiber diameter of Gaussian distributions decreased the CNAP amplitude by ~50%
	+	Okajima et al., 1994 [24]	increasing the mean and decreasing the standard deviation of fiber diameter distributions both increased CNAP amplitude due to decreased temporal dispersion
	+	Andreasen and Struijk, 2002 [33]	"RMS of the electroneurogram depends linearly on the fiber diameter in contrast to the peak-to-peak amplitude of the SFAP, which increases nearly with the square of the diameter"
	+	Donohoe et al., 2019 [31]	axon population with large fiber diameters (4,000 fibers at 9.5 μm) produced a CNAP with 8x larger amplitude than an axon population with small fiber diameters (6,000 fibers at 4.5 μm)
number of fibers	+	Schoonhoven et al., 1986a [28]	adding 3,000 fibers with CV>30 m/s caused CNAP amplitude to increase ~10x compared to just 5,000 fibers with mostly CV<30 m/s
	+	Stegeman et al., 1979 [21]	increasing the number of fibers increased CNAP amplitude and markedly altered CNAP amplitude at low fiber counts
	+	Schoonhoven et al., 1986b [29]	CNAP from a “pathological nerve” (i.e., 3,500 large fibers) had ~10x weaker signal than CNAP from a “normal nerve” (i.e., 8,000 large fibers)
			Randomly sampling fiber diameters from a fixed distribution produced CNAPs with different shape (but not amplitude) across samples of fibers
		Eiber et al., 2021 [41]	a population of axons with a range of distributed fiber diameters produced CNAP that differed in shape and amplitude from CNAP produced by a population of axons with a single homogeneous fiber diameter
fiber trajectory		Lubba et al., 2019 [39]	little effect of myelinated fiber tortuosity, but large effect on SFAP latency, amplitude, and shape of unmyelinated fiber tortuosity

## 4 Methods

### 4.1 Ethics statement

All procedures were approved by the Institute for Animal Care and Use Committee of Duke University (Durham, NC) and were in accordance with the Guide for Care and Use of Laboratory Animals (8th edition).

### 4.2 Overview

**Fig 2:**
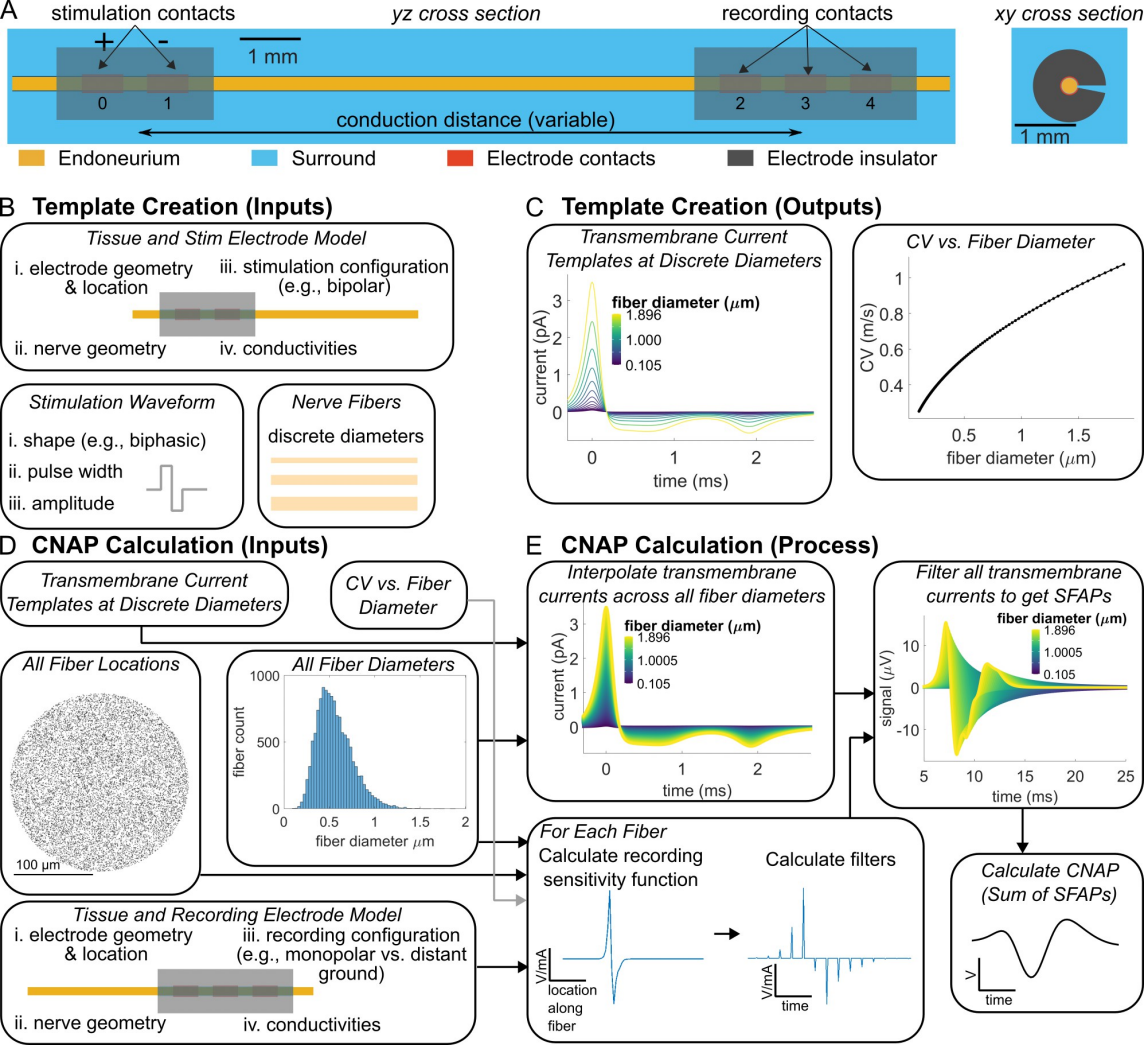
Overview of modeling CNAP recordings from rat cervical vagus nerve. (A) A bipolar stimulation electrode activated the nerve fibers at the proximal end of the nerve. A tripolar electrode recorded the CNAP at each contact—in a monopolar configuration—at the distal end of the nerve. The volume conductor model represented the monofascicular nerve as a cylinder with a perineurium (not illustrated) and an anisotropic endoneurium, and it represented the electrodes as electrode contacts within insulating cuffs. The recording electrode had a cuff opening of either 0° or 16°. A conductive material (“surround”) filled the space within and around the nerve and cuffs. (B) Template creation inputs included the stimulation volume conductor model, a stimulation waveform, and a set of 193 (myelinated) or 97 (unmyelinated) discrete fiber diameters that defined a population of nerve fibers at the centroid of the nerve to simulate in a biophysical model. (C) Template creation outputs included CV and transmembrane currents for each of the 193 (myelinated) or 97 (unmyelinated) simulated fibers. (D) CNAP calculation inputs included a recording volume conductor model, fiber diameter measurements, fiber locations, and the template creation outputs (i.e., transmembrane current templates and CV vs. fiber diameter relationship). Fiber diameter measurements and random fiber locations defined a population of 1,676 (myelinated; not illustrated) or 13,283 (unmyelinated; illustrated) nerve fibers to be recorded. Fiber diameter measurements were obtained from a publicly available dataset [47] and transformed by the shape-adjusted ellipse method [48]. (E) We interpolated transmembrane current templates across all fiber diameters. We calculated the recording sensitivity functions at all fiber locations via the recording volume conductor model. We calculated a filter for each fiber by inserting zeros into the recording sensitivity function such that the time between non-zero samples equaled the internodal length divided by CV. We generated SFAPs by convolving each filter with an interpolated transmembrane current template, and we superposed SFAPs to generate CNAPs.

We modeled CNAPs from a rat cervical vagus nerve by using biophysical cable models of axons and three-dimensional finite element models of the nerve and cuff electrode, with inhomogeneous tissue and material properties (Fig 2A). We then used those models to develop a method for highly efficient CNAP modeling (Fig 2B–2E). Finally, we used this method to conduct a comprehensive analysis of the effects of key biological and electrode parameters on CNAP recordings.

**Fig 3:**
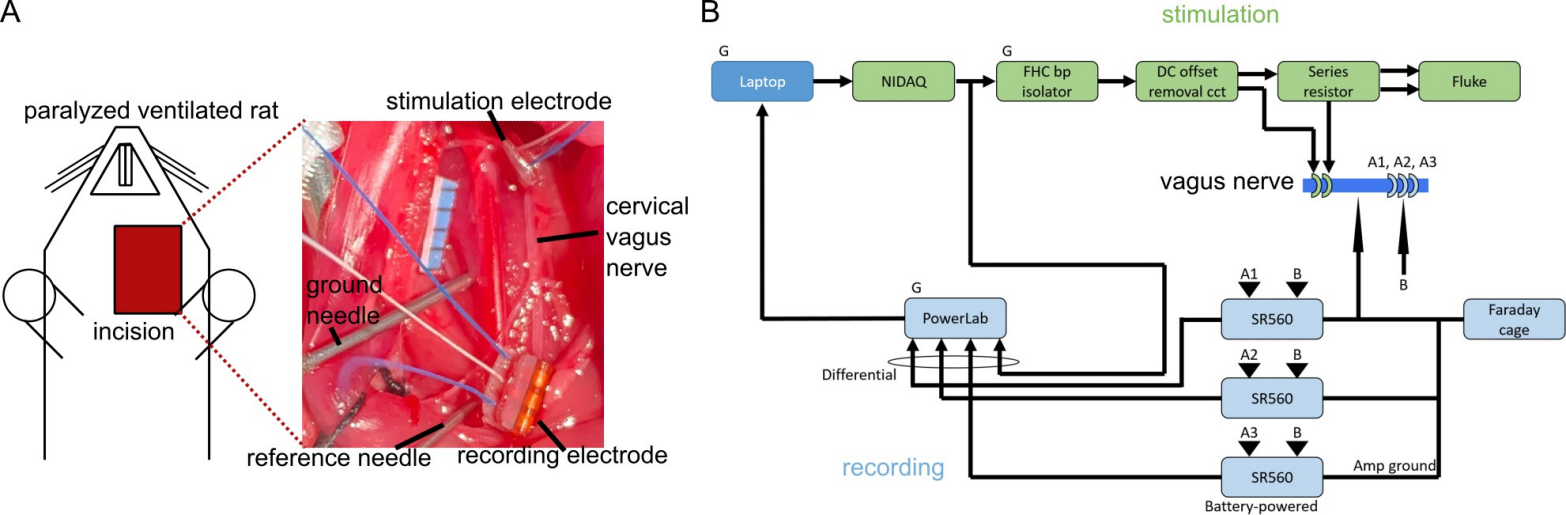
In vivo CNAP recording setup overview. (A) Surgical setup of stimulation and recording electrodes along the rat cervical vagus nerve. The black tick marks on the blue ruler are 1 mm apart. (B) Block diagram of stimulation (green) and recording (light blue) hardware setup. “G” denotes a unit plugged into wall power. “FHC bp isolator” is a current source, “Fluke” is a battery-powered oscilloscope, and “SR560” is a preamplifier. The “ground needle” in panel (A) was connected to the Faraday cage, while the “reference needle” was connected to channel B of all three SR560 units.

Our overall CNAP modeling approach consisted of the following: (1) Construct a volume conductor model of the nerve with a recording cuff electrode and a stimulation cuff electrode (Fig 2A); (2) Stimulate a population of biophysical axon models extracellularly using the spatial potentials from the volume conductor model with current delivered through the stimulation electrode (Fig 2B); (3) Extract transmembrane currents produced by those action potentials from every compartment of every axon (Fig 2C); (4a) In a brute force approach, calculate the potential at the recording cuff by multiplying all transmembrane currents at every compartment and every point in time with the recording sensitivity function, or (4b) in our efficient approach, calculate the potential at the recording cuff by interpolating a small number of action potential templates across fiber diameters and filtering them (Fig 2D and 2E). Our newly developed efficient methods enabled accurate CNAP modeling, evaluation of the quantitative impact of model parameters, and comparison against in vivo data.

### 4.3 Volume conductor model

Table 3 summarizes model parameters used. We used COMSOL Multiphysics v6.1 (Burlington, MA) to construct a finite element volume conductor model of a monofascicular rat cervical vagus nerve instrumented with a bipolar stimulation electrode and a tripolar recording electrode (Fig 2A). We represented the nerve as a cylinder of 30 mm length and 247 μm diameter consistent with the average rat cervical vagus nerve radius reported in [49] assuming no nerve shrinkage. We modeled the endoneurium as an anisotropic medium (0.57 S/m longitudinally, 0.17 S/m radially [50,51]). We modeled the perineurium using a thin layer approximation with COMSOL’s contact impedance boundary condition; the perineurium was 4.56 μm in thickness based on the equation for rat vagus nerve in [49], and its conductivity was 8.4e-4 S/m [51]. The electrodes had silicone insulation tubing (1e-12 S/m [52]) and either two or three platinum contacts (9.43e6 S/m [53]). The nerve was centered radially within the electrodes. The electrodes had an opening of 0° or 16°. To simplify the sensitivity analysis, we set the medium surrounding the cuff and nerve to be isotropic and to have the same conductivity as the fluid filling the 10 μm-thick space between the nerve and the cuff (default value equal to epineurium (0.16 S/m [54]). The stimulation and recording cuff geometry consisted of cylindrical tubes of length 2,580 μm and 3,650 μm, respectively, with an inner diameter of 265 μm, an outer diameter of 1,190 μm, and contacts having the same angular gap as the cuff (0° to 16°), contact length 680 μm, and contact pitch 1,070 μm (center-to-center). In models where conduction distance was not explicitly changed, the stimulation and recording electrodes were centered at 4 and 15 mm along the nerve such that the default center-to-center conduction distance was 11 mm. The meshed model had 2,093,292 tetrahedral elements. We implemented this baseline model in ASCENT v1.3.0 ([55]; https://github.com/wmglab-duke/ascent; https://doi.org/10.5281/zenodo.10608262), and our dataset includes the JSON files required for reproduction (https://doi.org/10.7924/r4pc3624h).

**Table 3:** Summary of default model parameters. All models used these parameters unless otherwise stated in the caption or in the sensitivity analysis. The default “CV vs. Fiber Diameter Relationships” emerged from the biophysical model (see Nerve Fiber Models section).

Parameter Name and Unit	Value
Conduction Distance (mm)	11
*Tissue Conductivities (S/m)*	
Perineurium	8.7e-4
Endoneurium (longitudinal)	0.57
Endoneurium (radial)	0.17
Surrounding Medium	0.16
*Electrode Properties*	
Recording Electrode Cuff Length (mm)	3.65
Contact Conductivity (S/m)	9.4e6
Cuff Insulator Conductivity (S/m)	1e-12
Cuff Opening (°)	0
Recording Electrode Type	Tripolar
Recording Configuration	Monopolar on Contact 1 relative to Distant Ground
*CV (m/s) vs*. *Fiber Diameter (D*, *in μm) Relationships*	
Myelinated Fibers	CV = 4.01*D—2.5
Unmyelinated Fibers	CV = 0.70*sqrt(D) - 1.9e-3

The purposes of the volume conductor model were (1) to calculate the electric potentials on each compartment of each target nerve fiber due to current from the stimulation electrode and (2) to calculate the electric potentials on the recording electrode due to the current from each compartment of each active nerve fiber (i.e., the *recording sensitivity function*). While the recording sensitivity function can be calculated directly by placing a point current source at each compartment of each active nerve fiber in the finite element model [35,41]—or a representative subset of them [39]—this method is computationally infeasible for fibers with more than a few compartments or for complex electrode geometries. We calculated the recording sensitivity function by leveraging the widely used principle of electromagnetic reciprocity [20,42,56–59], whereby the recording sensitivity function is given by the electric potentials on the nerve fiber due to a unit current applied on the *recording electrode*. Therefore, we placed a point current source within each platinum contact [60] of the stimulation *and* recording electrodes, grounded all outermost surfaces of the models, applied +1 mA through each contact one at a time, and solved for the electric potential due to current at each contact. We used quadratic geometry and solution shape functions, and we used the conjugate gradients solver to solve Laplace’s equation for potentials in the volume assuming quasi-static conditions and non-dispersive materials [61]. We then used superposition to calculate the electric potentials on the nerve fibers due to a bipolar configuration of the stimulation electrode (i.e., potentials from contact 1 relative to potentials from contact 0; Fig 2A). We also calculated potentials due to monopolar configurations of the recording electrode (i.e., potentials from contacts 2, 3, and 4 relative to a distant ground).

### 4.4 Nerve fiber models

Our model nerve contained the number and diameter of fibers present in the left cervical vagus nerve of rat #11327 (female Sprague-Dawley, weight: 198 g, age: 68 days) from a publicly available nerve fiber segmentation dataset [47,62]. We imported the rat #11327 nerve fiber segmentations into Neurolucida 360 software (version 2021; MBF Bioscience, Williston, VT) to calculate all default contour measurements (e.g., min ferret, max ferret, etc.), and then we processed those contour measurements according to the shape-adjusted ellipse calculation [48] to calculate the final nerve fiber diameters. The nerve contained 1,759 myelinated fibers with myelin diameters from 0.33 to 9.81 μm and 13,283 unmyelinated fibers with axon diameters from 0.11 to 1.90 μm. We excluded 83 myelinated fibers due to their diameters <1.01 μm resulting in an invalid modeled ultrastructure (see section on ultrastructure below); we thus modeled 1,676 myelinated fibers.

For the ‘brute force’ approach, we simulated every individual fiber in NEURON. For the efficient ‘filtered interpolation’ method, we modeled in NEURON only 97 unmyelinated fibers with diameters from 0.105 to 1.896 μm in 3.06% increments and 193 myelinated fibers with diameters from 1.013 to 9.809 μm in 1.19% increments. We then simulated transmembrane currents and conduction velocities for these 290 fibers; we interpolated the transmembrane currents to model SFAPs and CNAPs for all fibers in the nerve (see “Filtered Interpolated Action Potential Templates” section below). In one simulation, we randomly positioned the straight nerve fibers across the nerve such that there was no overlap between fibers of a given type (i.e., myelinated, unmyelinated) after accounting for each fiber’s diameter. This positioning produced negligible differences compared to positioning all fibers at the centroid of the fascicle (S1 Text); therefore, we positioned all fibers at the centroid of the nerve for all other simulations to simplify the extraction of electric potentials. All fibers were 30 mm long and centered longitudinally at the center of the recording electrode.

We used MRG (McIntyre-Richard-Grill) myelinated fiber models [63,64] and Tigerholm unmyelinated fiber models [46] implemented in NEURON v7.5 [65], but we redefined the geometric dimensions of myelinated fibers because the original models were not parameterized for small diameters. We fit equations to ultrastructure parameter data from small fiber diameters in the literature (Table 4; see S2 Text for plot of fits, and further details of fitting). Small myelinated fiber models with ion channel conductances based on the original MRG model [63] generated multiple action potentials per stimulus pulse. Therefore, we increased the maximum conductance of voltage-gated potassium ion channels (gkbar = 0.116 S/cm^2^) and decreased the maximum conductance of fast voltage-gated sodium ion channels (gnabar = 2.333 S/cm^2^) to obtain models that fired a single action potential per stimulus pulse (see S3 and S4 Text files). All fibers had passive end nodes to reduce edge effects (g_m_ = 0.0001 S/cm^2^, c_m_ = 2 μF/cm^2^, -70 mV reversal potential).

**Table 4:** Fits or values of ultrastructure parameters for myelinated fibers.

Parameter	Fit or Value
Nodal length^a^	1μm
MYSA length^c,d,e^	3μm
FLUT length^f,g^	$\left(-0.17\frac{1}{\mu m}{\left(D-9.45\;\mu m\right)}^{2}+3.30(D-9.45\;\mu m)+44.85\;\mu m)\times N(1,0.06\right)$
Internodal length^h^	$\left(-2.07\frac{1}{\mu m}{\left(D-9.04\;\mu m\right)}^{2}+87.95(D-9.04\;\mu m)+900.88\;\mu m)\times N(1,0.13\right)$
Internodal axon diameter (d_a_)^i,j^	$(0.020\frac{1}{\mu m}(D-2.39\;\mu m)+0.55+N(0,0.102))D$
Nodal axon diameter (d_n_)^k^	$(-0.011\frac{1}{\mu m}(d_{a}-7.15\;\mu m)+0.4+N(0,0.072))d_{a}$
Number of myelin lamellae (nl)^l^	$exp(0.5\frac{1}{\mu m}(d_{a}-1.75\;\mu m)+3.2+N(0,0.3))$

The independent variables are either fiber diameter (D) in micrometers or internodal axon diameter (d_a_) in micrometers. All expressions evaluate to units of micrometers, except for the number of myelin lamellae. The internodal length is the center-to-center distance between neighboring nodes of Ranvier. The “N(mean,std)” denotes a normally distributed factor with a mean and standard deviation of the fit. See S2 Text for plots of the fits. References: (a) Rydmark and Berthold 1983 [66]; (c)(Berthold 1968a, page 156 [67]; (d) Berthold 1968b, pg 45 [68]; (e) Berthold and Rydmark 1983, pg 966 [69]; (f) Table 1 of McIntyre et al., 2002 [63]; (g) Table 1 of McIntyre et al., 2004 [64]; (h) adult cat data from Fig 3 of Hursh 1939 [70]; (i) Fig 4 of Friede and Samorajski 1967 [71]; (j) Fig 3 of Fazan et al., 1997 [72]; (k) Figs 3 & 4 of Rydmark 1981 [73]; (l) Figs 3A-C of Friede and Samorajski [71].

We stimulated fibers with a supra-threshold symmetric biphasic waveform at t = 2 ms to evoke an action potential in all fibers (Table 5; dt = 10 ms from t = -200 ms to t = 0 ms; dt = 0.0025 ms from t = 0 ms to tstop). For all analyses after the NEURON simulations, we redefined t = 0 ms to be the start of the stimulus pulse. We extracted the net extracellular current from every compartment of every nerve fiber simulated in milliamps by extracting the values of the ‘i_membrane’ variable in NEURON and—in myelinated axons—subtracting periaxonal currents from those values (S5 Text). We stored each transmembrane current time series as an N-by-M matrix containing N time points and M compartments.

**Table 5:** Differences in stimulus waveform and duration between the myelinated and unmyelinated fiber simulations. Both fiber types were stimulated with a symmetric biphasic waveform with 0.005 ms inter-phase delay.

	Myelinated Fibers	Unmyelinated Fibers
tstop (ms)	65	120
phase duration (ms)	0.05	0.2
stimulus amplitude (mA)	-0.43	-0.58

### 4.5 Filtered interpolated action potential templates

#### 4.5.1 Filters

CNAPs are the sum of SFAPs from all fibers in a nerve (Fig 2E), and each SFAP can be computed as the weighted sum of transmembrane currents from every nerve fiber compartment:

$$f(n)=\sum_{m}^{M}i(n,m)w(m)$$ Eq 1

where *f* is an N-by-1 vector containing the SFAP time series (in V); *i* is an *N*-by-*M* matrix containing the transmembrane currents (in mA) generated by *M* compartments of a given type at *N* time points; and *w* is the *M*-by-1 recording sensitivity function (in V/mA) that quantifies the signal produced by compartment *m* per milliamp of transmembrane current. Eq. 1 requires the transmembrane current time series for every compartment, and storing these outputs can produce gigabytes of transmembrane current data *per fiber* depending on number of compartments (i.e., fiber length and fiber discretization) and number of time points (i.e., duration). Therefore, calculating CNAPs across several individuals, nerve targets, or electrodes not only requires running hundreds of thousands to millions of nerve fiber simulations but can also generate gigabytes to terabytes of data collectively. The computational costs of processing, transferring, and storing these data prevent widespread accessibility of CNAP modeling methods.

Fortunately, exploiting redundancies within the transmembrane current traces can reduce simulation burden and data storage needs by orders of magnitude. By assuming that the transmembrane current time series of a given compartment is the same—but time-shifted—as that of any other compartment of the same compartment type, numerous previous authors expressed Eq. 1 as the weighted sum of time-shifted *action potential templates*. We present this expression in discrete time (Eq. 2), convert the time shift into a convolution with a time-shifted unit sample function (Eq. 3), and factor the action potential template out of the summation (Eq. 4) to obtain a numerically efficient expression for calculating SFAPs:

$$f(n)=\sum_{m=1}^{M}i(n-\frac{L}{TV}(m-K),K)w(m)$$ Eq 2

$$f(n)=\sum_{m=1}^{M}i(n,K)*\delta (n-\frac{L}{TV}(m-K))w(m)$$ Eq 3

$$f(n)=i(n,K)*\sum_{m=1}^{M}\delta (n-\frac{L}{TV}(m-K))w(m)=i(n,K)*S(n)$$ Eq 4

where *L* is the distance between two adjacent compartments of the same type; *T* is the sampling period of the signal; *V* is the conduction velocity of the action potential (i.e., CV); *i(n*,*K)* is the action potential template, such that *K* is the index of a selected reference compartment; *ẟ* is the unit sample function; and the filter *S* has the same nonzero values as *w* but with *L/(TV)* zeros inserted between the nonzero elements such that *S* performs all the shifts and scaling operations of the summation. Eq. 2 enables calculation of SFAPs with only a single action potential template per fiber. Eq. 4 efficiently combines the effects of all the time shifts into a single filter operation.

Eq. 2, Eq. 3, and Eq. 4 assume that *T* is selected such that *L/(TV)* is an integer (although replacing *ẟ* with the much less efficient *sinc* function enables use of non-integer values of *L/(TV)* in Eq. 3 and Eq. 4). Further, the sampling rate *1/T* should be greater than twice the Nyquist frequency of the action potential template to avoid aliasing. We set *T* such that *1/T* met these conditions for each individual fiber diameter. While Eq. 4 can be implemented by resampling the action potential template to a sampling period *T* that satisfies the constraints, we transformed the expression into the frequency domain to bypass resampling and to conduct a faster elementwise multiplication in frequency domain rather than a slower convolution in time domain:

$$f(n)=F^{-1}\left[F[i(n,K)]F[\sum_{m=1}^{M}\delta (n-\frac{L}{TV}(m-K))w(m)]\right]=F^{-1}[F[i(n,K))F[S(n)]]$$ Eq 5

where F is the Discrete Fourier Transform (DFT; fft in MATLAB) and F^-1^ is the inverse DFT. Eq. 5 enabled highly efficient reconstruction of SFAPs by transforming the action potential templates and filter into a common frequency resolution prior to elementwise multiplication. Details of our implementation are available in the code that we made available with our dataset.

#### 4.5.2 Multiple compartment types

Myelinated fibers had 11 compartment types per node of Ranvier (i.e., NODE, MYSA1, FLUT1, STIN1, STIN2, STIN3, STIN4, STIN5, STIN6, FLUT2, MYSA2). Eq. 5 applies to such fibers by defining the action potential templates as N-by-M-by-11 matrices and by defining the recording sensitivity function as an M-by-11 matrix:

$$f(n)=\sum_{p=1}^{11}F^{-1}[F[i(n,K,p)]F[\sum_{m=1}^{M}\delta (n-\frac{L}{TV}(m-K))w(m,p)]]$$ Eq 6

where *i(n*,*K*,*p)* is the action potential template of compartment type *p* associated with node of Ranvier *K*, and *w(m*,*p)* is the recording sensitivity function of compartment type *p* associated with the node of Ranvier *m*.

#### 4.5.3 Fiber locations

We obtained the action potential templates from straight fibers placed at the centroid of the modeled nerve. However, we could simulate CNAPs for straight fibers at any xy coordinate of the nerve by sampling the recording sensitivity function, *w*, for each fiber from the volume conductor model. The recording sensitivity function was the only component of Eq. 5 that depended on fiber location, thus the specific location or trajectory of fibers was fully accounted for by the recording sensitivity function sampled along the fiber trajectory.

#### 4.5.4 Interpolation across fiber diameters

Eq. 5 allows highly efficient simulation of many nerve fibers that have the same diameter—and therefore the same action potential template—because their recording sensitivity functions can be summed together to produce a joint recording sensitivity function *w* before applying Eq. 5.

However, each unique fiber diameter requires its own action potential template, resulting in a potentially comparable number of action potential templates to be simulated in NEURON as the brute force method. We bypassed these fiber diameter simulations by taking advantage of the similarity between action potential templates across diameters (Fig 2C) to linearly interpolate action potential templates between fiber diameters (Fig 2E). Given two action potential templates that are aligned so that their max time points match, *i*_*R1*_ and *i*_*R2*_, from fibers with two different fiber diameters, *d*_*1*_ and *d*_*2*_ (where *d*_*1*_ < *d*_*2*_), we interpolated the temporal template from a fiber at an arbitrary fiber diameter, *d*_*i*_ (where *d*_*i*_ > *d*_*1*_ and *d*_*i*_ < *d*_*2*_) with the following weighted average:

$$s_{i}(t)=(\frac{d_{2}-d_{i}}{d_{2}-d_{1}})s_{1}(t)+(\frac{d_{i}-d_{1}}{d_{2}-d_{1}})s_{2}(t)$$ Eq 7

We verified that the method of Eq. 5 reconstructed SFAPs accurately and that the number of templates used for Eq. 7 was adequate to reconstruct SFAPs across the entire fiber diameter range (S6 Text). Finally, to avoid applying Eq. 5 and Eq. 7 repeatedly to fiber diameters that were nearly the same, we summed together the recording sensitivity functions of all nerve fibers that had nearly identical fiber diameters (i.e., within 0.001 μm, based on our sensitivity analyses (S7 Text)).

We recorded action potential templates from compartments that were 75% along the length of the nerve fiber. We saved as a reference the location (in mm) and time (in ms) at which the temporal templates achieved their maximum values, then we defined a new time vector from t = -2 ms to t = 60 ms (unmyelinated) or t = -2.4 ms to t = 36 ms (myelinated) for the action potential template such that t = 0 ms occurred at the maximum value of the temporal template. Since myelinated fiber models were discretized with 11 compartment types, we defined t = 0 ms to be the maximum value of the node of Ranvier’s transmembrane current.

#### 4.5.5 Monopolar vs. Dipolar representations

We encountered unexpected oscillations when interpolating across action potential templates in the smallest myelinated fibers (S8 Text), presumably due to charge imbalances resulting from small errors in CV calculation. This occurred with the *monopolar* representation of nerve fiber compartments in which each compartment was assumed to be a monopolar point source of current. Interpolations for small fiber diameters were more robust to oscillations when we used the *dipolar* representation in which each compartment was assumed to be a point current dipole. Since the monopolar transmembrane current matrix is the negative first spatial difference of the dipolar transmembrane current matrix—indiscriminate of compartment type—we calculated the dipolar transmembrane current matrix as the negative cumulative sum of the monopolar transmembrane current matrix along the spatial dimension for all compartments. Notably, the dipole representation was distinct from dipole approximations of multipoles as is used in EEG and other neural recording applications. Instead, it is an alternative numerical representation of the full transmembrane current spatiotemporal information. Indeed, Plonsey referred to the monopolar and dipolar representations as “single layer disks” and “double layer disks”, respectively, and showed that they are “fully equivalent” [74]. The inherent charge balance of the dipolar representation, however, makes it less susceptible to charge imbalances from CV calculation imprecision. All the equations above apply to either representation except that the dipolar representation uses dipolar action potential templates for *i* and uses the first spatial derivative of the recording sensitivity function for *w*. The code we made available includes the option of either the monopole or dipole representation, and we used the dipolar representation for all simulations.

### 4.6 In Vivo CNAP recordings

We adapted our prior methods [75] to record CNAPs from the cervical vagus nerve of a urethane-anesthetized, paralyzed, ventilated Sprague-Dawley rat (female, 217 g; Charles River Laboratories) (Fig 3). All procedures were approved by the Institute for Animal Care and Use Committee of Duke University (Durham, NC) and were in accordance with the Guide for Care and Use of Laboratory Animals (8th edition). The study was also in compliance with the ARRIVE guidelines [76]. The rat was housed under USDA- and AAALAC-compliant conditions, with 12 h/12 h light/dark cycle and free access to food, water, and environmental enrichment. The rat was anesthetized for 3 min with 3% isoflurane in air to facilitate subcutaneous injection of 1.2 g/kg urethane. We administered a supplemental dose of 0.4 g/kg urethane intraperitoneally 66 min after the initial injection, and then we administered an additional supplemental dose of 0.1 g/kg intramuscularly. We monitored heart rate and blood oxygenation continuously using a pulse oximeter (PalmSAT 2500A; Nonin Medical; Plymouth, MN, USA), and we assessed anesthesia depth using the toe pinch reflex and heart rate. We monitored body temperature using a rectal temperature probe (TH-8 Thermalert; Physitemp Instruments, Inc.; Clifton, NJ) and maintained body temperature between ~35–38°C with a heated water blanket. We delivered 0.05 mg/kg atropine (dissolved in 0.9% saline) intraperitoneally before the surgery to reduce the likelihood of mucus buildup in the trachea. After the experiment, we euthanized the rat via perfusion.

We made a midline neck incision, conducted a tracheotomy to ventilate the rat (PhysioSuite; Kent Scientific; Torrington, CT, USA), and established and maintained paralysis by delivering 20 mg/mL gallamine triethiodide (dissolved in 0.9% saline) every 60 minutes through an intraperitoneal catheter. We then exposed the left carotid sheath via blunt dissection, dissected the cervical vagus nerve from the carotid artery, and placed a bipolar cuff electrode at the rostral end of the nerve and a tripolar cuff electrode at the caudal end (300 μm inner diameter (ID), 1 mm outer diameter, 2580 μm (bipolar) or 3650 μm (tripolar) cuff length, 1070 μm center-to-center contact spacing, 680 μm contact length; platinum-iridium contacts; silicone and polyimide shells; Micro-Leads Neuro, Somerville, MA, USA). We filled the cervical cavity with warm saline. Before implanting each cuff, we removed air bubbles in the cuff by sonicating the cuffs in 70% isopropyl alcohol for 1 min and in saline for 5 min. We then measured the impedance of the electrodes in a saline bath (at 1 kHz and 100 mV), between the two contacts of the bipolar stimulation cuff and between each contact of the recording cuff relative to a needle in the bath. We repeated these measurements after we implanted the cuffs. Recording electrode impedances in vivo were 3.7, 4.7, and 3.5 kΩ on the rostral, middle, and caudal contacts, respectively. During the experiment, we adjusted the conduction distance by moving the stimulation electrode rostrally or caudally to three different conduction distances measured from the center of the stimulation electrode to the center of the recording electrode: 6 mm, 11 mm, 15 mm.

We used bipolar stimulation to evoke maximal CNAPs and conducted monopolar recordings to record CNAPs (B). We used symmetric biphasic waveforms (inter-phase delay: 0 ms; rate: 3.096 Hz) with a phase duration of 50 μs (myelinated fibers) or 200 μs (unmyelinated fibers); the first phase on the caudal contact was cathodic. We also tested the opposite phase to verify that recorded signals were not due to stimulus artifacts. We identified a stimulus amplitude that evoked a maximal CNAP by measuring a dose-response curve; we then used the CNAP evoked by the highest tested stimulation amplitude for comparison to the computational model. For each stimulation amplitude, the stimulus-triggered average CNAP was calculated as the median of the CNAPs evoked by 10 test pulses and recorded differentially by a voltage preamplifier (SR560; Stanford Research Systems; Sunnyvale, CA, USA) with a gain of 200x (conduction distance: 11 mm and 15 mm) or 100x (conduction distance: 6 mm) and analog lowpass filtering (12 dB per octave rolloff at 100 Hz), digitally sampled at 100 kHz (PowerLab 4/35 DAQ; ADInstruments Inc.; Colorado Springs, CO). The output of the stimulus current source was filtered through a circuit identical to that included in our prior studies [75, 77] to avoid undesired DC offsets [78]: 1 μF series capacitors along the positive and negative pathways, a 100 kΩ resistor in parallel with the stimulator, and a 100 kΩ resistor in parallel with the load. The current was delivered through a 1 kΩ resistor in series with the stimulation cuff to monitor stimulus pulse shape and amplitude using a battery-powered oscilloscope (Fluke 190–062 ScopeMeter Test Tool; Fluke Corporation; Everett, WA, USA) to measure the intended amplitude of the stimulus pulses versus the actual amplitude for a range of stimulus pulse amplitudes that were representative of the signals used during the experiment.

## 5 Results

We designed, implemented, and applied an efficient method to model CNAPs from a nerve containing tens of thousands of nerve fibers. We quantified recording sensitivity functions from numerically solved volume conductor models using the reciprocity principle [20,42,43]. We simulated the full transmembrane current matrix in a subset of fiber diameters to extract ‘action potential templates’ (i.e., transmembrane current time series at the compartments at and around a single node) as done in [41] and applied a new method of interpolating across the templates. We calculated SFAPs by filtering interpolated templates using a weighting function derived from the volume conductor model and fiber-specific CVs, similar to [44]. We calculated CNAPs using this highly efficient method and demonstrated that it reproduced brute force simulations accurately. We then applied this method to conduct a comprehensive sensitivity analysis that revealed large effects of volume conductor tissue parameters, conduction distance, and fiber diameter distribution on the CNAP amplitude, latency, and shape. Comparison of model outputs to an in vivo CNAP showed good agreement in amplitude and shape with myelinated fibers but not unmyelinated fibers.

### 5.1 Filtering interpolated templates produced CNAPs that matched brute force simulations

**Fig 4:**
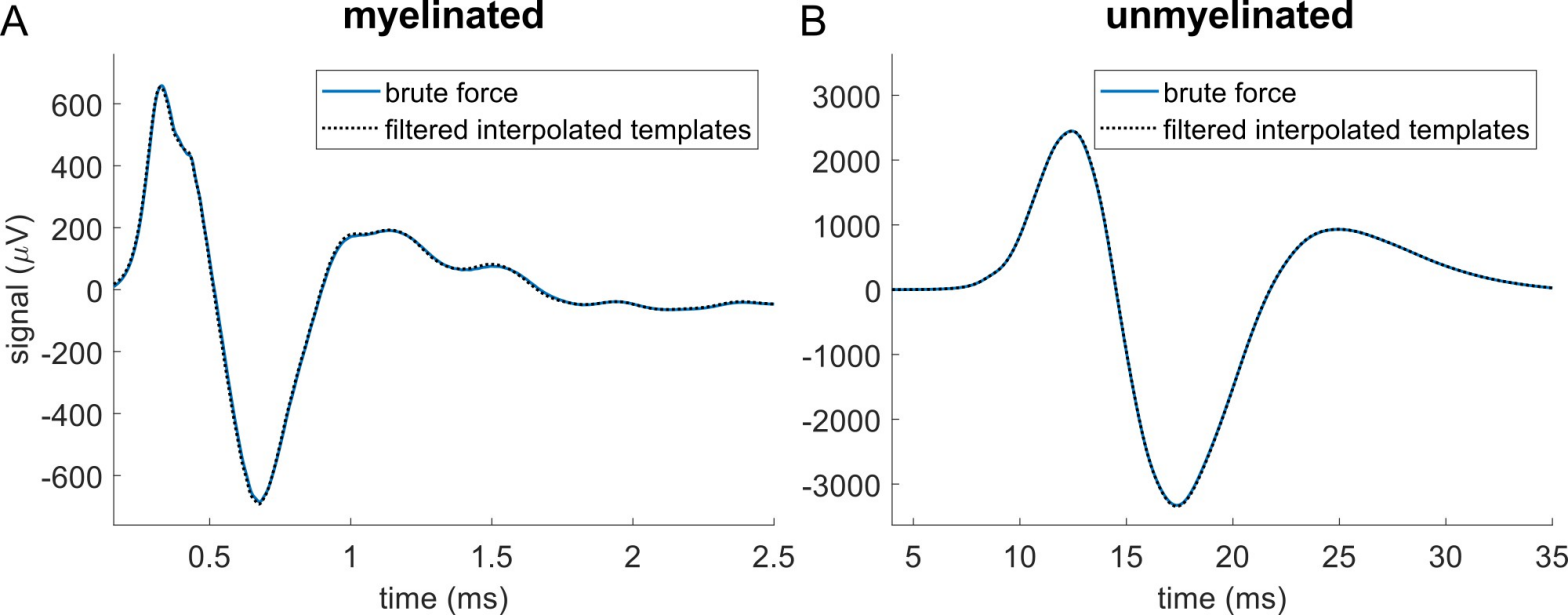
Modeled CNAPs for myelinated (A) and unmyelinated (B) fibers in rat cervical vagus nerve calculated by using brute force (blue solid) or by filtering interpolated templates (black dotted). In the brute force method, we simulated all 1,759 myelinated fibers and 13,283 unmyelinated fibers in NEURON, while in the ‘filtering interpolated templates’ method, we simulated only 193 myelinated fibers and 97 unmyelinated fibers and interpolated templates for the thousands of remaining fibers.

The brute force method used NEURON to simulate all 1,759 myelinated fibers and 13,283 unmyelinated fibers in a rat cervical vagus nerve, and we compared the resulting CNAPs to signals calculated using our template method. The template method simulated only 193 myelinated and 97 unmyelinated fibers in NEURON, spanning the range of fiber diameters: we extracted the action potential templates for each fiber, interpolated templates for the thousands of remaining fibers, and convolved each template with fiber-specific filters calculated from the volume conductor model and CV to generate SFAPs. The resulting CNAPs reproduced the brute force results accurately (Fig 4).

After obtaining the electrodes’ recording sensitivity functions from the volume conductor, the time required to reconstruct the CNAP by filtering interpolated action potential templates was 38 seconds for myelinated fibers and 30 seconds for unmyelinated fibers on a desktop without using parallelization (processor: AMD Ryzen 7 1700 Eight-Core Processor 3.00 GHz; RAM: 16 GB). In contrast, the brute force method that simulated every nerve fiber required approximately 48 hours when parallelized across hundreds of CPUs on the Duke Compute Cluster, corresponding to approximately 1,030,000 CPU seconds (286 CPU hours) for myelinated fibers and 57,000,000 CPU seconds (15,860 CPU hours) for unmyelinated fibers. Thus, our efficient method achieved 27,000–1,900,000x speedup. This speedup was achieved through two primary approaches (see Methods for details): (1) interpolating transmembrane currents across fiber diameters, and (2) calculating filters via inserting zeros into the recording sensitivity function and then transforming the filters into the frequency domain at appropriate frequency resolution to conduct elementwise multiplication with the transmembrane current templates. While the new method did initially require 193 myelinated and 97 unmyelinated fiber simulations, these simulations were run only once and were used as the bases for all subsequent analyses, including parameter sensitivities.

**Fig 5:**
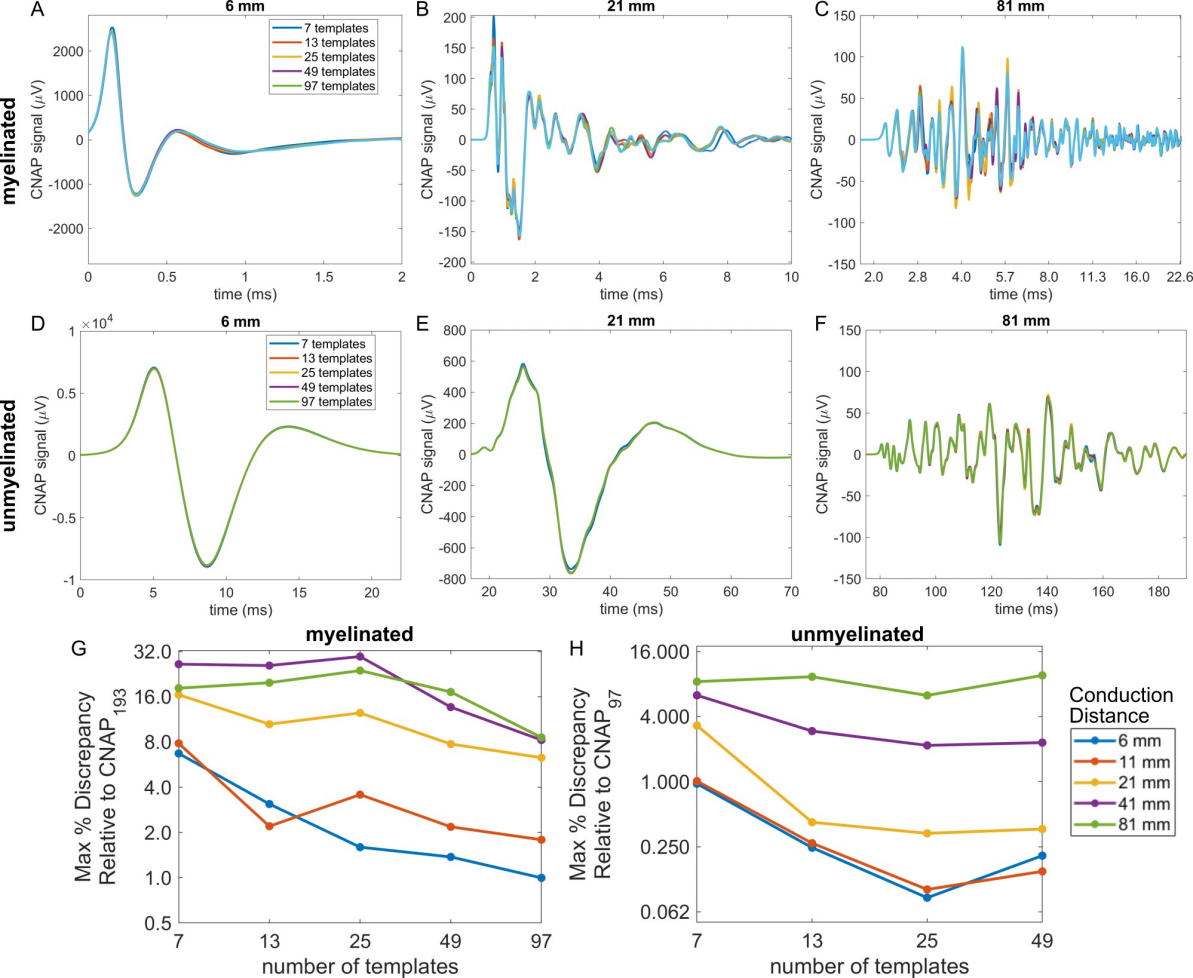
CNAPs modeled with different numbers of templates by using linear interpolation across fiber diameters (1.013 to 9.809 μm for myelinated fibers and 0.105 to 1.896 μm for unmyelinated fibers) at five conduction distances. (A-F) Example myelinated and unmyelinated CNAPs at conduction distances of 6, 21, and 81 mm. (G-H) Maximum percent discrepancy between CNAP_n_ (i.e., CNAP constructed from n templates) and CNAP_finest_ (i.e., finest = 193 for myelinated or finest = 97 for unmyelinated): 100*max(abs(CNAP_n_—CNAP_finest_))/V_pk-pk,finest_.

The accuracy of CNAP modeling depended on the number of templates, conduction distance, and—most of all—the interpolation method. Decreasing the number of fiber diameters simulated to obtain action potential templates slightly altered the amplitude of CNAPs, and this effect was more pronounced for longer conduction distances (Fig 5). However, the timing, shape, and overall amplitude of the CNAPs remained relatively consistent irrespective of the number of templates used for both myelinated and unmyelinated fiber CNAPs (Fig 5A–5F). Using a binning approach that did not interpolate linearly but instead grouped fiber diameters into bins equal to the number of templates (i.e., nearest neighbor interpolation) [41] resulted in large oscillations and deviations in shape and amplitude of the CNAPs across both myelinated and unmyelinated fibers (S9 Text).

### 5.2 Effect of tissue conductivity values and cuff opening

#### 5.2.1 Effects on CNAP amplitude

**Fig 6:**
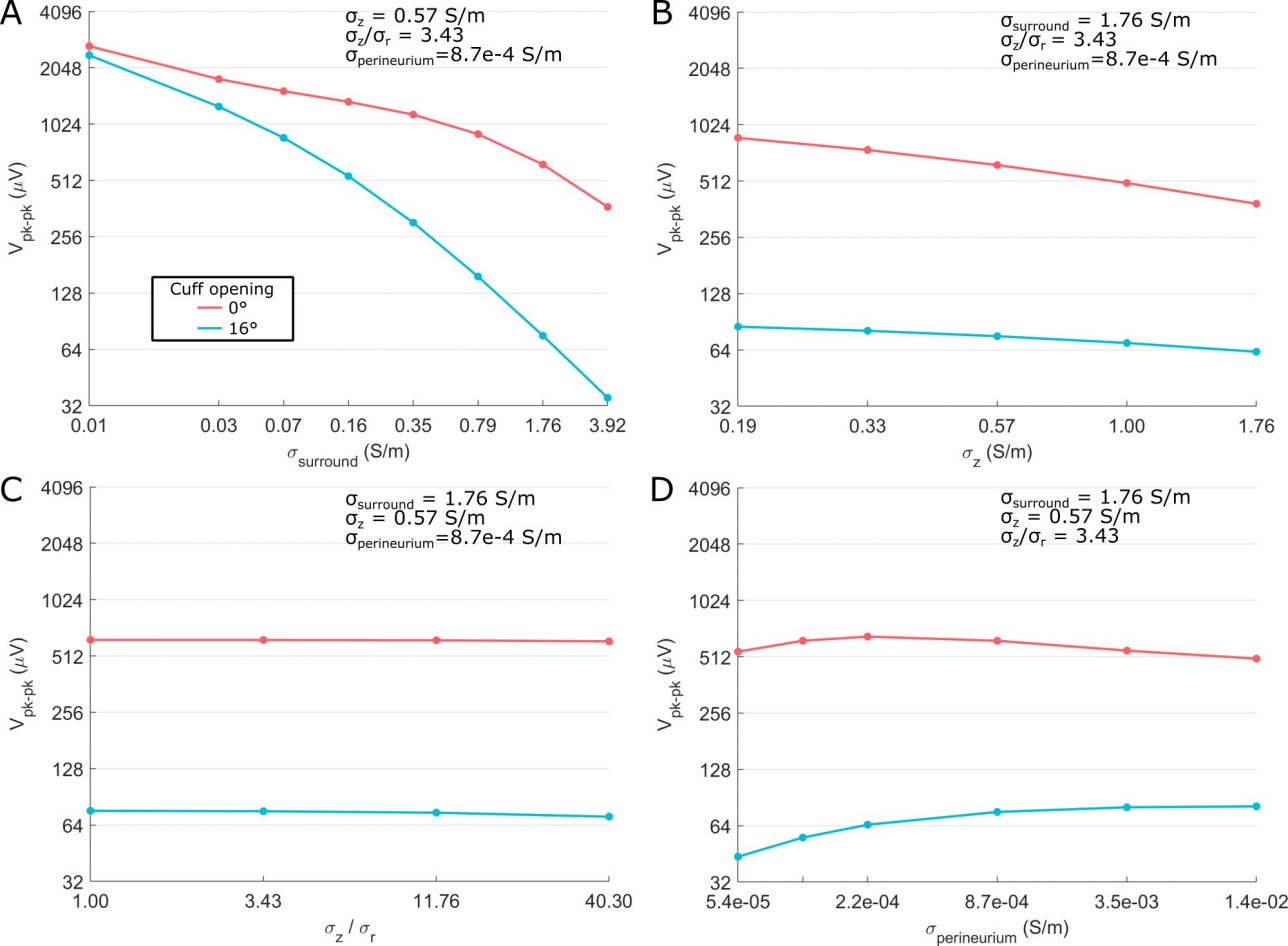
Sensitivity of peak-to-peak CNAP amplitude (V_pk-pk_) across tissue conductivity values and cuff opening size on the myelinated fiber CNAP from the rat cervical vagus nerve at a single conduction distance (11 mm center-to-center). ‘σ_surround_’ is the conductivity of the cuff slit, of the medium containing the cuff and nerve, and of the thin space between the cuff and the nerve (Fig 2A); ‘σ_r_’ and ‘σ_z_’ are the radial and longitudinal conductivity of the endoneurium, respectively; ‘σ_perineurium_’ is the conductivity of the perineurium.

Tissue conductivities could strongly influence CNAP amplitude. With a fully sealed cuff (0° opening), the largest effect was due to surround conductivity, which comprised the conductivity of the cuff slit, the medium containing the cuff and nerve, and the thin space between the cuff and nerve (Fig 2A). Surround conductivity changed CNAP amplitude by >2x across a biologically plausible range of conductivities: 0.03 S/m (fat) to 1.76 S/m (saline) (Fig 6A). Increasing endoneurial longitudinal conductivity over a large range, i.e., from one-third to three times the published value of 0.57 S/m, decreased peak-to-peak CNAP amplitude by ~2x (Fig 6B). Endoneurial anisotropy and perineurial conductivity had the smallest effects even when sweeping their values over a substantial range around their literature values of 3.43 (endoneurial anisotropy; Fig 6C) and 8.7e-4 S/m (perineurium conductivity; Fig 6D). There were two interaction effects between tissue conductivities: perineurium conductivity had a larger effect at intermediate values of surround conductivity; surround conductivity had a larger effect at smaller endoneurium longitudinal conductivities (S10 Text). Results for unmyelinated fibers were comparable to myelinated fibers (S11 and S12 Text files).

Compared to the fully sealed cuff, using a cuff with a 16° slit (“non-sealed cuff”) reduced CNAP amplitudes by 4x or 8x at typical surround conductivities of 0.35 S/m (e.g., longitudinal muscle conductivity) or 1.76 S/m (e.g., saline), respectively (Fig 6A). Thus, the expected reduction of CNAP amplitude with non-sealed cuffs [20] was stronger when the surrounding medium was more conductive. Conversely, as the surrounding medium was made less conductive (e.g., 0.01 S/m), the cuff opening effects approached zero. The 16° slit strengthened the amplitude effects of surround conductivity (Fig 6A), weakened the amplitude effects of longitudinal endoneurial conductivity (Fig 6B), and strengthened the effects of perineurium conductivity such that CNAP amplitude generally decreased as perineurium conductivity increased (Fig 6D). Results in unmyelinated fibers were comparable to myelinated fibers (S12, S13, and S14 Text files).

#### 5.2.2 Effects on CNAP shape

**Fig 7:**
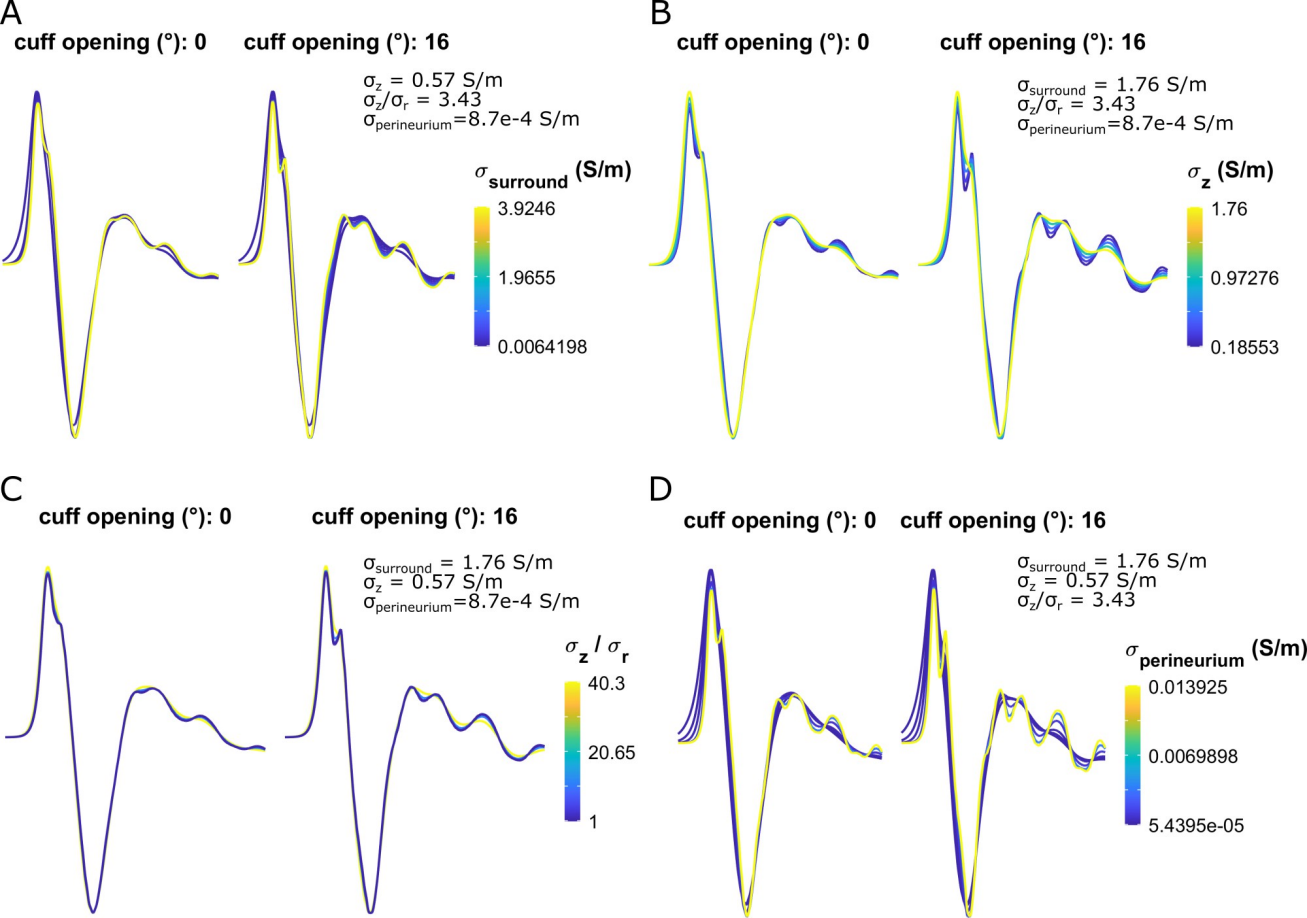
Sensitivity of myelinated fiber CNAP shape to all tissue conductivities and cuff openings shown in Fig 6. Each subpanel shows a normalized waveform (to facilitate shape comparison) from t = 0 to t = 2 ms.

Tissue conductivities had modest effects on waveform shape. In sealed cuffs, CNAP shape was only slightly smoother at higher longitudinal endoneurial conductivities (Fig 7B) and lower perineurium conductivities (Fig 7D). Surround conductivity and endoneurial anisotropy had a negligible effect on CNAP shape (Fig 7A and 7C). Non-sealed cuffs slightly intensified the conductivity-driven effects on CNAP shape, but the effects remained modest (Fig 7). Results in unmyelinated fibers were comparable to myelinated fibers (S15 Text)).

### 5.3 Effect of conduction distance

**Fig 8:**
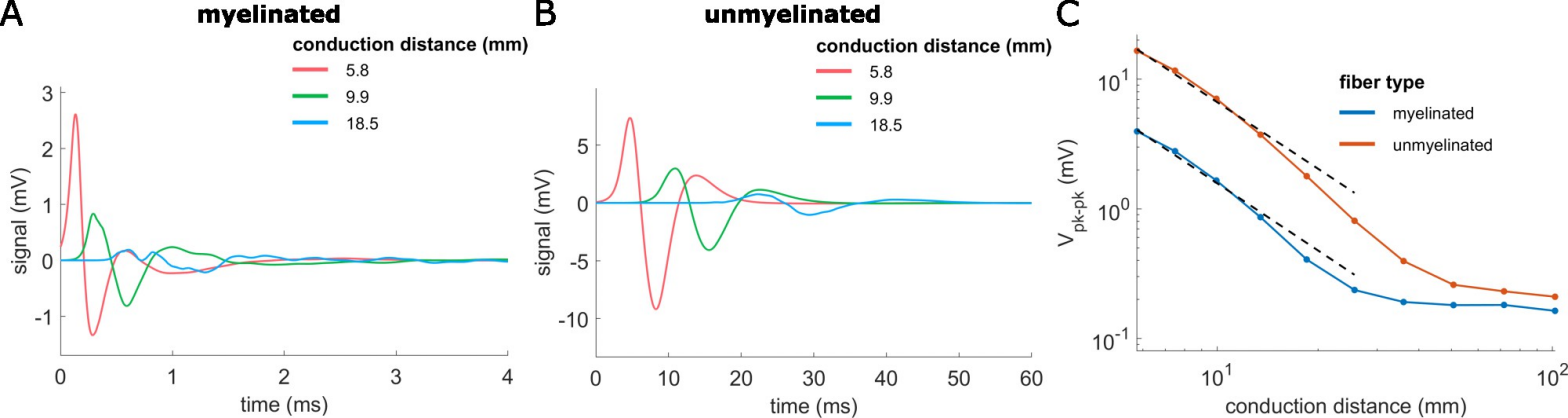
Effects of conduction distance on CNAPs from rat cervical vagus nerve. CNAPs from myelinated (A) and unmyelinated (B) fiber populations for different conduction distances (center-to-center) between the stimulation and recording cuffs. (C) Effect of conduction distance (5.8 to 101.8 mm) on peak-to-peak CNAP amplitude for myelinated and unmyelinated fibers. Nonlinear power fits of conduction distances <26 mm (black dashed lines) related amplitude (in mV) to conduction distance (in mm): amplitude = 83*distance^-1.7 (myelinated), amplitude = 339*distance^-1.7 (unmyelinated).

Increasing the conduction distance from 5.8 mm to 101.8 mm (center-to-center between stimulation and recording cuffs) decreased CNAP amplitude, increased CNAP latency, and altered the shape of signals from both myelinated and unmyelinated fibers (Fig 8). CNAP shape changed as the conduction distance increased such that the first positive peak of myelinated fiber CNAPs was larger than the first negative peak when conduction distance was 5.8 mm, but comparable to the negative peak at longer conduction distances (Fig 8A). Unmyelinated fiber CNAPs always had smaller first positive peaks than first negative peaks (Fig 8B). Within the feasible length of a rat cervical vagus nerve (i.e., ~26 mm), the signal amplitude decreased approximately as the inverse square of the conduction distance (power fits for distances <26 mm: amplitude = 83*distance^-1.7 (myelinated), amplitude = 339*distance^-1.7 (unmyelinated)).

### 5.4 Effect of nerve fiber properties

#### 5.4.1 Fixed Bins vs. Random sampling of fiber distributions

**Fig 9:**
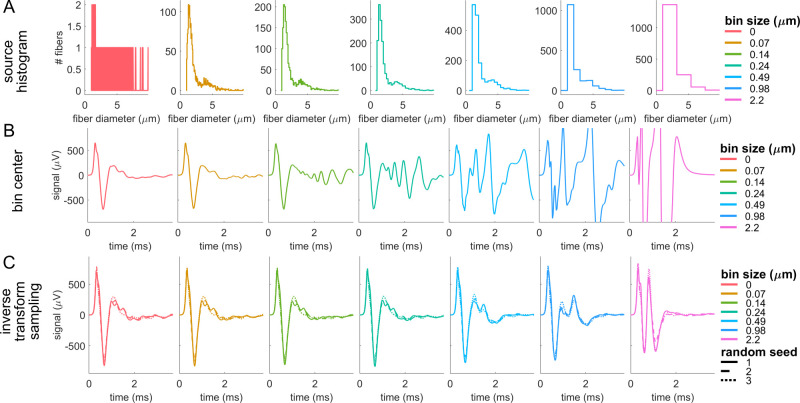
Effect of bin size and sampling method on myelinated fiber CNAPs during extraction of fiber diameters from distributions. (A) Histograms of known myelinated fiber diameters across different bin sizes. A bin size of 0 μm used the individual fiber diameter measurements (precision of 1e-6 μm). (B) Effect on CNAPs of generating fiber diameters based on the center of the bin and the bin height. As bin size increased, using the bin centers produced inaccuracies due to less destructive interference and more constructive interference. (C) Effect on CNAPs of generating fiber diameters based on inverse transform sampling to sample diameters randomly from the estimated cumulative distribution function. For a given non-zero bin size, CNAPs were more accurate than when using bin centers.

CNAPs could be reconstructed from fiber diameter distributions rather than from known specific fiber diameters. However, the bin width used to quantify the distributions and the method of sampling the distributions had large effects on the CNAPs. Quantifying fiber diameter distributions using different bin widths (Fig 9A and S16 Text) and using the bin centers for calculating CNAPs produced signals with accuracy that decreased as the bin width increased (Fig 9B and S16 Text). Using standard inverse transform sampling to randomly sample fibers from the cumulative distribution function of the distributions was more accurate than using the bin centers (Fig 9C and S16 Text vs. Fig 9B and S16 Text). The necessary resolution for fiber diameter distribution quantification to construct CNAPs accurately was at least 0.24 μm (myelinated) or 0.14 μm (unmyelinated) using inverse transform sampling. Although inverse transform sampling is a stochastic sampling process, the resulting CNAPs were quite consistent across random seeds (Fig 9C and S16 Text).

#### 5.4.2 Fiber diameter distributions in literature

**Fig 10:**
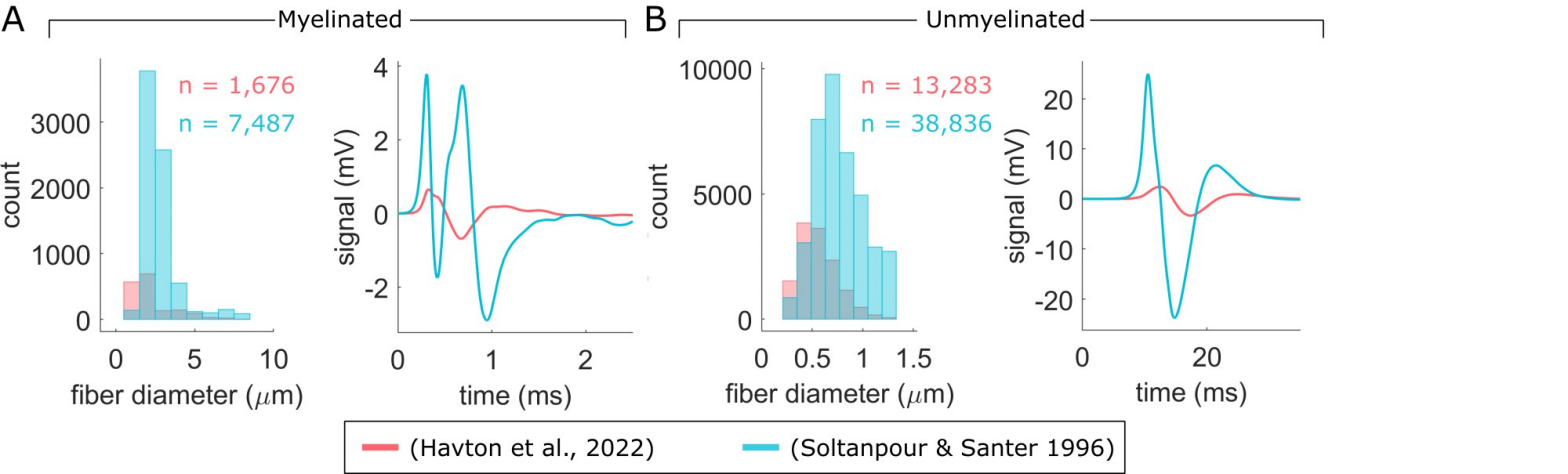
Comparison of CNAPs from two published fiber diameter datasets of myelinated (A) and unmyelinated (B) fibers in rat cervical vagus nerve. Individual fiber measurements from the (Havton et al., 2022) dataset [47] were from a left cervical vagus nerve (sex: female; age: 68 days; strain: Sprague-Dawley; weight: 198 g); we corrected the measurements according to the shape-adjusted ellipse method [48]. Fiber diameter distributions from (Soltanpour & Santer 1996) [79] were from a right cervical vagus nerve (sex: male; age: 4 months; strain: Wistar; weight: none listed); we used standard inverse transform sampling to obtain individual fiber measurements.

We evaluated the effect of fiber diameter distribution by calculating CNAPs using two sources of rat cervical vagus nerve fiber diameter measurements [47,79]. Soltanpour & Santer [79] reported a larger number of fibers and generally larger fiber diameters than Havton and colleagues [47], resulting in substantially larger CNAPs with shorter latencies (Fig 10).

#### 5.4.3 Effect of CV vs. Fiber diameter

**Fig 11:**
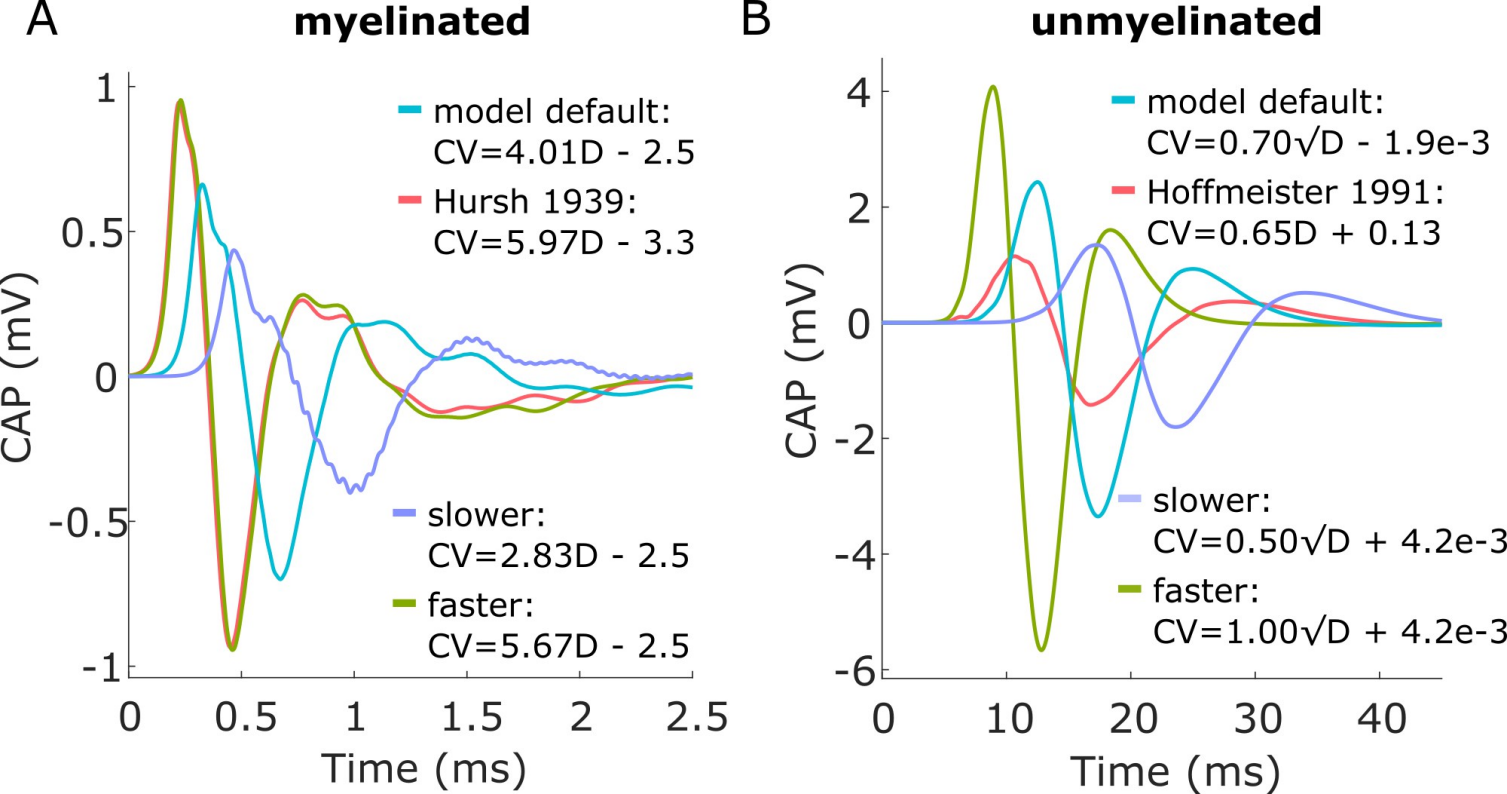
Effect of CV-to-fiber diameter relationship on CNAPs from myelinated fibers (A) and unmyelinated fibers (B).

The CV vs. fiber diameter relationship had a large effect on CNAP shape, amplitude, and latency. Further, CV vs. fiber diameter measurements from literature differed from those derived from biophysical models, resulting in notably different modeled CNAPs. The CV of modeled fibers was linearly related to fiber diameter in myelinated fibers (i.e., CV = m*D + b; S17 Text) and linearly related to the square root of fiber diameter in unmyelinated fibers (i.e., CV = m*sqrt(D) + b; S17 Text). Increasing the ‘slope’ coefficient, m, of these relationships resulted in larger CNAP amplitudes and shorter CNAP latencies, and reducing the coefficient had the opposite effect (Fig 11). Hursh reported a linear relationship between CV and myelinated fiber diameter in somatic and autonomic cat nerves [70], and CV values were faster than those of modeled myelinated fibers (S18 Text). CNAPs using the Hursh data had larger amplitude and shorter latency than the default model, comparable to the CNAP produced with an increased ‘slope’ coefficient (Fig 11A). Hoffmeister and colleagues reported a linear relationship between CV and unmyelinated fiber diameter in somatic cat nerves [80], and CV values were comparable to those of modeled unmyelinated fibers (S18 Text). CNAPs using the Hoffmeister data had comparable latency and shape to the default model, but CNAP amplitude was lower (Fig 11B).

#### 5.4.4 Effects of fiber shrinkage

**Fig 12:**
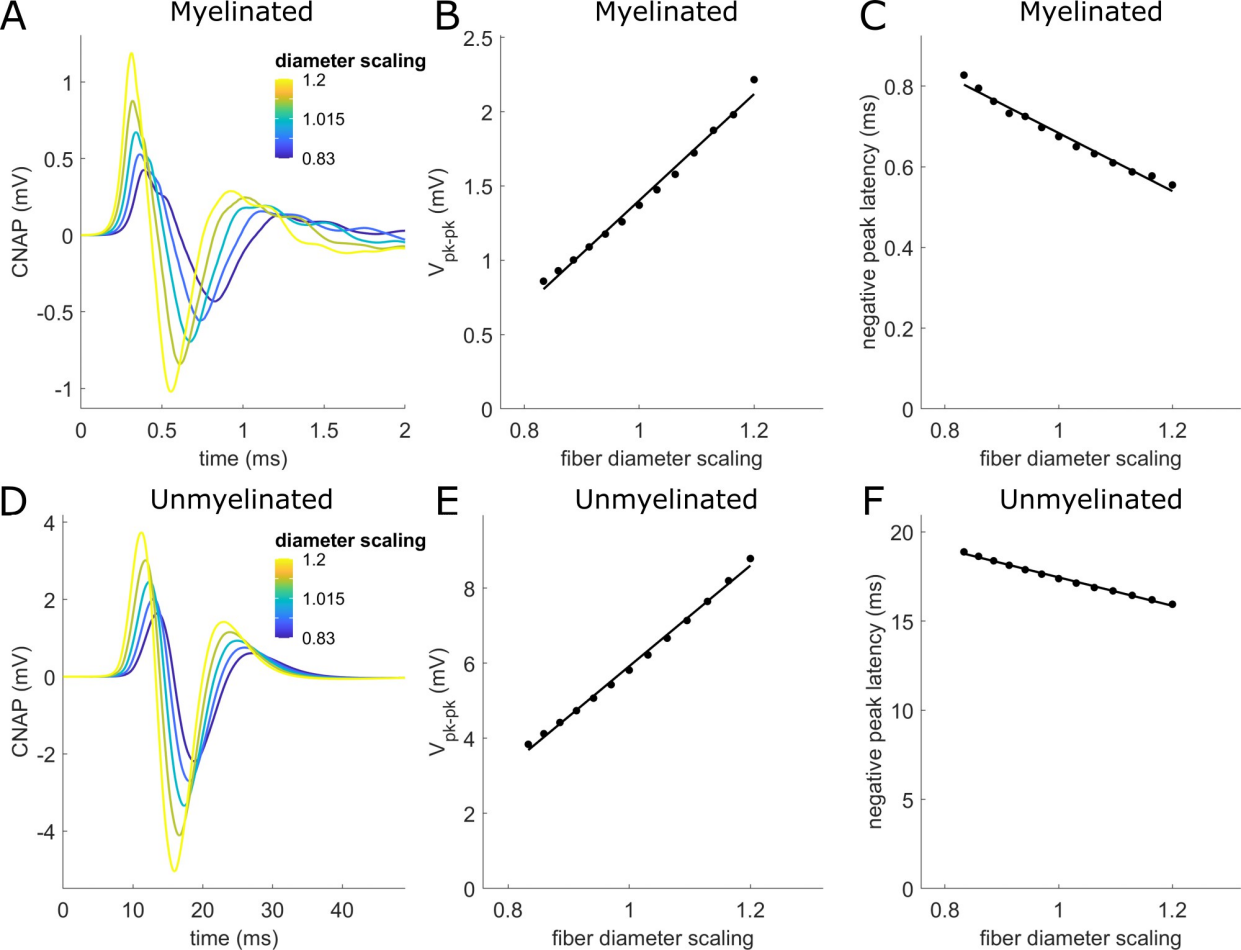
Effect on CNAPs due to scaling fiber diameters. (A,D) CNAPs from myelinated (A) and unmyelinated (D) fibers with diameters scaled by different scaling factors (legend). (B,E) Effects of diameter scaling factor on peak-to-peak CNAP amplitude. (C,F) Effects of diameter scaling factor on latency of the CNAP negative peak. Linear fits (black solid lines) related V_pk-pk_ or latency (y) to fiber diameter scaling (x) with an adjusted R^2^ value ≥0.98 for all fits: (B) y = -2.18 + 3.58*x; (C) y = 1.40–0.72*x; (E) y = -7.48 + 13.40*x; (F) y = 25.41–7.97*x.

Tissue fixation, dehydration, and other histological processing can shrink the observed nerve fiber diameters by variable amounts, e.g., 10.1±0.16% [70], 6–8% [81], 20% [82], and 25–50% [71]. CNAP peak-to-peak amplitude increased by 26% (myelinated) or 23% (unmyelinated) per 10% increase in fiber diameter (Fig 12). CNAP latency shortened by 11% (myelinated) or 5% (unmyelinated) per 10% increase in fiber diameter.

#### 5.4.5 Fiber XY location and Z jitter had a negligible effect on CNAPs

Fiber location within the cross section of the monofascicular rat cervical vagus nerve had a negligible effect on CNAP amplitude, shape, and latency (S1 Text). Randomly displacing the longitudinal positions (i.e., z-coordinate) of each myelinated nerve fiber individually by a factor of ±0.5 times the fiber-specific internodal distance also had only a minor effect on signal shape and amplitude but no effect on latency (S19 Text).

### 5.5 Comparison to In Vivo recording

**Fig 13:**
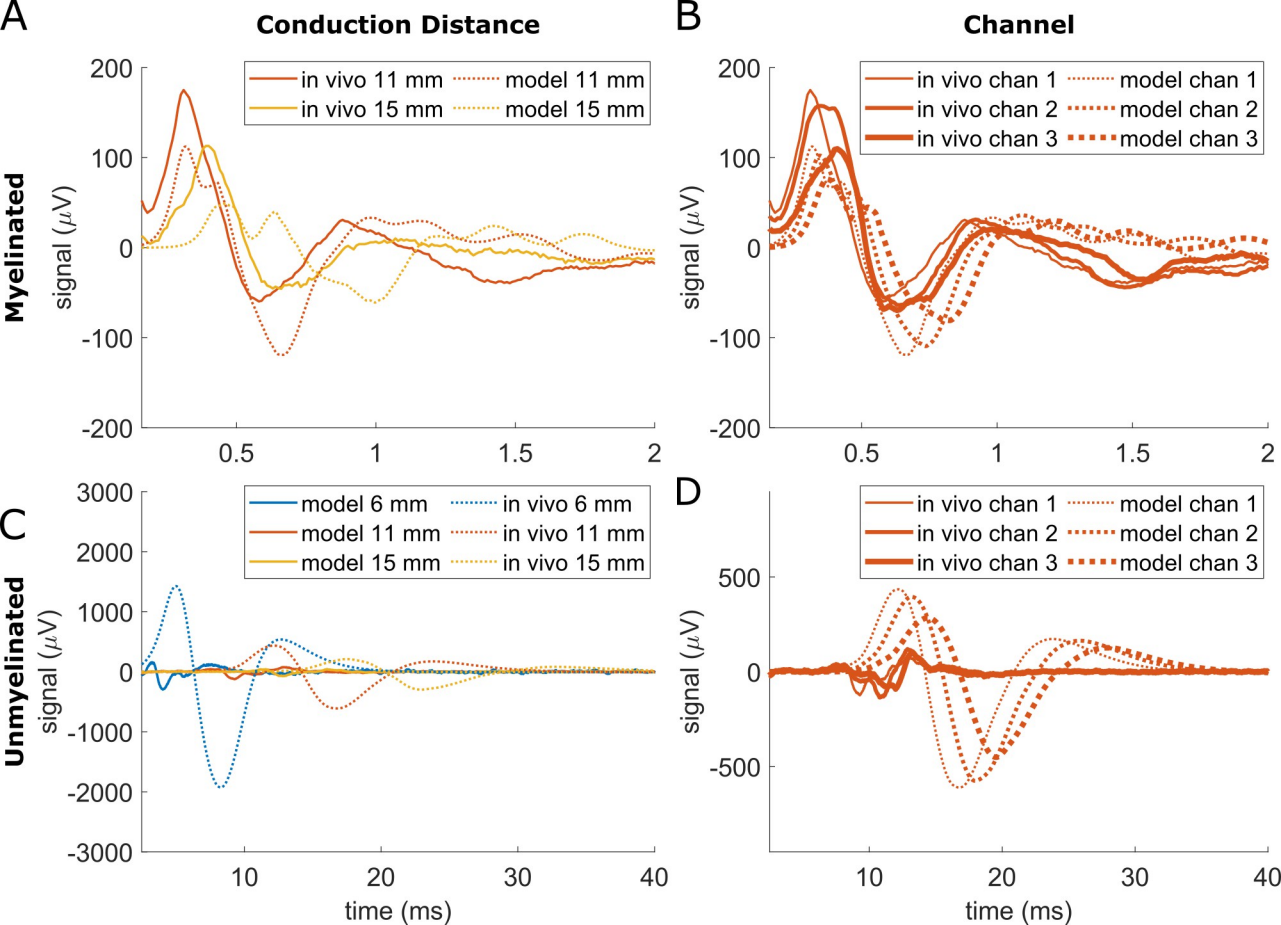
Comparison of CNAPs from in vivo data (solid lines) and models (dashed lines) from a rat cervical vagus nerve across conduction distances (A,C) and recording channels within the tripolar cuff (B,D). Model surround conductivity and cuff opening were tuned to 0.50 S/m and 16°, respectively, to match the model amplitude to the in vivo myelinated fiber CNAP at 11 mm conduction distance from recording channel 1.

In vivo CNAPs had markedly smaller amplitudes than modeled CNAPs in initial models using conductivities from literature, a 0° cuff opening, and a surround conductivity of 0.16 S/m (i.e., epineurium) (Fig 1). Therefore, we tuned our models’ surround conductivity and cuff opening—the parameters that are most likely to vary experimentally and that had the greatest effect based on our sensitivity analysis (Fig 6)—to 0.50 S/m and 16°, respectively, to match the model amplitude to the in vivo myelinated fiber CNAP in Fig 1A. The resulting modeled myelinated fiber CNAPs had comparable amplitude to in vivo recordings across all conduction distances, all recording channels, and both stimulation polarities, within a factor of 1.4 (Fig 13A and 13B). However, the unmyelinated fiber CNAPs were still 4–7 times smaller in vivo compared to the model (Fig 13C and 13D). The modeled latency of the negative peak was longer than in vivo by 1.2–1.5x for myelinated fibers and by 1.8-2x for unmyelinated fibers. All CNAPs except the in vivo unmyelinated CNAPs had a triphasic shape (Fig 13C and 13D).

We examined the effects of conduction distance and recording channel from a tripolar cuff. Increasing conduction distance decreased CNAP amplitudes and lengthened CNAP latencies by a larger amount in the model than in vivo (S20 Text). Changing the recording channel had a similar qualitative effect in vivo and in models; CNAPs generally had lower amplitudes and slower latencies for recording contacts further from the stimulation cuff.

## 6 Discussion

### 6.1 Filtered interpolation method

Computational models have great potential to facilitate the design of electrodes that can extract information selectively from the peripheral nervous system to guide next-generation, closed-loop stimulation therapies and to enhance understanding of physiological systems. However, there is a need for (1) rigorous computational models of nerve recording; (2) model validation; (3) increased efficiency of simulations because calculating recordings for the tens of thousands of fibers in a nerve is computationally prohibitive. Filtering action potential templates produced CNAPs that matched the brute force approach with negligible error in shape, amplitude, and latencies across unmyelinated and myelinated fibers. Filtering action potentials achieved these results in orders of magnitude less time than the brute force method of using transmembrane current matrices (30 seconds and 38 seconds compared to 286 and 15,860 CPU hours for myelinated and unmyelinated fibers) for simulating the CNAPs of a rat cervical vagus nerve. This computational efficiency is even more crucial for larger species and nerves, such as human cervical vagus nerves that contain tens of thousands of myelinated fibers (mean: 19,770 [83]; mean: 16,552 [84]) and unmyelinated fibers (mean: 92,214 [83]). Thus, interpolating action potential templates of transmembrane currents from a relatively small number of pre-computed action potential templates makes CNAP modeling accessible for application in a variety of clinical and research settings.

Filtering action potential templates enhanced the efficiency of SFAP and CNAP calculation by exploiting action potential redundancies over space within a fiber and similarities across different fiber diameters. This method is expected to be valid for large populations of fibers of a given kind (e.g., A-fibers, B-fibers, and C-fibers separately) but is not valid when using templates of one kind of fiber for another kind of fiber (e.g., A-fiber templates for B-fiber or C-fiber modeling) due to differences in action potential templates from those fibers at a given diameter. Similarly, any two specific fibers may have slight differences in ultrastructure or ion channel composition that could result in distinct action potential templates that are not accounted for by filtering action potentials, but such differences are assumed to average over the entire population. We interpolated *transmembrane currents* (linearly)—rather than *SFAPs* (nonlinearly via stretching or contracting)—across fiber diameters because interpolating SFAPs requires recalculating SFAPs for different volume conductors, fiber locations, and fiber trajectories. Further, we used *temporal* action potential templates (i.e., *i(n*,*K)* from Eq. 4, where *n* is all time point indices and *K* is a reference compartment) rather than *spatial* action potential templates (i.e., *i(J*,*m)*, where *J* is a reference time point and *m* is all compartment indices); interpolating spatial templates across fiber diameters (nonlinearly via stretching or contracting) is challenging when there are multiple compartment types that differ in spacing across fiber diameters (e.g., as in myelinated fibers).

### 6.2 Tissue conductivity and cuff opening effects

Our study extends previous literature by quantifying the influence of tissue conductivities on CNAPs over a biologically feasible range of values. The observed effects on CNAP amplitude were substantial, and these observations have implications for model-based signal prediction and recording interface design. Our findings suggest that model-based predictions of CNAP amplitudes are subject to uncertainty on the order of 2x-8x due to uncertainties of tissue conductivities and cuff opening. The fact that surround conductivity and cuff seal were the most influential parameters highlights a key opportunity: techniques that control or probe the cuff seal and surrounding medium conductivity in situ would improve the predictive accuracy of models by eliminating the primary sources of volume conductor uncertainty. The absence of latency effects is expected because purely resistive volume conductors do not alter signal propagation delay. Tissue conductivities and cuff seal had almost no effect on CNAP shape, even in a model with perineurium removed (S23 Text). The implication for predictive models of extraneural recording is that efforts to account for temporal properties of CNAPs (e.g., shape, latency) should focus on other parameters that influence these properties more heavily (e.g., conduction distance, fiber parameters).

The effects of surround conductivity and cuff seal on CNAP amplitude are consistent with previous studies and first principles. The marked decrease in CNAP amplitude with increasing conductivity of the surrounding medium follows from the application of Ohm’s law to a current-controlled source such as transmembrane current (i.e., decreasing resistance, R, for a fixed current, I, causes voltage, V, to decrease as well). Previous analyses formalized this concept for point or ring electrodes in homogeneous isotropic media of conductivity σ, showing that SFAP amplitude is proportional to 1/σ [21,74]. These concepts also explain the substantial and well-established effect of cuff seal in maintaining a high amplitude by keeping a low effective tissue conductivity around the nerve [9,20].

The non-monotonic effects of perineurium conductivity in a fully sealed cuff indicate a tradeoff that may be explained in terms of current leakage. The perineurium reduces the amount of current exiting the nerve, thus decreasing the recorded signal amplitude within the cuff. However, when the cuff is well-insulated (e.g., intermediate surround conductivity & fully sealed cuff), the perineurium simultaneously prevents current from *leaking* into the space outside the cuff, thus increasing the recorded signal amplitude within the cuff. As the cuff’s insulating capabilities decrease (e.g., high surround conductivity & non-sealed cuff), current leakage happens at the cuff itself and therefore decreasing the perineurium conductivity only reduces the amount of current reaching the recording electrode, decreasing recorded signal amplitude. These findings indicate that the effect of perineurium depends on the recording environment. Indeed, previous analytical models of media with inhomogeneities showed that a material’s effects depended on its conductivity relative to that of other materials [21]. The default perineurium conductivity of 8.7e-4 S/m (resistivity of 1149 Ω-m) was measured from amphibian somatic nerves [51, 85]. More recent measurements from canine vagus nerves identified an average conductivity of 2.7e-4 S/m (resistivity of 3751 Ω-m) [86], which was ~3-fold lower than the measurements from amphibian somatic nerves. We varied the default perineurium conductivity by factors of 1/16 to 16x, and the effects on CNAP amplitude and waveform were still limited. Therefore, we expect that the perineurium conductivity within a biologically feasible range does not substantially impact recordings from rat cervical vagus nerve.

Endoneurial longitudinal conductivity in our models had the opposite effect to a study by Wijesinghe and colleagues [25], although that study did not include a perineurium, so the tradeoff of conductivity effects likely differed. Anisotropy had a negligible effect in our models in contrast to Wijesinghe and colleagues [25]; however, it is unclear how that previous study altered anisotropy (i.e., whether radial conductivity was changed while maintaining longitudinal conductivity, or vice versa).

### 6.3 Nerve fiber effects

#### 6.3.1 Fiber diameter distribution

Fiber diameter quantification and sampling methods had a large influence on CNAP shape, amplitude, and latency. Fiber diameter data are traditionally quantified in the literature with histograms, and sampling the bin center of such histograms can lead to large artifacts in CNAPs due to excessive constructive interference between the fibers. On the other hand, stochastic sampling to obtain individual fiber diameters from histograms (e.g., via inverse transform sampling) produced CNAPs that resembled CNAPs calculated using the known individual nerve fiber diameters. Therefore, while measurements of individual nerve fibers are limited in the literature, sufficiently fine histogram quantification may still enable accurate CNAP reconstructions. Nevertheless, there are limited such data available in the literature, and even fewer compare distributions across individuals. Indeed, fiber diameter distributions from the only two literature sources of myelinated and unmyelinated fiber diameter measurements from rat cervical vagus nerve [47,79] had a marked effect on CNAP amplitude and shape. Inter-individual differences included fiber count (n_total_ = 9,163 [47] vs. n_total_ = 52,119 [79]), rat age and gender (60-day-old female [47] vs. 4-month-old male [79]), analysis method (i.e., correction of Havton et al., 2022 data [47] with the shape-adjusted ellipse method [48]), and mode fiber diameter (1.72 μm (myelinated), 0.53 μm (unmyelinated) [47] vs. 2.45 μm (myelinated) & 0.74 μm (unmyelinated) [79]). Notably, the prominent second peak in the myelinated CNAPs that used Soultanpour & Santer [79] data resembled the CNAPs from the undersampled distributions in Fig 9C, suggesting a potential undersampling of the histogram. The limited data on inter-individual nerve fiber histograms prevents evaluating the role of inter-individual differences of fiber diameter distributions in accounting for inter-individual differences in CNAPs, and this is an important limitation for model validation. Our findings motivate the need for additional measurements of individual-specific fiber diameter distributions and nerve anatomy to develop more accurate CNAP predictions.

#### 6.3.2 CV vs. Fiber diameter

The assumed relationship between CV and fiber diameter can dictate the amplitude, shape, and latency of modeled CNAPs. While CV values in biophysical models of nerve fibers emerged from the physical properties defined for the fibers (S17 Text), these CV values differed from in vivo measurements (S18 Text), and this discrepancy led to quite distinct modeled CNAPs (Fig 11). While altering the CV-to-fiber diameter relationship involves overriding the values calculated by the biophysical models, our modeling framework allows the exploration of such relationships. Interestingly, the linear fit of Hoffmeister [80] produced a smaller amplitude unmyelinated fiber signal but had only a limited effect on latency, suggesting a role of CV-to-fiber diameter relationship (i.e., linear with fiber diameter vs. linear with the square root of fiber diameter). We only used experimental measurements from two studies as the source of CV measurements, and given the observed potential importance of appropriate CV values on CNAP signals, future use of more sources is warranted.

#### 6.3.3 Conduction distance

Our findings show that there is a tradeoff between shortening conduction distances to increase CNAP amplitude and lengthening conduction distances to decrease stimulation artifact overlap with CNAPs. The decrease in CNAP amplitude with increasing conduction distance is caused by temporal dispersion of action potentials [22,25,26,29,30,40]. We showed that this decrease approximately follows an inverse square relationship within the feasible range of conduction distances in the rat cervical vagus nerve of <20 mm. The implication of these findings for unmyelinated fibers, for which the stimulus artifact is much shorter than the expected latencies, is that minimizing the conduction distance can maximize the amplitude of CNAPs without overlapping with the stimulation artifact. On the other hand, myelinated fiber signals are likely best recorded at intermediate conduction distances that reduce attenuation due to dispersion (<10 mm) and are still far enough to avoid overlap with the stimulus artifact.

At a conduction distance of 11 mm, CNAPs for unmyelinated fibers were ~3-5x larger than for myelinated fibers because there were approximately 10x more unmyelinated fibers; this larger fiber count offset their ~2-3x lower SFAP amplitude. Consistent with our CV vs. fiber diameter analysis (Fig 11), temporal dispersion increases as the CV becomes slower and as the standard deviation of CV increases [24]. However, unmyelinated fibers exhibited only slightly stronger temporal dispersion than myelinated fibers (Fig 8) despite having much slower CVs. This occurred because SFAP duration was much longer for unmyelinated fibers compared to myelinated fibers (S6 Text); the longer SFAP duration compensated for the slower signals, resulting in comparable degrees of dispersion in both fiber types (S20 Text). This finding is consistent with our in vivo data, indicating a comparable degree of temporal dispersion between myelinated and unmyelinated signal (Fig 13 and S20 Text).

#### 6.3.4 Fiber XY Position and Z Jitter

Fiber location within the nerve cross section had a limited effect on CNAP characteristics. Three factors likely account for this result: (1) the circularly symmetric electrode geometry paired with a circularly symmetric monofascicular nerve geometry can distinguish between signals from nerve fibers at different radial locations but not angular locations; (2) CNAP signals are aggregates of a population of SFAPs such that differences due to location get averaged out; and (3) the presence of a resistive perineurium makes potentials from each fiber in the fascicle appear more uniform. The effect of perineurium can be understood by considering the reciprocal relationship between stimulation and recording: stimulating nerve fibers within a fascicle requires higher amplitudes when perineurium is present, and threshold amplitudes are nearly uniform for a given fiber diameter anywhere within the fascicle [87,88]. Therefore, it follows from the electromagnetic reciprocity theorem that signals originating from across the fascicle for a given fiber diameter will be detected as similar amplitudes at the recording electrode. The limited effect of fiber location and longitudinal jitter challenge the goal of selective recording of specific fiber populations in the case of a monofascicular nerve. However, the use of high density electrodes to obtain spatiotemporal profiles [42] may provide sufficient information to decode spatial-specific information.

### 6.4 Model vs. In Vivo comparison

Our initial comparison of modeled vs. in vivo CNAPs showed a large discrepancy in amplitudes (Fig 1), while tuning models within experimentally feasible parameter values based on volume conductor sensitivity analysis generated amplitudes comparable to in vivo CNAPs for myelinated fibers (Fig 13). Previous studies recording from large myelinated fibers reported a strong correspondence between model predictions and in vivo recordings in terms of shape and latency, although those studies most often did not consider signal amplitude, and they used triangular SFAP shapes with CV tuning to achieve a strong match [22,25,27,30]. Two studies reported comparable amplitudes of biophysical model predictions to in vivo data for myelinated and unmyelinated fibers (3x or less discrepancy) [39,41]. However, comparing amplitude and shape for myelinated fibers was confounded by a strong stimulation artifact overlapping with the fastest signal components due to the very short conduction distance (2.85 mm) [41]. As a result, neither the shape nor latency of the CNAPs resembled the modeled signals, and the amplitude discrepancy was larger depending on the in vivo trial. Meanwhile, Lubba and colleagues recorded CNAPs at a very large conduction distance (80 mm) [39] such that it was challenging to compare the shape and latency of the modeled CNAPs to the in vivo data since very temporally dispersed CNAPs resembled noisy time series. In contrast to Eiber and colleagues’ use of nerve-specific fiber diameters, it was unclear how Lubba and colleagues selected the number and diameters of myelinated and unmyelinated fibers, and this selection can impact amplitude substantially. Finally, Lubba and colleagues shifted the unmyelinated fiber SFAPs in time, using CV [in meter/sec] = 1.4*sqrt(fiber diameter [in micrometers], which in turn can also influence amplitudes. In our study, our use of intermediate conduction distances in vivo (6–15 mm) enabled us to compare our modeled CNAPs to in vivo CNAPs in terms of shape, latency, and amplitude and revealed the discrepancies and their potential sources. We also extended previous comparisons by conducting measurements across conduction distances and channels. The modeled myelinated fiber CNAPs had comparable shapes to in vivo CNAPs. Recording channel effects were qualitatively consistent between the model and in vivo signals, showing a decrease in amplitude and increase in latency from channels 1 to 3.

Model amplitudes could be made comparable to in vivo amplitudes by adjusting tissue conductivity and cuff fit on the nerve, but could not be made comparable to in vivo signals for both myelinated and unmyelinated fibers simultaneously. Further, the latency of CNAPs in the models was slower than in vivo. Several factors may contribute to these discrepancies. The frequency content of unmyelinated fiber CNAPs overlaps substantially with the high pass filter used to reject powerline noise (S21 and S22 Text files), which may contribute to the amplitude discrepancies. Uncertainty in the conduction distance may partially account for the latency or amplitude discrepancies between in vivo and model data. However, since the effects of conduction distance and volume conductor parameters are expected to affect myelinated and unmyelinated fibers similarly, these factors alone do not account for in vivo vs. model discrepancies of both fiber types. The two fiber types are based on two distinct fiber models and it is not surprising that they differ in their ability to match the in vivo data. Available models of unmyelinated fibers, including the model that we used [46], produce action potentials with CV and strength-duration responses consistent with experimental measurements, but other characteristics such as recovery cycle and action potential shape do not match well with experimental measurements [89]. There is a need for models of unmyelinated fibers that reproduce experimental measurements, including in vivo CNAP recordings. Our framework for modeling CNAPs can readily incorporate updated biophysical models of unmyelinated fibers.

### 6.5 Limitations

The latency of each action potential template includes action potential initiation and action potential propagation, and the former varies with the type of stimulus pulse, the stimulus amplitude, and the electrode geometry. Therefore, the action potential templates are most accurate for predicting CNAPs evoked by the specific stimulation model that produced them. Initiation delays would result in subtle sub-millisecond changes to CNAPs, although these are expected to become less relevant as the conduction distance increases. Action potential templates should thus be accompanied by metadata that would enable evaluating subtle effects (e.g., stimulation electrode geometry, stimulation pulse shape, location and time from which the template was taken, etc.).

We maintained the recording cuff length at 3,650 μm across all simulations. Prior studies showed that recorded signals increased with cuff length, to a plateau: SFAP peak-to-peak amplitude plateaued after a certain cuff length in myelinated fibers [20,33,39] and was maximum at a certain cuff length in unmyelinated fibers [39]. Andreasen proposed a quantitative relationship between the optimal cuff length and the conduction velocity, internodal distance, and the action potential duration of a given fiber diameter [33] and later provided a theory of active length that can guide the selection of cuff length [90]. Other parameters that remain to be evaluated systematically for effects on CNAPs include temperature [26, 27], intracellular conductivity [25], activity-dependent slowing in unmyelinated fibers [46], and ephaptic coupling.

Our simplified rat cervical vagus nerve model included a range of parameters but did not include scar tissue, shrinkage of the nerve (i.e., as opposed to shrinkage of the nerve fibers), cuff rotation, and variation of perineurium thickness. Further, with larger nerves found in pigs and humans, additional complexities emerge due to nerve geometry, additional conductivity parameters, and inter-individual variability (e.g., size, number, and locations of fascicles). Such parameters can be included in volume conductor models but were beyond the scope of the present work. We also only conducted the study on monopolar recordings, which are referenced to a distant ground; bipolar or tripolar recordings use distinct references that could alter the effects of tissue conductivities, conduction distance, and nerve fiber parameters on recorded signals.

We evaluated models assuming that the axons were straight. However, the presence of non-straight axons may have a large effect on the signal recorded from an axon [39]. Non-straight axon trajectories only change the specific values of recording sensitivity function in Eq. 5, which is obtained by sampling the volume conductor model at coordinates along the axon trajectory; the transmembrane current matrix and the shift matrix in Eq. 5 remain unchanged.

We compared modeled CNAPs in response to activation of all fibers in the nerve to in vivo CNAPs in response to a stimulation amplitude that evoked maximal CNAP responses. We did not evaluate the accuracy of the modeled CNAPs when subsets of the nerve were active (e.g., graded activation), although representation of subpopulations of active axons can be readily incorporated in the model.

### 6.6 Conclusion

In conclusion, we developed a method to model CNAPs efficiently in whole-nerve models, we applied the method to quantify the sensitivity of CNAPs to tissue and recording parameters, and we compared modeled CNAPs to in vivo data. The method calculated CNAPs with 27,000–1,900,000x speedup compared to brute force while maintaining accuracy for both myelinated and unmyelinated fibers. Tissue conductivity had a large effect on the amplitude of CNAPs, whereas the distribution of fiber diameters and the CV-to-fiber diameter relationship had a large effect on the shape and latency. The shape, latency, and amplitude of modeled CNAPs corresponded well to in vivo recordings for myelinated fibers, while discrepancies in amplitude indicated a need for unmyelinated fiber models that match experimental measurements. Our framework for CNAP modeling is publicly available to facilitate physiological insights and the design of neural recording interfaces for closed-loop therapies.

## Supporting information

S1 Text Effect of Fiber Location Within Nerve. (DOCX)

S2 Text Myelinated Fiber Ultrastructure Fits. (DOCX)

S3 Text Ion Channel Max Conductance Tuning. (DOCX)

S4 Text Transmembrane Potential Across Myelinated Fiber Diameters. (DOCX)

S5 Text Net Extracellular Current. (DOCX)

S6 Text Filtered Interpolated Action Potential Templates Matched Brute Force SFAPs at Arbitrary Fiber Diameters. (DOCX)

S7 Text Temporal Template Interpolation Revealed Necessary Precision for Accurate CNAP Reconstruction. (DOCX)

S8 Text Monopolar and Dipolar Representations. (DOCX)

S9 Text Minimum Number of Templates Depended Strongly on Conduction Distance and Interpolation. (DOCX)

S10 Text Tissue Conductivities Effects on Myelinated Fiber CNAPs (full). (DOCX)

S11 Text Tissue Conductivities Effects on Unmyelinated Fiber CNAPs (partial). (DOCX)

S12 Text Tissue Conductivities Effects on Unmyelinated Fiber CNAPs (full). (DOCX)

S13 Text Tissue Conductivities Effects on Myelinated Fiber CNAPs (full, with cuff opening). (DOCX)

S14 Text Tissue Conductivities Effects on Unmyelinated Fiber CNAPs (full, with cuff opening). (DOCX)

S15 Text Tissue Conductivities Effects on Unmyelinated Fiber CNAPs Waveform shape. (DOCX)

S16 Text Effect of Random Sampling in Unmyelinated Fibers. (DOCX)

S17 Text CV vs. Fiber Diameter Relationship in Models. (DOCX)

S18 Text CV vs. Fiber Diameter Relationship in Literature. (DOCX)

S19 Text Effect of Longitudinal Jitter. (DOCX)

S20 Text Temporal Dispersion In Vivo vs. Model. (DOCX)

S21 Text Frequency Content of Myelinated and Unmyelinated Fibers. (DOCX)

S22 Text Analog Filtering. (DOCX)

S23 Text No Perineurium. (DOCX)
