# Supplementary material for: Computational models of compound nerve action potentials: Efficient filter-based methods to quantify effects of tissue conductivities, conduction distance, and nerve fiber parameters
Source: PLoS Comput Biol. 2024 Mar 1;20(3):e1011833. doi: 10.1371/journal.pcbi.1011833 (PMC10936855; doi:10.1371/journal.pcbi.1011833)
Supplement: S1 Text — (DOCX) [file pcbi.1011833.s001.docx]

S1 Text: Effect of Fiber Location Within Nerve

*
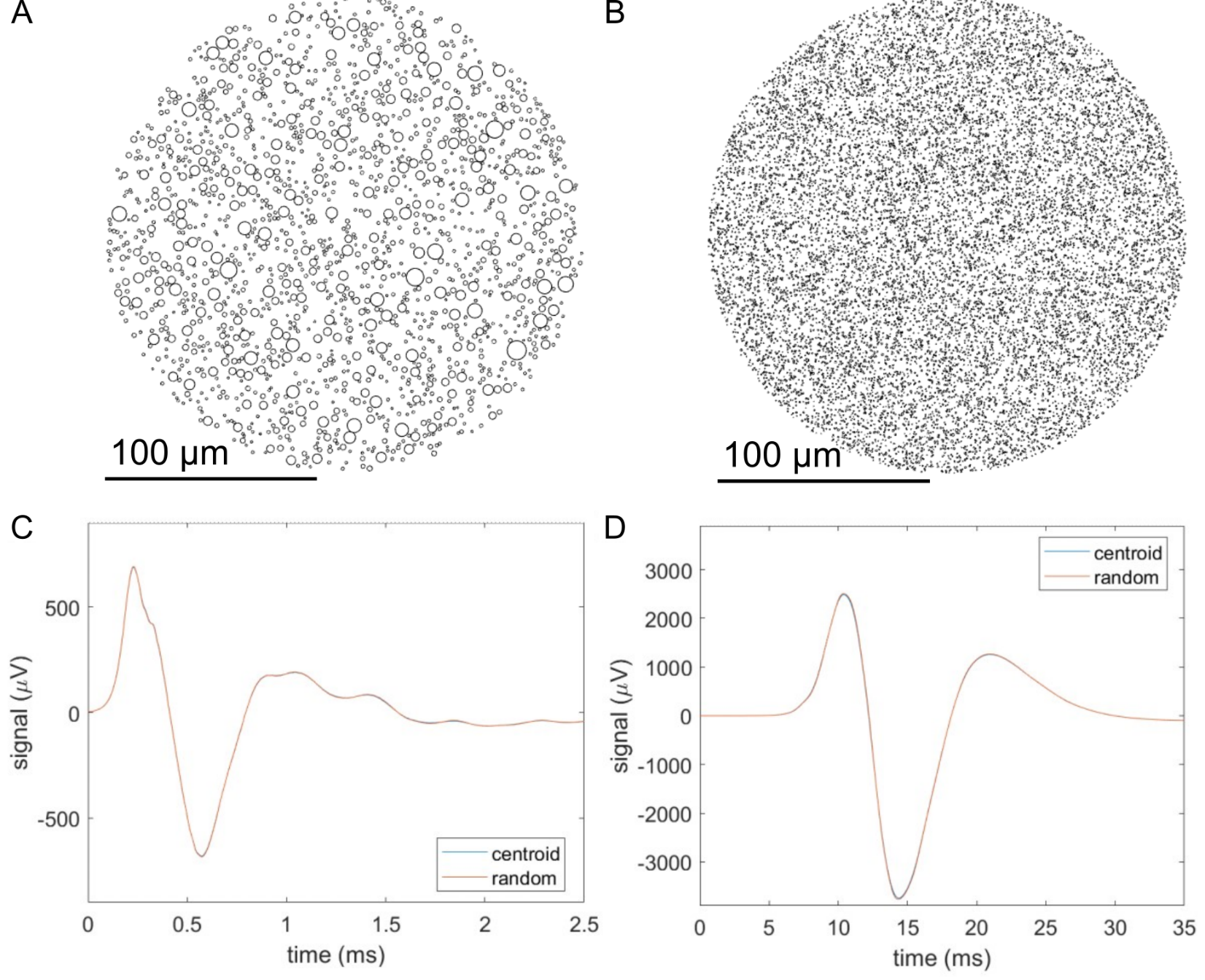
*

*Figure A. Effect of fiber location of myelinated (A) and unmyelinated (B) fibers on CNAPs from myelinated (C) and unmyelinated fibers (D). Placing fibers randomly across the rat cervical vagus nerve fascicle (‘random’; A-B) produced CNAPs that were nearly identical to those obtained from placing all fibers at the centroid of the fascicle (‘centroid’).*
