# Supplementary material for: Computational models of compound nerve action potentials: Efficient filter-based methods to quantify effects of tissue conductivities, conduction distance, and nerve fiber parameters
Source: PLoS Comput Biol. 2024 Mar 1;20(3):e1011833. doi: 10.1371/journal.pcbi.1011833 (PMC10936855; doi:10.1371/journal.pcbi.1011833)
Supplement: S2 Text — (DOCX) [file pcbi.1011833.s002.docx]

S2 Text: Myelinated Fiber Ultrastructure Fits

We performed all linear or quadratic fits in MATLAB R2018a (‘fit’ function with ‘poly1’ or ‘poly2’ option). We subtracted the mean of the independent variables from each independent variable data point to ensure fit stability and avoid multicollinearity as well as to produce readily interpretable fit parameters. We weighted or transformed the dependent or independent variable to ensure nonnegative parameter values with means and uncertainties that tracked the data from literature. Specifically, for FLUT length and internodal length, we performed weighted linear least squares regression (via the ‘Weight’ option of the ‘fit’ function) with weights equal to the inverse of the square of the FLUT length or internodal length, respectively, due to the observation that data uncertainty was proportional to the parameter value itself. For internodal axon diameter and nodal axon diameter, we performed linear least squares regression on the g-ratio (i.e., ratio of internodal axon diameter to myelin diameter) and on the ratio of the nodal axon diameter to the internodal axon diameter. For the number of myelin lamellae, we performed linear least squares regression on the natural log of the number of lamellae. The transformations and weighting are reflected in the final fit equations shown in Figure A.


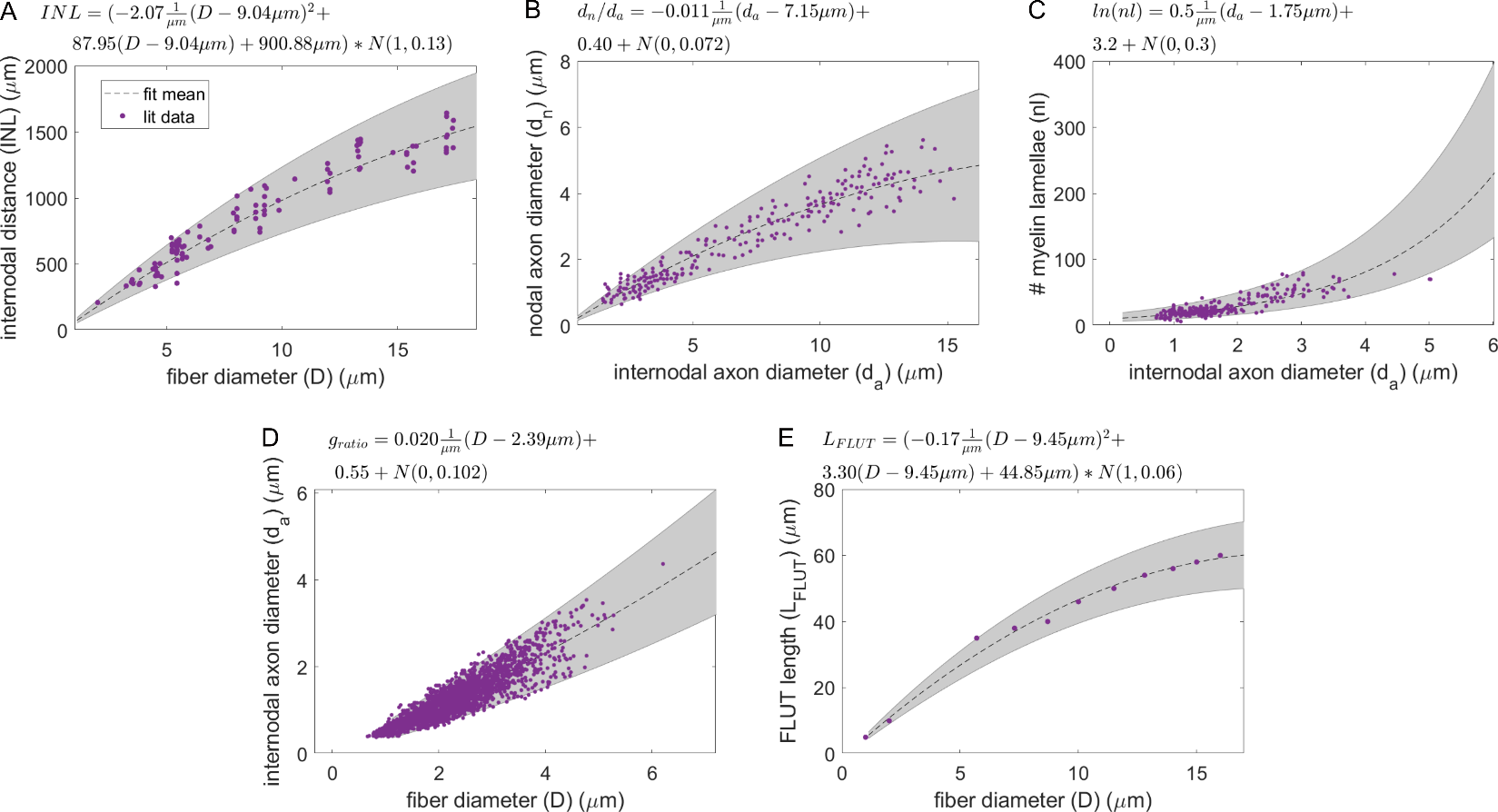


*Figure A. Models of ultrastructure parameters for myelinated fibers (model equations denoted above each panel; Gaussian noise specified as N(mean, std)). Models were constructed via linear regression of ultrastructure parameters from the literature (yellow dots) to quantify the mean and variance of each parameter (black dotted line and gray shaded area). The gray areas represent the 95% confidence interval of the data variability. (A) Internodal length (INL) vs. fiber diameter (D) (fit to data from adult cat shown in Figure 3 of* (1)*), with uncertainty modeled as a fraction of internodal length. The STIN length is calculated as the internodal length (panel A), minus the nodal length (fixed at 1 µm), minus twice the MYSA length (fixed at 3 µm), minus twice the FLUT length (panel E). (B) Ratio of nodal axon diameter (d_n_) to internodal axon diameter (d_a_) (fit to data from Figures 3 & 4 of* (2)*). The nodal axon diameter is also equal to the MYSA axon diameter. (C) Number of myelin lamellae (nl) (fit to data from Figures 3A to 3C of* (3)*), with data log-transformed to exploit the discrete nature of the parameter via assumption of a Poisson distribution of noise. (D) Internodal axon diameter (fit to data from Figure 3 of* (3) *and Figure 3 (proximal and distal) from* (4)*), which was transformed to g-ratio (ratio of internodal axon diameter to myelin diameter) for fitting. This parameter is also equal to the FLUT axon diameter. (E) FLUT length (L_FLUT_) (fit to data from* (5,6)*), with uncertainty modeled as a fraction of FLUT length.*

References

1. Hursh JB. Conduction Velocity and Diameter of Nerve Fibers. American Journal of Physiology-Legacy Content. 1939 Jul 31;127(1):131–9.

2. Rydmark M. Nodal axon diameter correlates linearly with internodal axon diameter in spinal roots of the cat. Neuroscience Letters. 1981 Jul;24(3):247–50.

3. Friede RL, Samorajski T. Relation between the number of myelin lamellae and axon circumference in fibers of vagus and sciatic nerves of mice. J Comp Neurol. 1967 Jul;130(3):223–31.

4. Fazan VPS, Salgado HC, Barreira AA. A Descriptive and Quantitative Light and Electron Microscopy Study of the Aortic Depressor Nerve in Normotensive Rats. Hypertension. 1997 Sep;30(3):693–8.

5. McIntyre CC, Richardson AG, Grill WM. Modeling the Excitability of Mammalian Nerve Fibers: Influence of Afterpotentials on the Recovery Cycle. Journal of Neurophysiology. 2002 Feb;87(2):995–1006.

6. McIntyre CC, Grill WM, Sherman DL, Thakor NV. Cellular Effects of Deep Brain Stimulation: Model-Based Analysis of Activation and Inhibition. Journal of Neurophysiology. 2004 Apr;91(4):1457–69.
