# Supplementary material for: Computational models of compound nerve action potentials: Efficient filter-based methods to quantify effects of tissue conductivities, conduction distance, and nerve fiber parameters
Source: PLoS Comput Biol. 2024 Mar 1;20(3):e1011833. doi: 10.1371/journal.pcbi.1011833 (PMC10936855; doi:10.1371/journal.pcbi.1011833)
Supplement: S3 Text — (DOCX) [file pcbi.1011833.s003.docx]

S3 Text: Ion Channel Max Conductance Tuning

The specific combination of maximum sodium and potassium channel conductance affected firing responses in the fibers with ion channel parameters from (63). With the default combination of maximum conductance values (i.e., gnabar=3 S/cm^2 & gkbar=0.8 S/cm^2), multiple irregular action potentials occurred at some small fiber diameters (Figure A). Scaling gnabar and gkbar independently by 0 .33, 0.56, 0.78, 1.00, 1.22, 1.44, 1.67 and simulating all possible conductance combinations revealed that increasing the gnabar or decreasing gkbar could further increase firing or produce other unusual transmembrane potentials (Figure A). In contrast, decreasing the fast sodium channel’s maximum conductance or increasing the potassium channel’s maximum conductance could decrease multiple action potential firing. For all our analyses in the main text, we set gnabar to 2.333 S/cm^2 and gkbar to 0.116 S/cm^2 because the transmembrane potentials at those maximum conductances exhibited only a single action potential across all simulated axons (S4 Text).


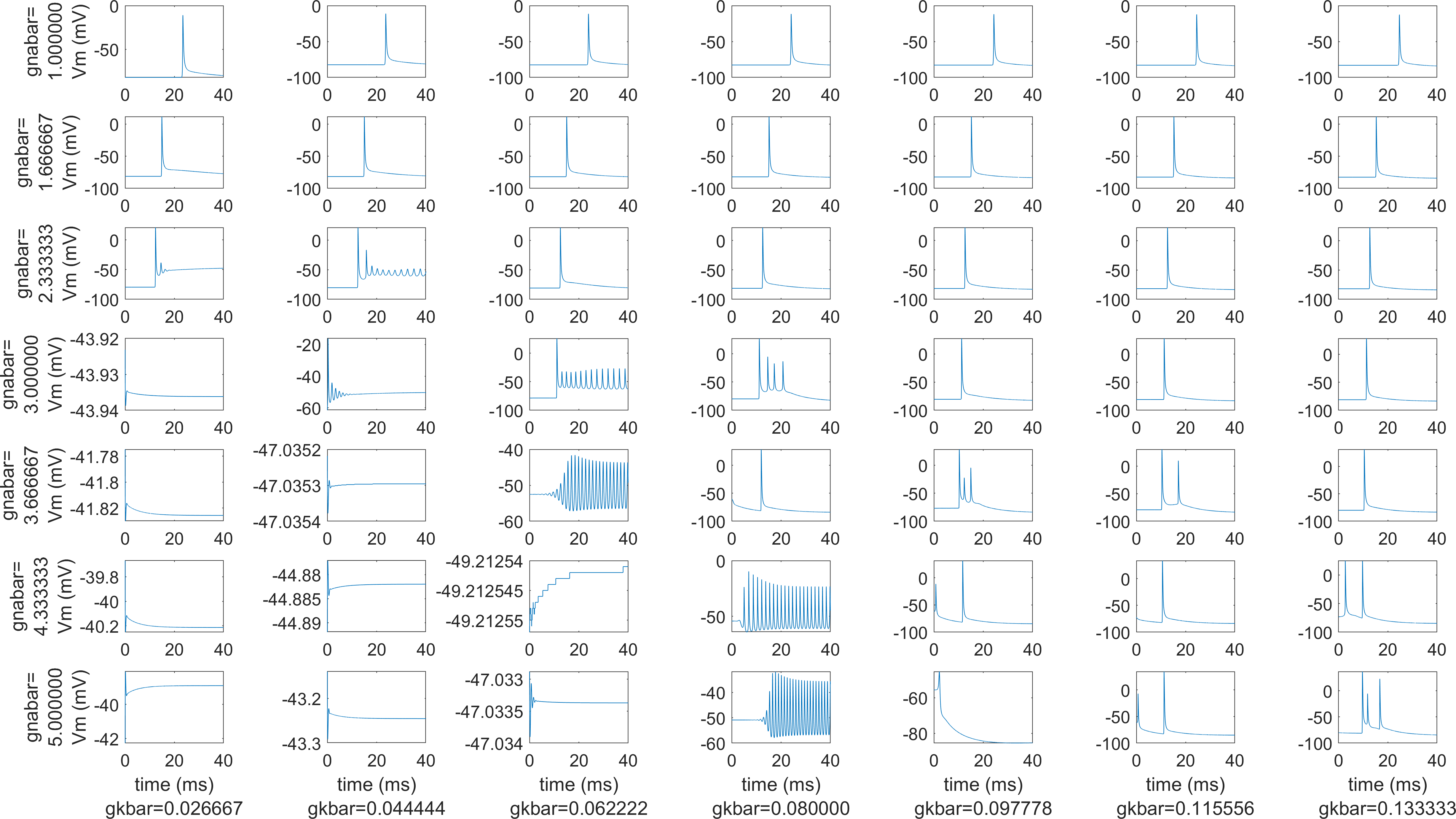


*Figure A. Transmembrane potential traces at a single node of a 50 mm-long, 1.6 μm diameter myelinated fiber across a range of fast sodium channel maximum conductances (gnabar, in S/cm^2; row labels) and a range of maximum potassium channel conductance values (gkbar, in S/cm^2; column labels). The default conductances from (63) are gnabar=3 S/cm^2 and gkbar 0.8 S/cm^2 (center panel). We stimulated each axon with an intracellular stimulus pulse of 0.8 nA amplitude at the second node of Ranvier, and we recorded the transmembrane potential at the node closest to the 40 mm point along each axon.*
