# Supplementary material for: Computational models of compound nerve action potentials: Efficient filter-based methods to quantify effects of tissue conductivities, conduction distance, and nerve fiber parameters
Source: PLoS Comput Biol. 2024 Mar 1;20(3):e1011833. doi: 10.1371/journal.pcbi.1011833 (PMC10936855; doi:10.1371/journal.pcbi.1011833)
Supplement: S4 Text — (DOCX) [file pcbi.1011833.s004.docx]

S4 Text: Transmembrane Potential Across Myelinated Fiber Diameters

**
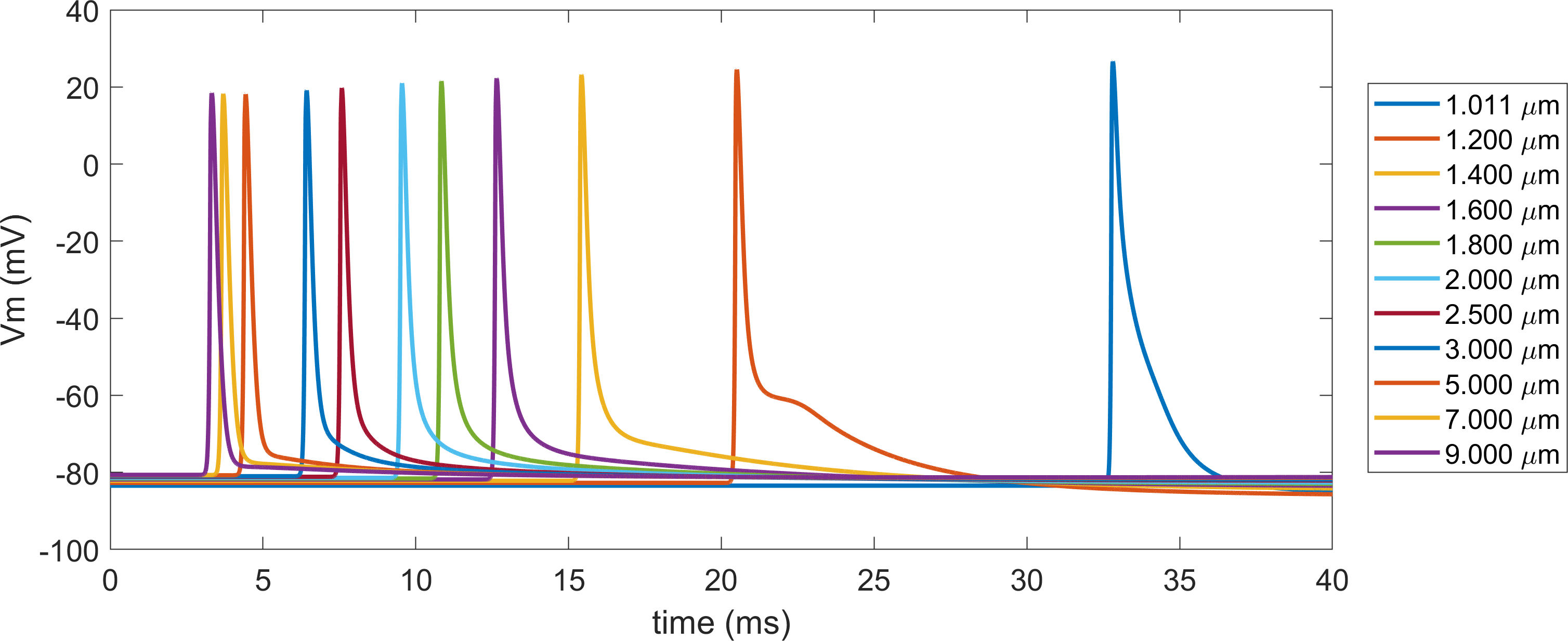
**

*Figure A. Action potentials across myelinated fiber diameters with gnabar=2.333 S/cm^2 and gkbar=0.116 S/cm^2. The line colors are not unique across fiber diameters, but the latencies are ordered from fastest fiber (9 μm) to the slowest fiber (1.011 μm). We stimulated each axon with an intracellular stimulus pulse of 0.8 nA amplitude at the second node of Ranvier, and recorded the transmembrane potential at the node we closest to the 40 mm point along each axon.*
