# Supplementary material for: Computational models of compound nerve action potentials: Efficient filter-based methods to quantify effects of tissue conductivities, conduction distance, and nerve fiber parameters
Source: PLoS Comput Biol. 2024 Mar 1;20(3):e1011833. doi: 10.1371/journal.pcbi.1011833 (PMC10936855; doi:10.1371/journal.pcbi.1011833)
Supplement: S5 Text — (DOCX) [file pcbi.1011833.s005.docx]

S5 Text: Net Extracellular Current


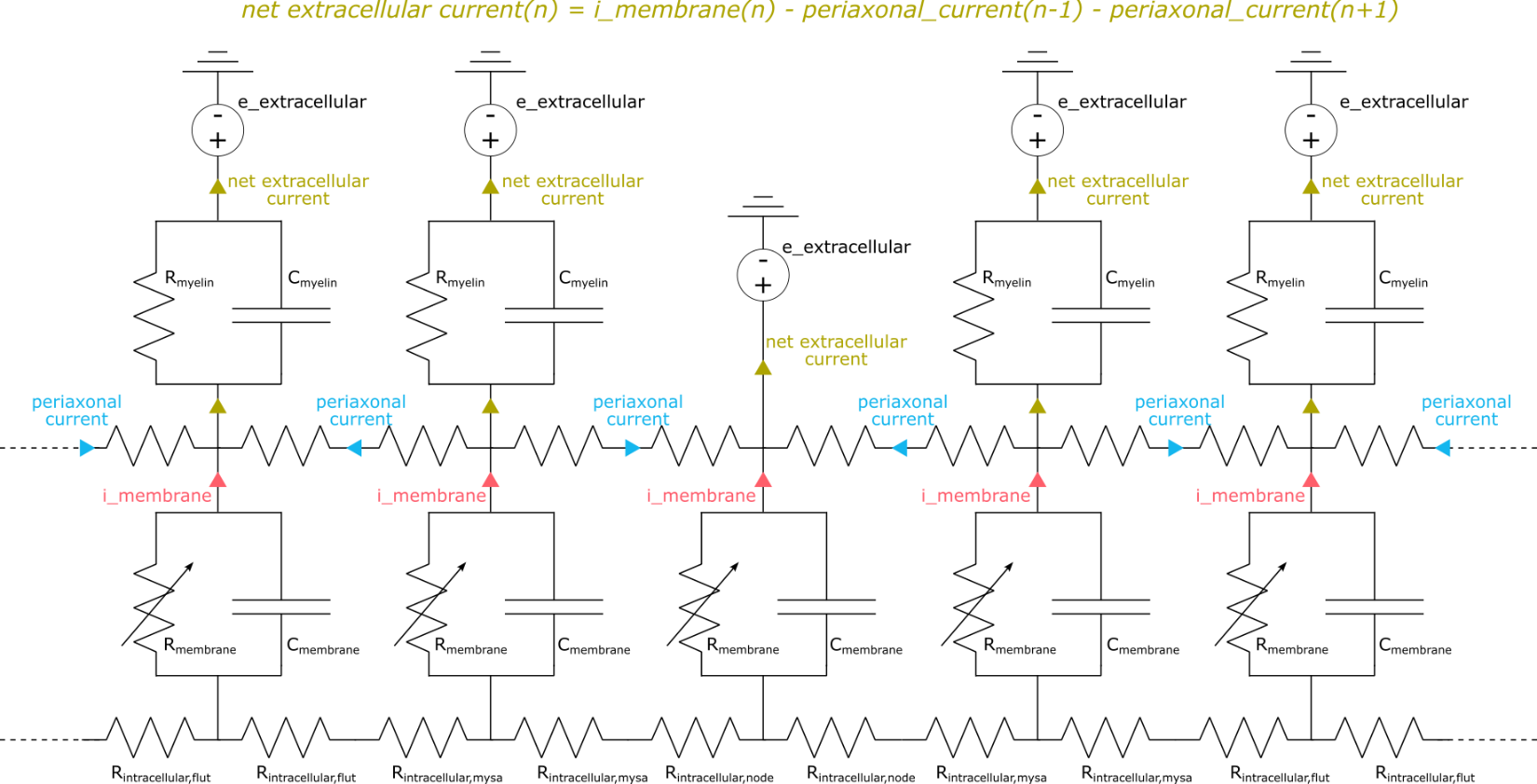


*Figure A. Illustration of net extracellular current calculation at compartments around a node of Ranvier in a myelinated fiber. For each compartment, the net extracellular current is given by Kirchoff’s current law: net extracellular current(n) = i_membrane(n) - periaxonal current(n-1) - periaxonal current(n+1). This calculation applies to every compartment type in myelinated and unmyelinated fibers, although in unmyelinated fibers the net extracellular current equals i_membrane since the periaxonal current is zero. To simplify visualization, the voltage-gated ion channels are lumped into a single R_membrane_, and the leak channel of the membrane is not shown.*
