# Supplementary material for: Computational models of compound nerve action potentials: Efficient filter-based methods to quantify effects of tissue conductivities, conduction distance, and nerve fiber parameters
Source: PLoS Comput Biol. 2024 Mar 1;20(3):e1011833. doi: 10.1371/journal.pcbi.1011833 (PMC10936855; doi:10.1371/journal.pcbi.1011833)
Supplement: S6 Text — (DOCX) [file pcbi.1011833.s006.docx]

S6 Text: Filtered Interpolated Action Potential Templates Matched Brute Force SFAPs at Arbitrary Fiber Diameters

Underlying the strong match between brute force CNAPs and those obtained by filtering interpolated templates was a strong match observed at the level of individual SFAPs (Figure A(A-D)). The filtered templates and brute force SFAPs had identical shapes and peak-to-peak amplitudes at the smallest (Figure A(A,C)) and largest (Figure A(B,D)) fiber diameters present in the nerve.

Also underlying accurate CNAP reconstruction was the ability to interpolate templates across fiber diameters that we did not simulate in NEURON. This approach produced accurate SFAPs when we used a sufficiently large number of templates to sample the fiber diameter range (Figure A(E-H)). Using 97 myelinated fiber templates or 25 unmyelinated fiber templates produced a strong match between filter-derived SFAPs and brute force SFAPs, while coarser sampling of the fiber diameter range resulted in noticeable latency and amplitude discrepancies. The peak-to-peak amplitudes from filtered interpolated action potential templates matched those of the brute force SFAPs across all fiber diameters simulated (S7 Text).


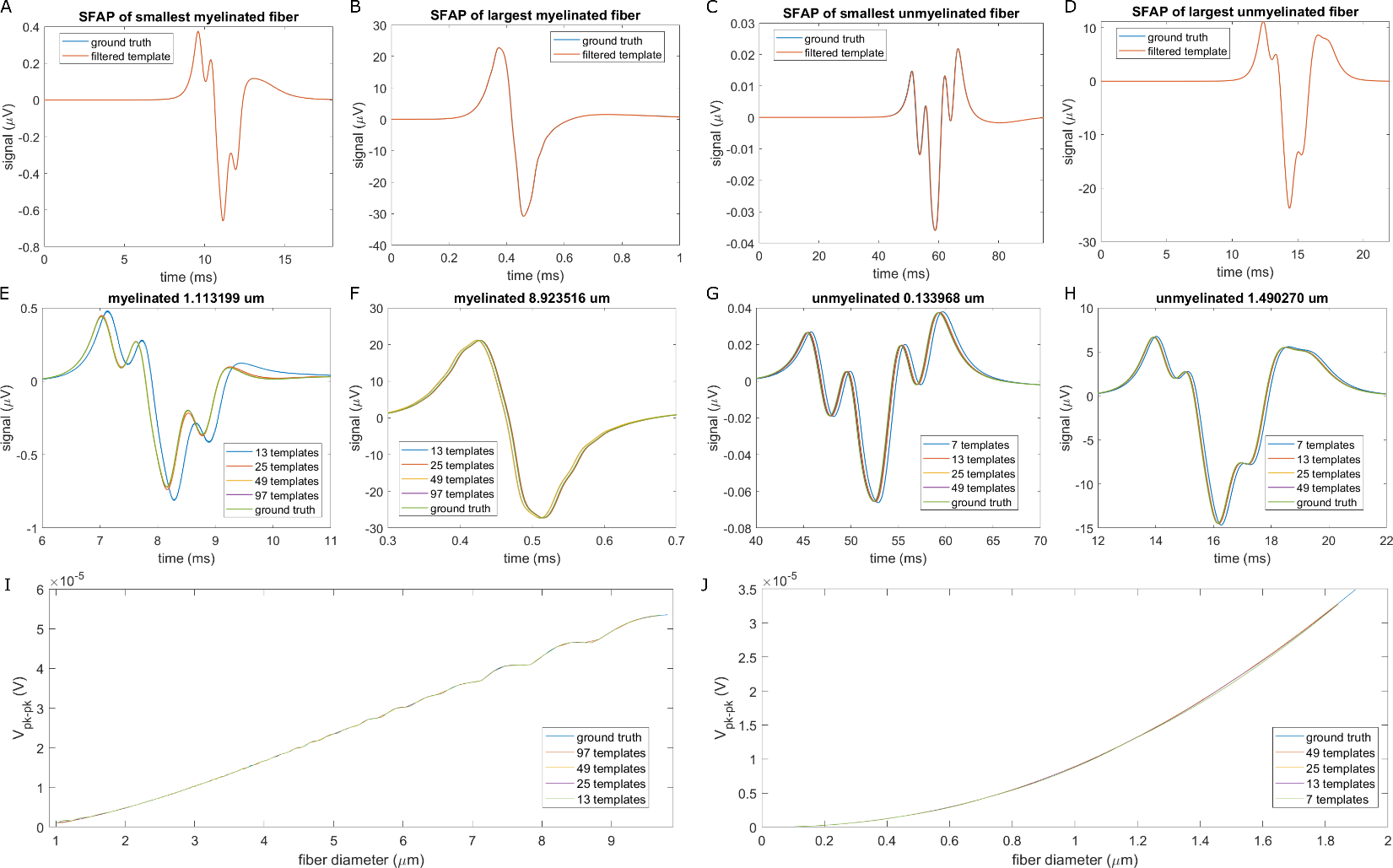


*Figure A. SFAPs calculated using the brute force method (ground truth) vs. signals produced via filtered action potential templates (filtered template). (A-D) Overlay of SFAPs across the smallest and largest myelinated and unmyelinated fibers in the rat cervical vagus nerve where the fiber diameters. (E-H) Overlay of SFAPs across the smallest and largest myelinated and unmyelinated fibers in the rat cervical vagus nerve. (I-J) Comparison of peak-to-peak SFAP amplitudes across all simulated fiber diameters for both myelinated (I) and unmyelinated (J) fibers.*
