# Supplementary material for: Computational models of compound nerve action potentials: Efficient filter-based methods to quantify effects of tissue conductivities, conduction distance, and nerve fiber parameters
Source: PLoS Comput Biol. 2024 Mar 1;20(3):e1011833. doi: 10.1371/journal.pcbi.1011833 (PMC10936855; doi:10.1371/journal.pcbi.1011833)
Supplement: S7 Text — (DOCX) [file pcbi.1011833.s007.docx]

S7 Text: Temporal Template Interpolation Revealed Necessary Precision for Accurate CNAP Reconstruction

The precision of fiber diameter measurements implicitly bins differently sized fibers to the chosen precision. We modeled CNAPs across a range of precision values to evaluate the coarsest precision necessary for accurate CNAP modeling. At coarse precisions and large conduction distances, CNAPs exhibited highly variable that were not present at finer precisions (Figure A(A)). These oscillations likely occurred due to synchrony effects similar to those observed for coarse fiber diameter binning (Figure A(A)). Precision 0.001 um achieved less than 10% deviation relative to the next finer precision across all conduction distances and in both myelinated and unmyelinated fiber, while precision of 0.01 um achieved required conduction distances ≤20 mm (myelinated) or ≤40 mm (unmyelinated) (Figure A(B)).


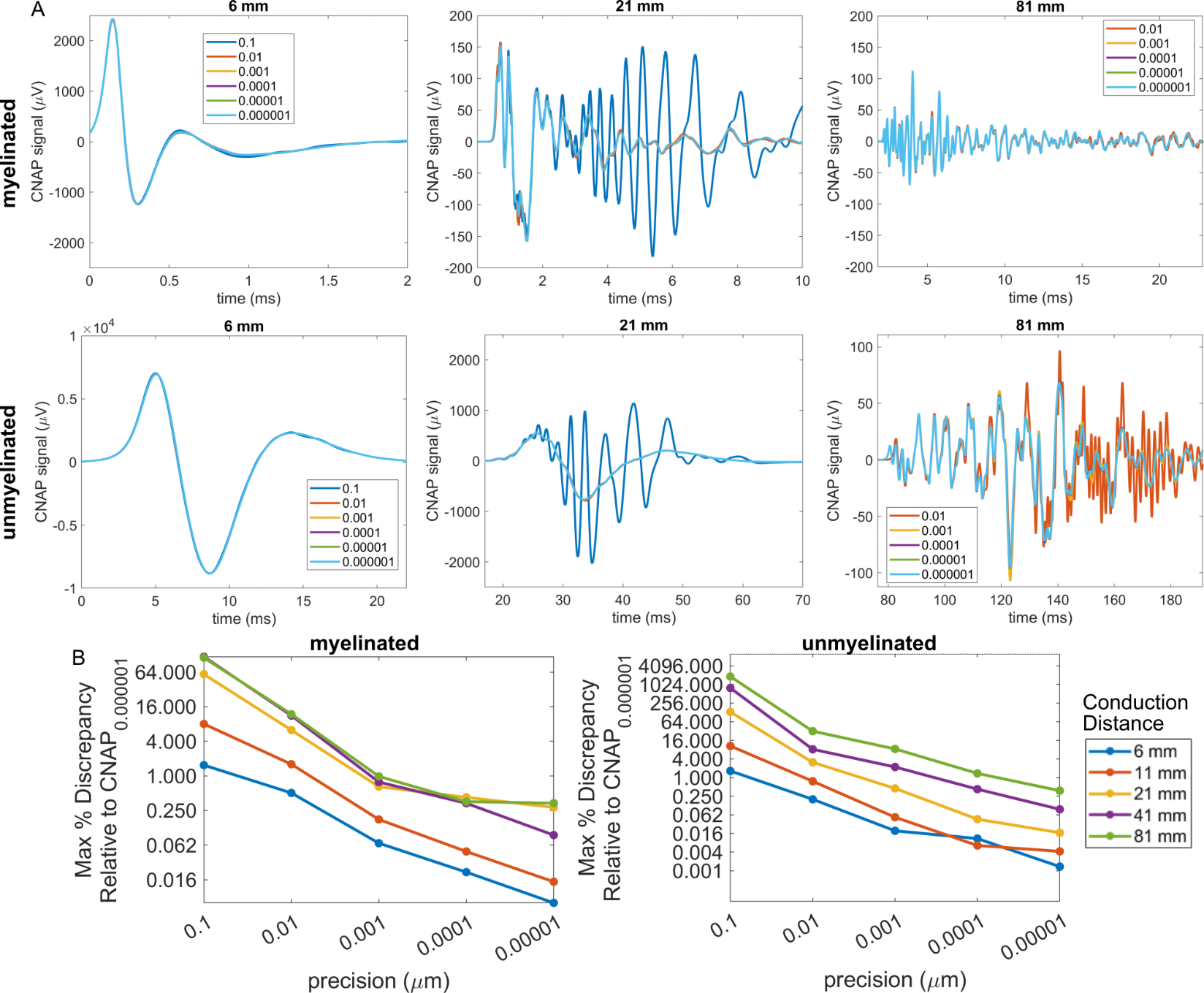


*Figure A. CNAPs constructed at different fiber diameter precisions and across five conduction distances. (A) Sample myelinated and unmyelinated CNAPs at 5, 20, and 80 mm. (B) CNAP amplitude compared to next finer resolution of action potential templates.*
