# Supplementary material for: Computational models of compound nerve action potentials: Efficient filter-based methods to quantify effects of tissue conductivities, conduction distance, and nerve fiber parameters
Source: PLoS Comput Biol. 2024 Mar 1;20(3):e1011833. doi: 10.1371/journal.pcbi.1011833 (PMC10936855; doi:10.1371/journal.pcbi.1011833)
Supplement: S8 Text — (DOCX) [file pcbi.1011833.s008.docx]

S8 Text: Monopolar and Dipolar Representations

| A  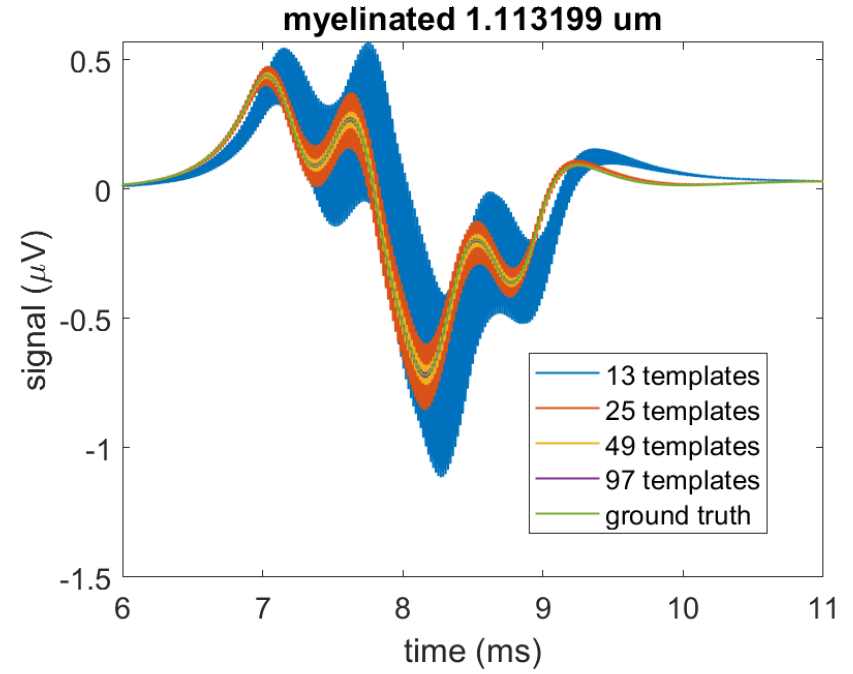 | B  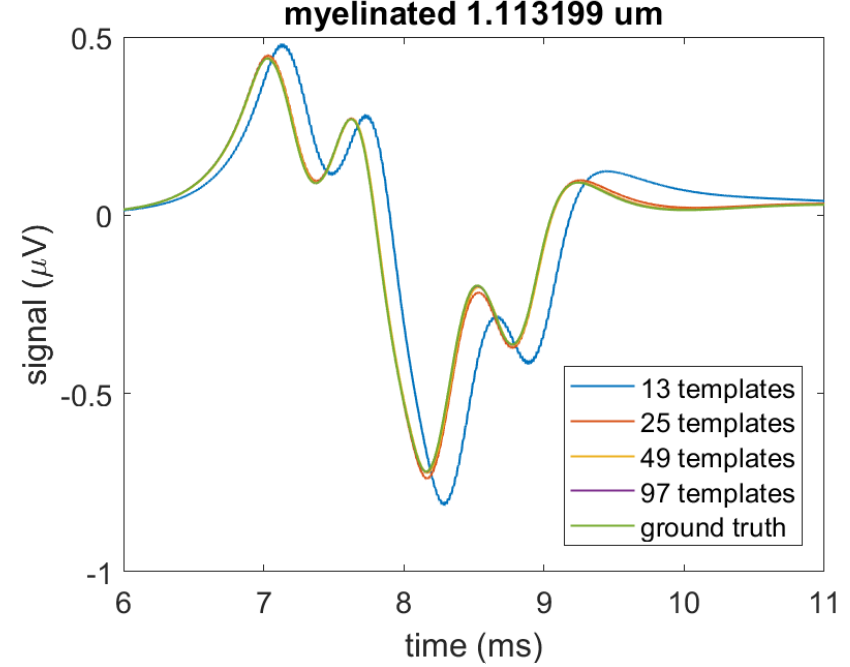 |
| --- | --- |

*Figure A. Effect monopolar (A) vs. dipolar (B) transmembrane current representation on the accuracy of myelinated fiber SFAP reconstruction in a small fiber diameter that was not part of the template diameters. Oscillations occurred for the monopolar representation that were not present for the dipolar representation.*
