# Supplementary material for: Computational models of compound nerve action potentials: Efficient filter-based methods to quantify effects of tissue conductivities, conduction distance, and nerve fiber parameters
Source: PLoS Comput Biol. 2024 Mar 1;20(3):e1011833. doi: 10.1371/journal.pcbi.1011833 (PMC10936855; doi:10.1371/journal.pcbi.1011833)
Supplement: S9 Text — (DOCX) [file pcbi.1011833.s009.docx]

S9 Text: Minimum Number of Templates Depended Strongly on Conduction Distance and Interpolation

We compared our use of linear interpolation across fiber diameters to a previous approach that did not use interpolation but instead grouped fiber diameters into a number of bins, equal to the number of templates (37); thus, all fiber diameters in a given bin were modeled using the template diameter. This binning approach reduced CNAP accuracy, and the accuracy was strongly dependent on the number of templates. At 6 mm conduction distance, at least 25 templates were needed for the binning approach vs. 7 templates for the linear interpolation approach to produce accurate CNAP shapes (A,D vs. Figure A(A,D)). At 81 mm in myelinated fibers, 193 myelinated fiber templates were needed for the binning approach vs. 7 templates for the linear interpolation approach (Figure A(C)), and no number of tested unmyelinated fiber templates was accurate for the binning approach. Inaccuracies in binning approach appeared as CNAPs with large oscillations due to excessive synchronization between fibers in the same bin (Figure A(B,C,E,F)).


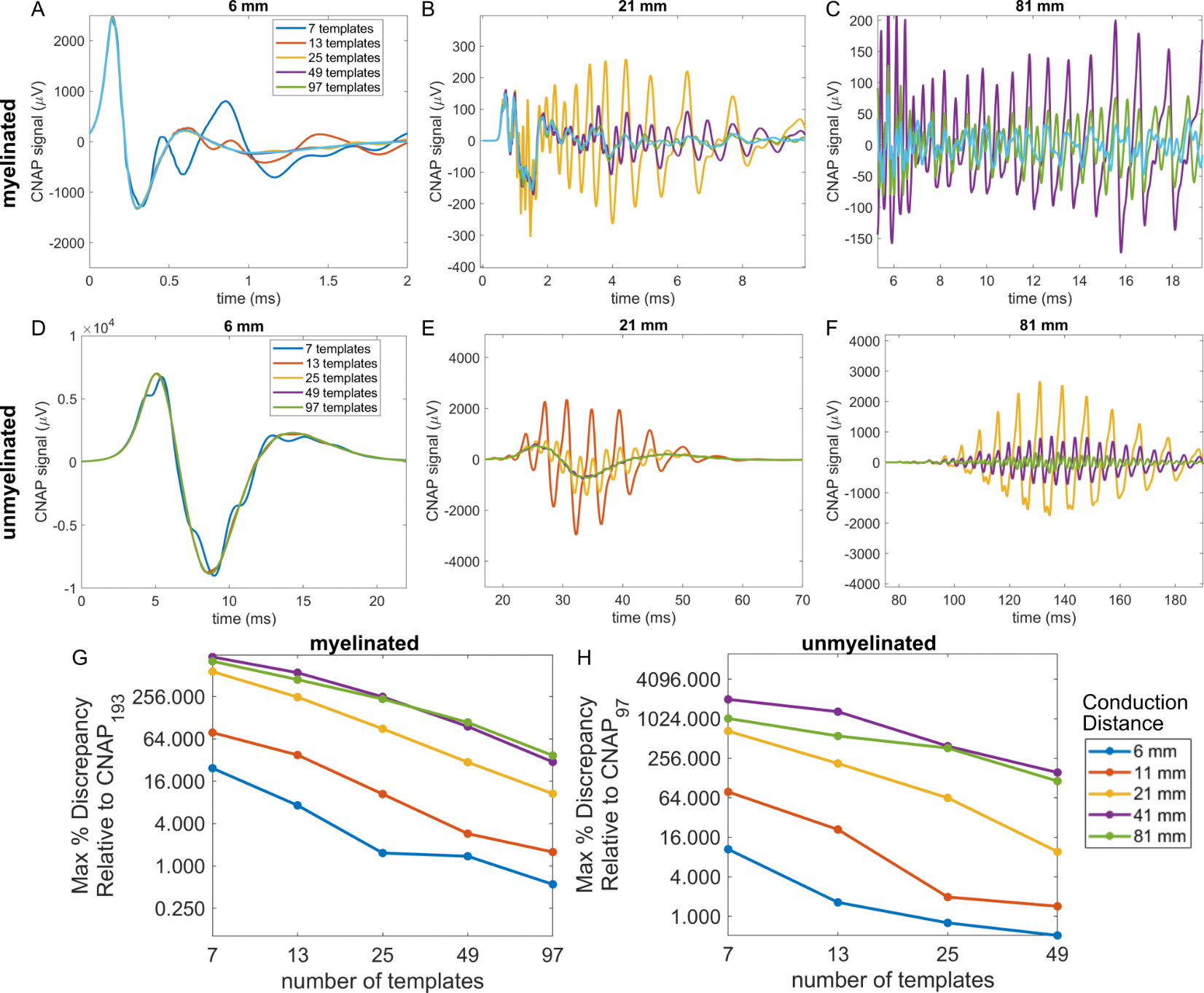


*Figure A. CNAPs modeled using the binning approach with different numbers of templates across fiber diameters (1.013 to 9.809 μm for myelinated fibers and 0.105 to 1.896 μm for unmyelinated fibers) at five conduction distances. In the binning approach, the number of bins was equal to the templates. For example, with 25 templates, myelinated fibers from 1.07 to 1.17 μm would all be represented by 1.11 μm myelinated fibers. (A-F) Example myelinated and unmyelinated CNAPs at conduction distances of 6, 21, and 81 mm. (G-H) Maximum deviation between CNAP signals using different numbers of templates compared to using the largest number of templates (normalized relative to the peak-to-peak amplitude of CNAP signals at the largest number of templates). For visual clarity and to maintain a reasonable zoom window, panels B, C, D, and F only show a subset of the comparisons because the discrepancies from using fewer templates resulted in signals with even larger amplitudes.*
