# Supplementary material for: Computational models of compound nerve action potentials: Efficient filter-based methods to quantify effects of tissue conductivities, conduction distance, and nerve fiber parameters
Source: PLoS Comput Biol. 2024 Mar 1;20(3):e1011833. doi: 10.1371/journal.pcbi.1011833 (PMC10936855; doi:10.1371/journal.pcbi.1011833)
Supplement: S11 Text — (DOCX) [file pcbi.1011833.s011.docx]

S11 Text: Tissue Conductivities Effects on Unmyelinated Fiber CNAPs (partial)

*
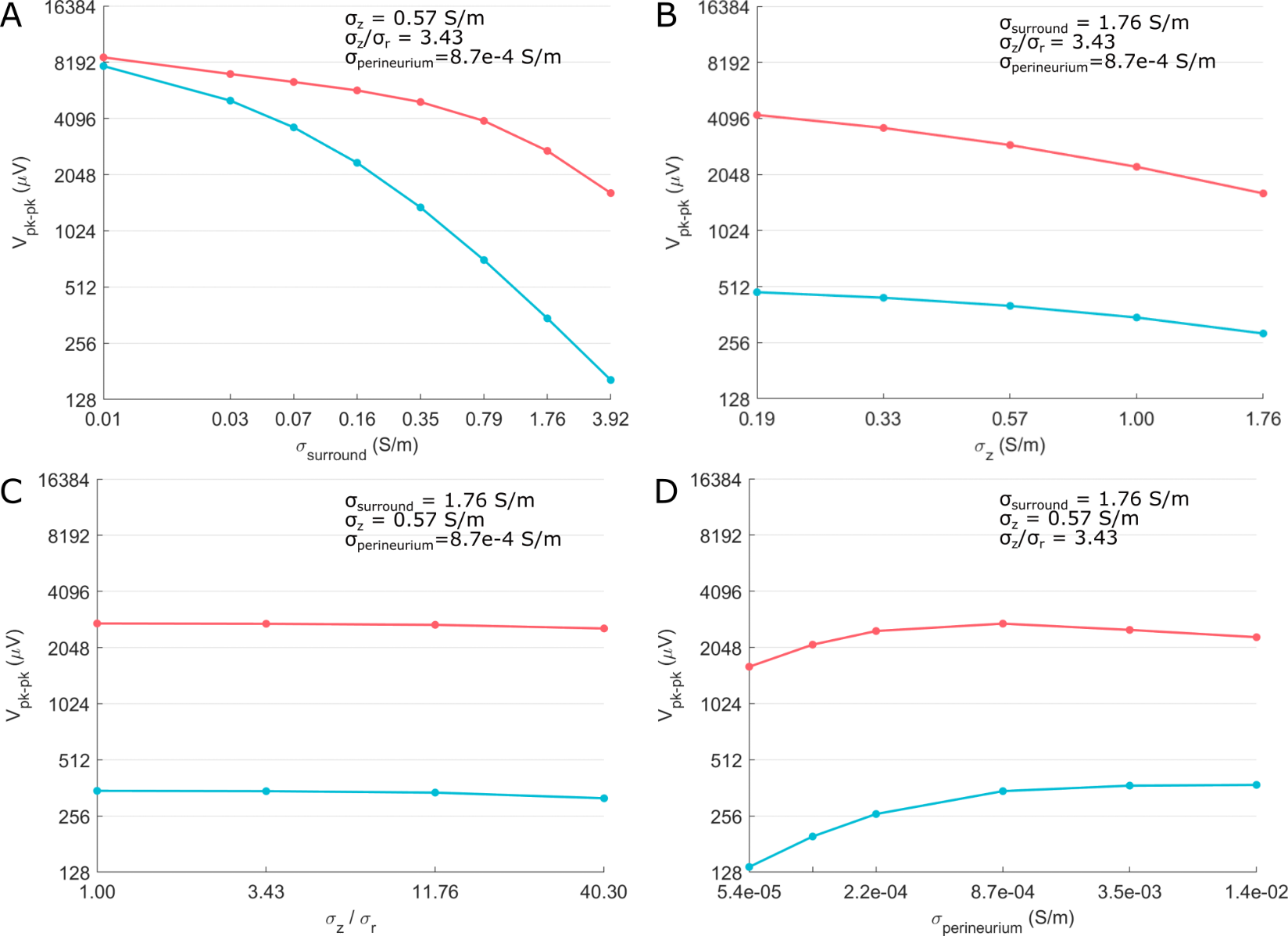
*

*Figure A. Same as Figure 6, but for unmyelinated fibers.*
