# Supplementary material for: Computational models of compound nerve action potentials: Efficient filter-based methods to quantify effects of tissue conductivities, conduction distance, and nerve fiber parameters
Source: PLoS Comput Biol. 2024 Mar 1;20(3):e1011833. doi: 10.1371/journal.pcbi.1011833 (PMC10936855; doi:10.1371/journal.pcbi.1011833)
Supplement: S13 Text — (DOCX) [file pcbi.1011833.s013.docx]

S13 Text: Tissue Conductivities Effects on Myelinated Fiber CNAPs (full, with cuff opening)


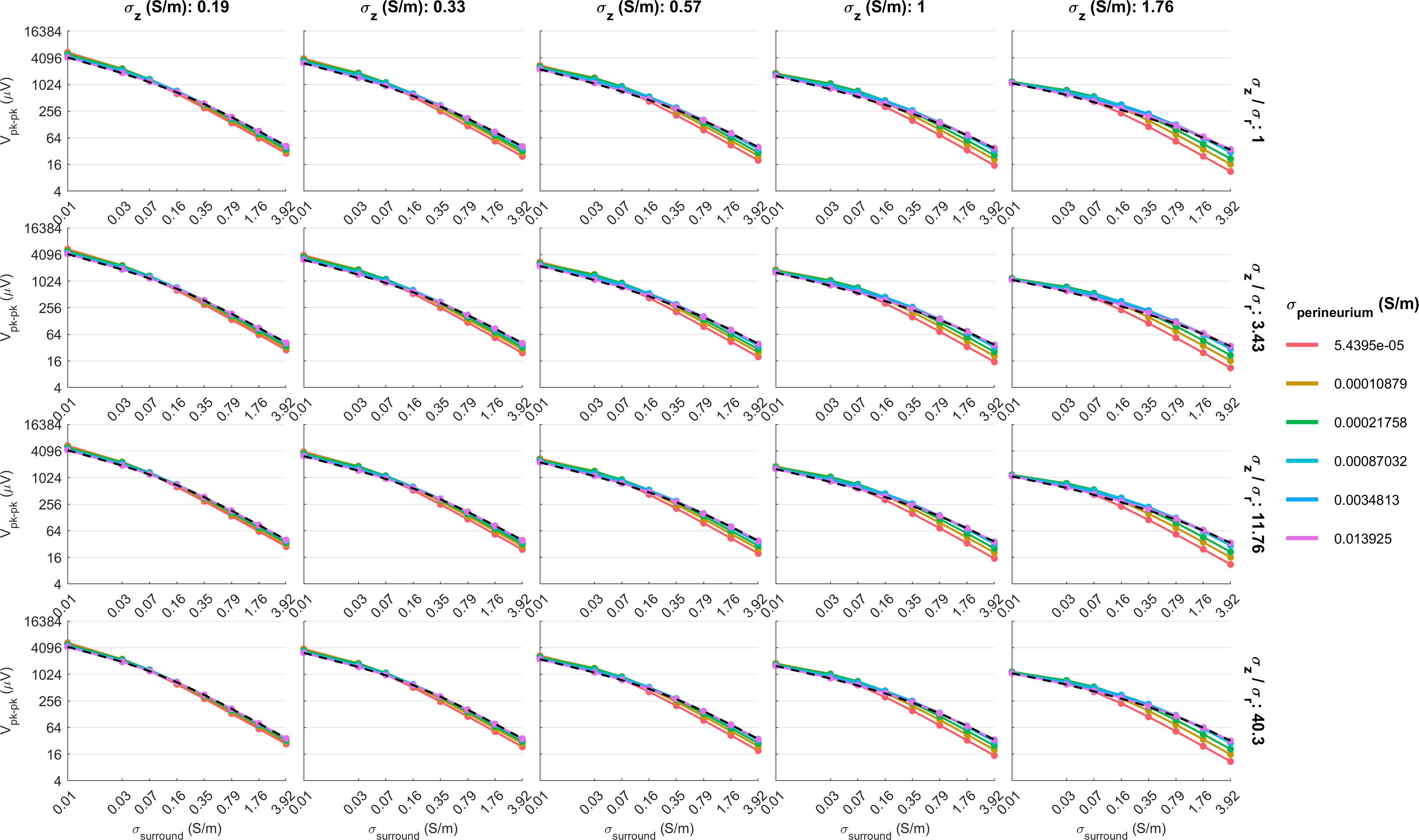


*Figure A. Sensitivity analysis with a sealed cuff (16° opening) of surround conductivity, perineurium conductivity, endoneurium longitudinal conductivity, and endoneurial anisotropy (= endoneurium longitudinal conductivity / endoneurium radial conductivity) on myelinated fiber CNAP peak-to-peak signal amplitude. The analysis includes a simulation with no perineurium (black dashed line).*
