# Supplementary material for: Computational models of compound nerve action potentials: Efficient filter-based methods to quantify effects of tissue conductivities, conduction distance, and nerve fiber parameters
Source: PLoS Comput Biol. 2024 Mar 1;20(3):e1011833. doi: 10.1371/journal.pcbi.1011833 (PMC10936855; doi:10.1371/journal.pcbi.1011833)
Supplement: S15 Text — (DOCX) [file pcbi.1011833.s015.docx]

S15 Text: Tissue Conductivities Effects on Unmyelinated Fiber CNAPs Waveform shape


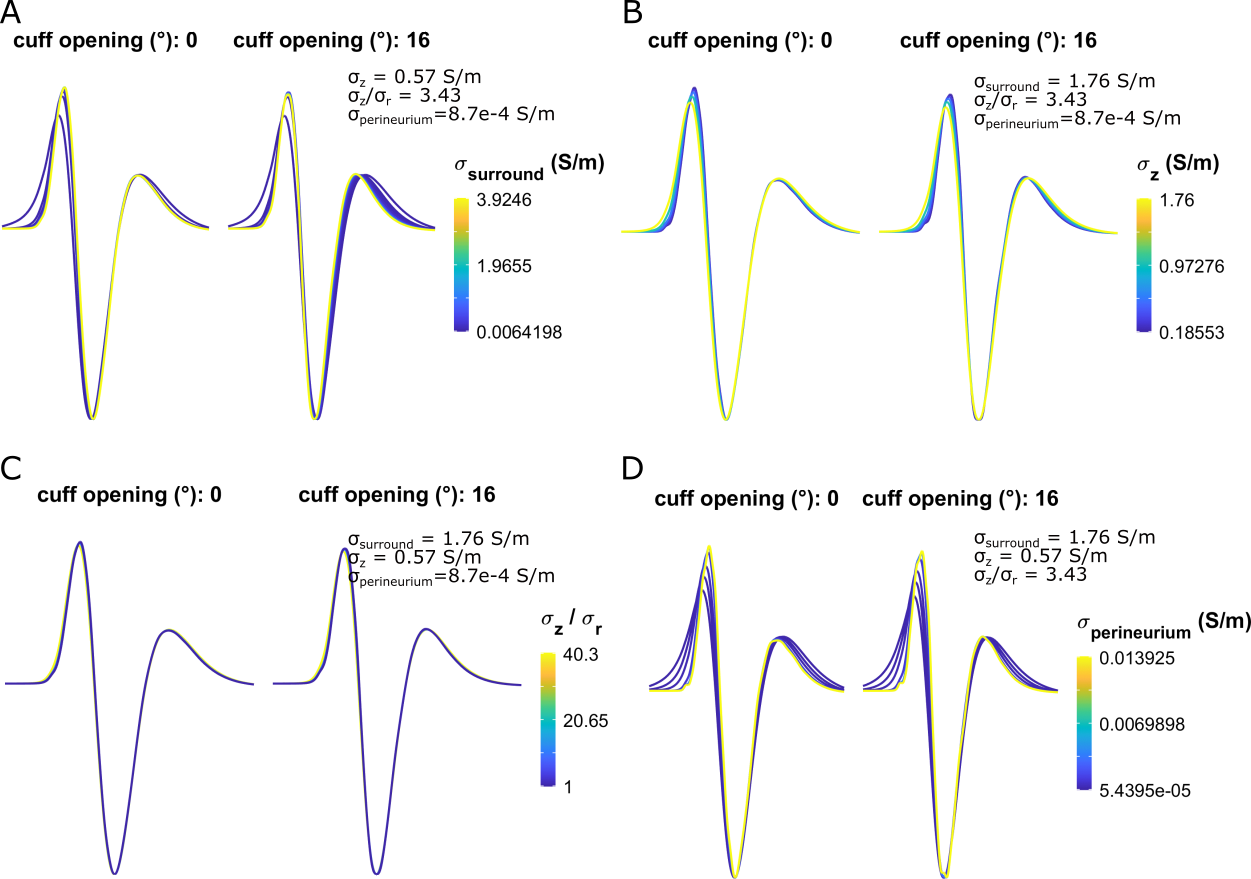


*Figure A. Sensitivity analysis of unmyelinated fiber CNAP waveforms for all tissue conducitivties and cuff openings shown in Figure 6. Each subpanel shows a normalized waveform (to facilitate shape comparison) from t=2 to t=37 ms.*
