# Supplementary material for: Computational models of compound nerve action potentials: Efficient filter-based methods to quantify effects of tissue conductivities, conduction distance, and nerve fiber parameters
Source: PLoS Comput Biol. 2024 Mar 1;20(3):e1011833. doi: 10.1371/journal.pcbi.1011833 (PMC10936855; doi:10.1371/journal.pcbi.1011833)
Supplement: S16 Text — (DOCX) [file pcbi.1011833.s016.docx]

S16 Text: Effect of Random Sampling in Unmyelinated Fibers


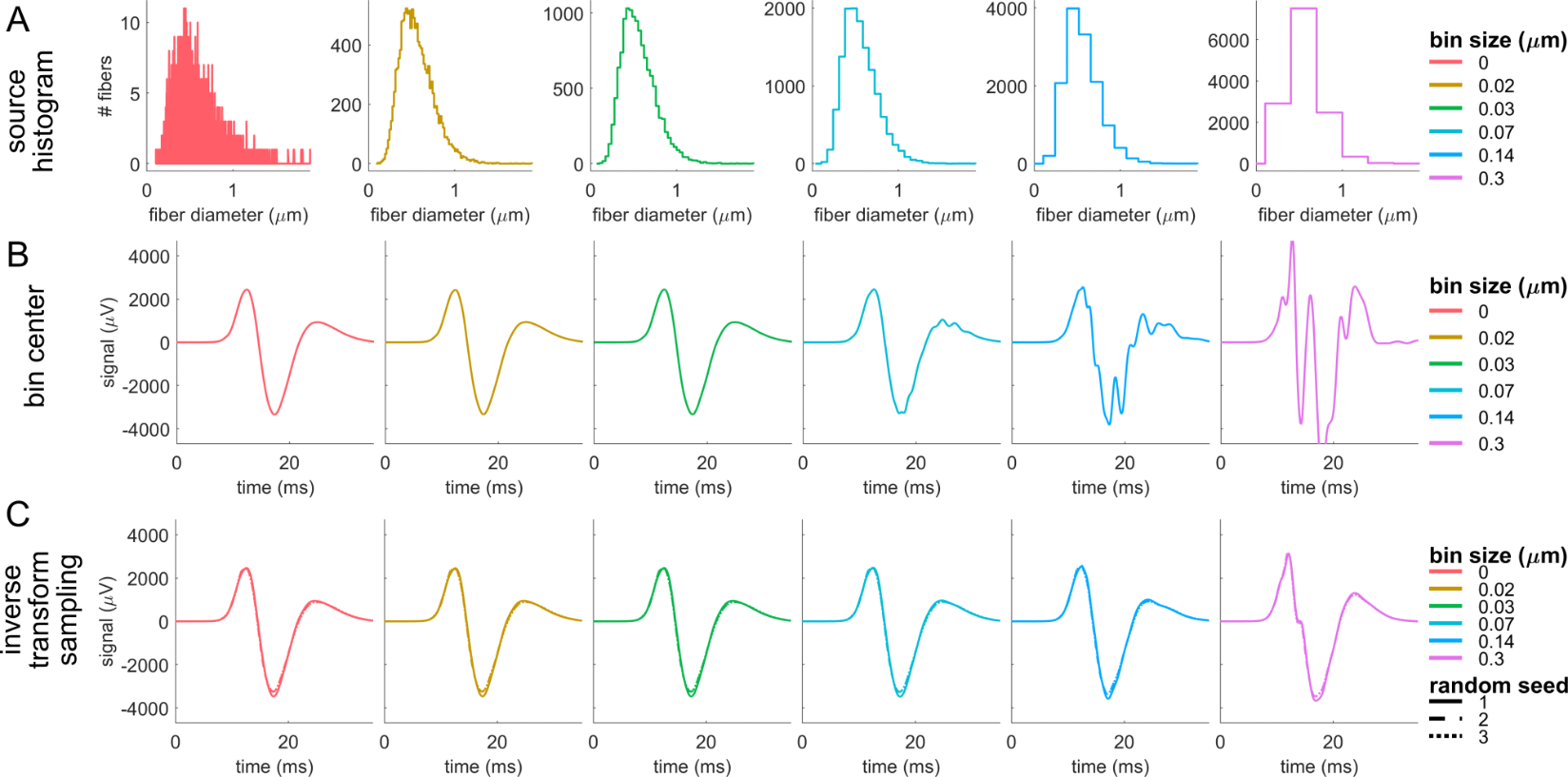


*Figure A. Effect of bin size and sampling method on unmyelinated fiber CNAPs during extraction of fiber diameters from distributions. (A) Histograms of known unmyelinated fiber diameters across different bin sizes. A bin size of 0 µm used the individual fiber diameter measurements (precision 1e-6 µm). (B) Effect on CNAPs of generating fiber diameters based on the center of the bin and the bin height. As bin size increased, using the bin centers produced inaccuracies due to less destructive interference and more constructive interference. (C) Effect on CNAPs of generating fiber diameters based on inverse transform sampling to randomly sample diameters from the estimated cumulative distribution function. For a given non-zero bin size, CNAPs were more accurate than when using bin centers. Conduction distance was 11 mm center-to-center.*
