# Supplementary material for: Computational models of compound nerve action potentials: Efficient filter-based methods to quantify effects of tissue conductivities, conduction distance, and nerve fiber parameters
Source: PLoS Comput Biol. 2024 Mar 1;20(3):e1011833. doi: 10.1371/journal.pcbi.1011833 (PMC10936855; doi:10.1371/journal.pcbi.1011833)
Supplement: S17 Text — (DOCX) [file pcbi.1011833.s017.docx]

S17 Text: CV vs. Fiber Diameter Relationship in Models

| A  CV = 4.01*fiberD – 2.5  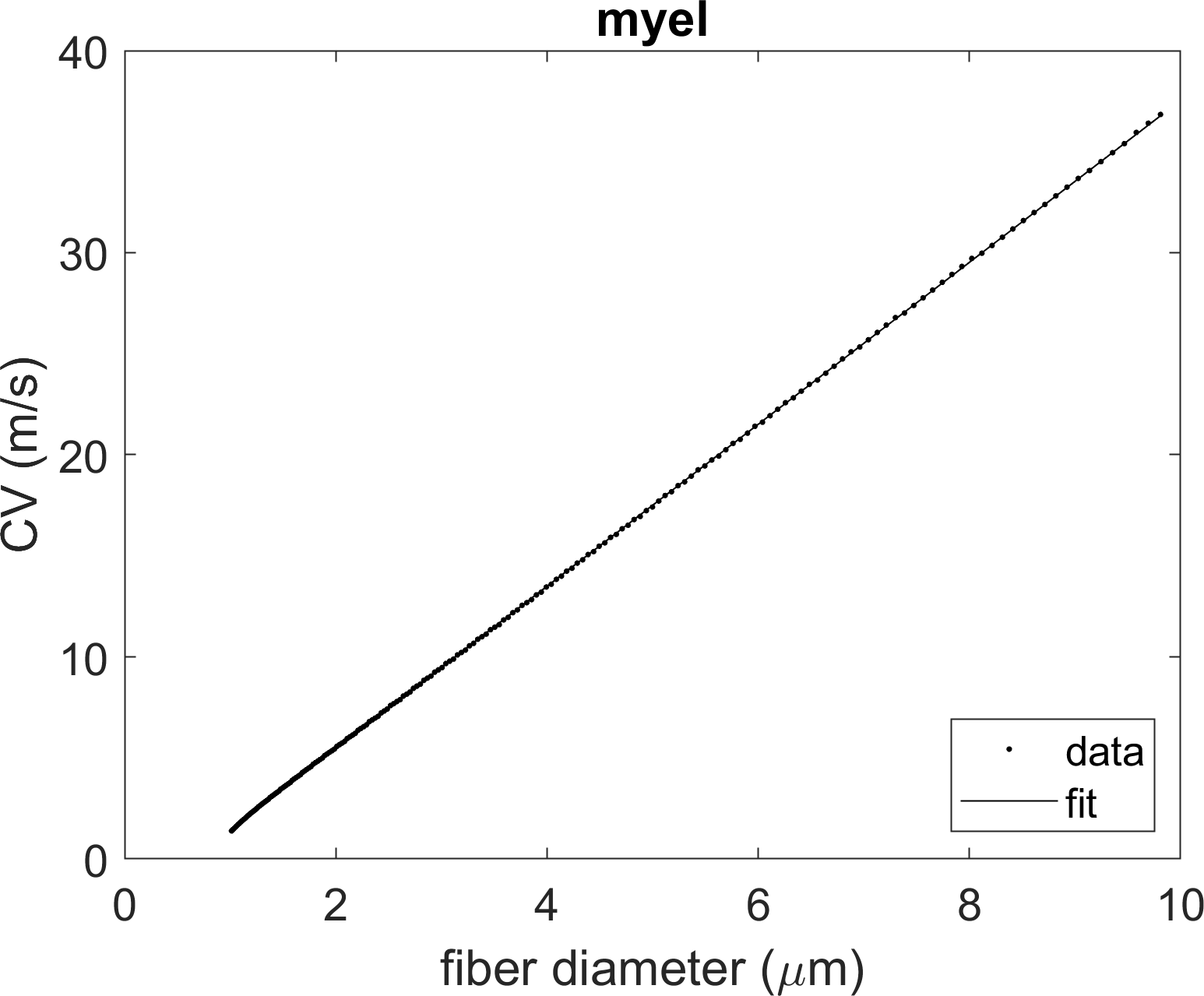 | B  CV = 0.70*sqrt(fiberD) – 1.9e-3  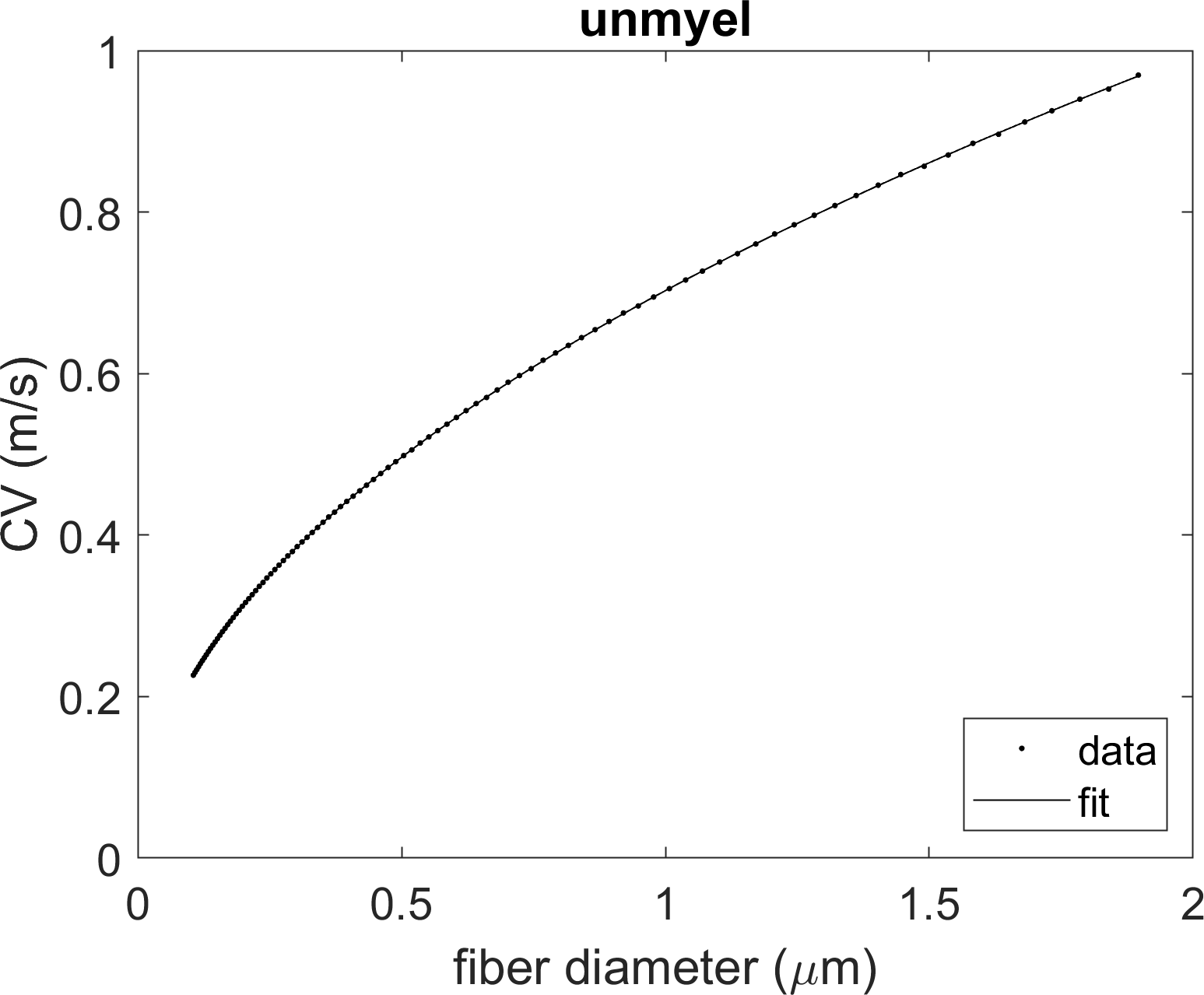 |
| --- | --- |

*Figure A. Fits of CV vs. fiber diameter relationships in modeled myelinated fibers (A) and unmyelinated fibers (B). Fits take fiberD in micrometers and produce CV in meters per second.*
