# Supplementary material for: Computational models of compound nerve action potentials: Efficient filter-based methods to quantify effects of tissue conductivities, conduction distance, and nerve fiber parameters
Source: PLoS Comput Biol. 2024 Mar 1;20(3):e1011833. doi: 10.1371/journal.pcbi.1011833 (PMC10936855; doi:10.1371/journal.pcbi.1011833)
Supplement: S18 Text — (DOCX) [file pcbi.1011833.s018.docx]

S18 Text: CV vs. Fiber Diameter Relationship in Literature

| A  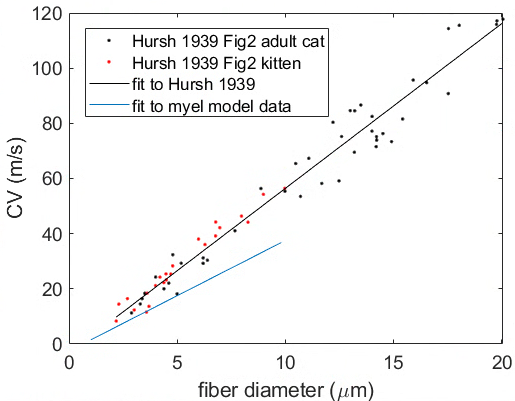 | B  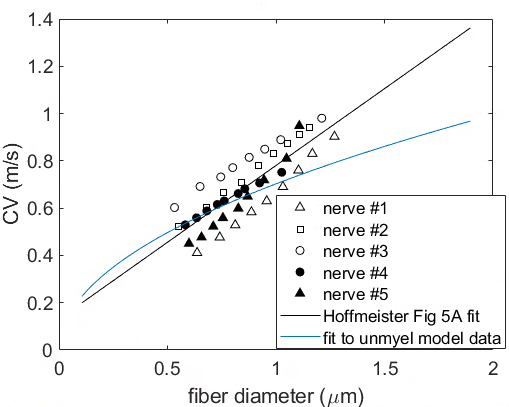 |
| --- | --- |

*Figure A. In vivo CV data vs. model CV data for myelinated (A) and unmyelinated (B) fibers. (B) Nerve numbers represent a distinct nerve from Fig 5A of (80). The shape and fill of the markers correspond to the shape and fill used in Fig 5A of (80).*
