# Supplementary material for: Computational models of compound nerve action potentials: Efficient filter-based methods to quantify effects of tissue conductivities, conduction distance, and nerve fiber parameters
Source: PLoS Comput Biol. 2024 Mar 1;20(3):e1011833. doi: 10.1371/journal.pcbi.1011833 (PMC10936855; doi:10.1371/journal.pcbi.1011833)
Supplement: S19 Text — (DOCX) [file pcbi.1011833.s019.docx]

S19 Text: Effect of Longitudinal Jitter

*Figure A. Effect of longitudinal jitter on SFAPs from myelinated fibers. ‘Baseline’ fibers had their middle compartments centered at the longitudinal middle of the nerve while ‘z jittered’ fibers were shuffled by random distances in the z direction such that the distances ranged from 0% to 100% of each fiber’s internodal distance.*
