# Supplementary material for: Computational models of compound nerve action potentials: Efficient filter-based methods to quantify effects of tissue conductivities, conduction distance, and nerve fiber parameters
Source: PLoS Comput Biol. 2024 Mar 1;20(3):e1011833. doi: 10.1371/journal.pcbi.1011833 (PMC10936855; doi:10.1371/journal.pcbi.1011833)
Supplement: S20 Text — (DOCX) [file pcbi.1011833.s020.docx]

S20 Text: Temporal Dispersion In Vivo vs. Model


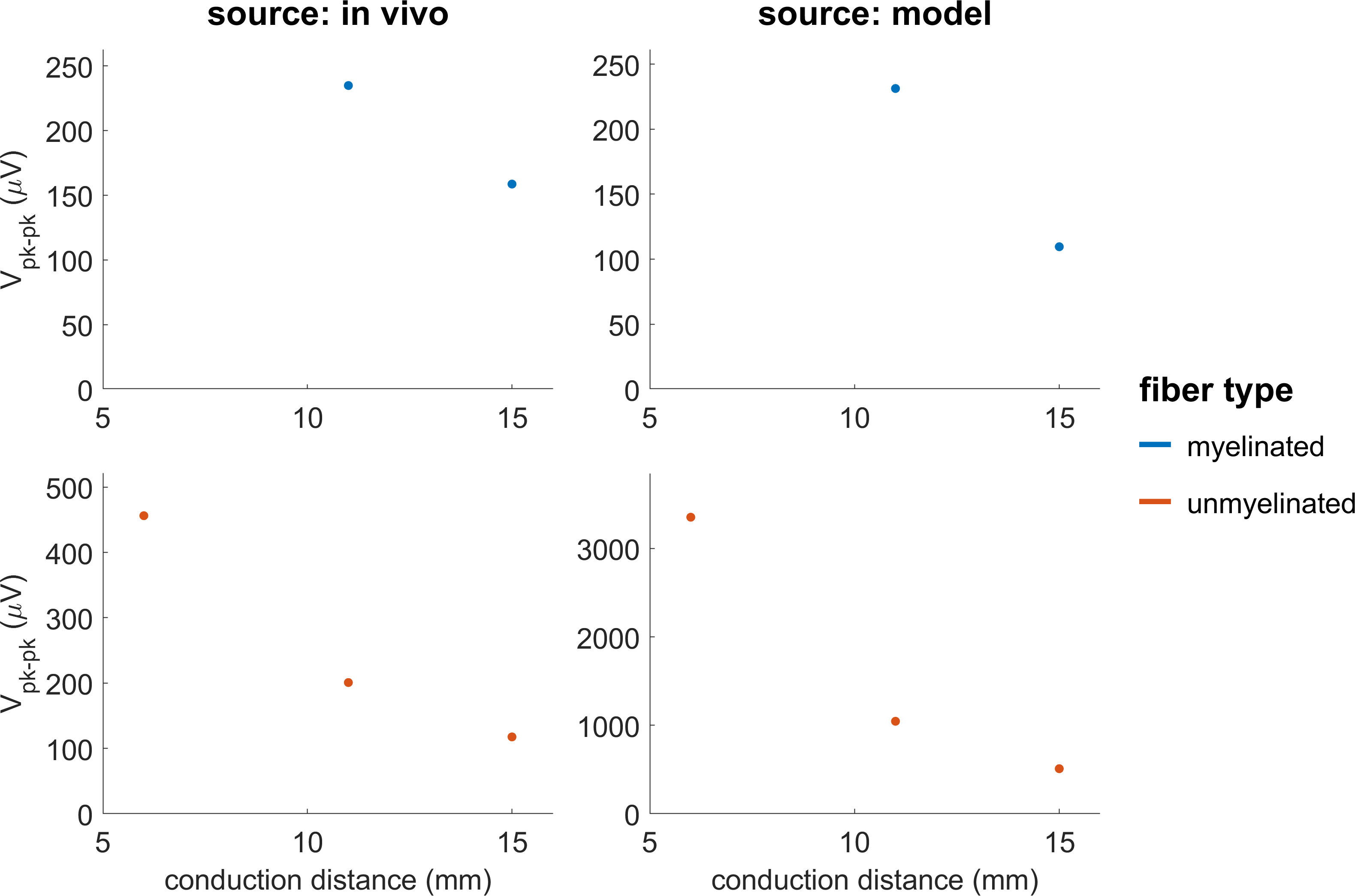


*Figure A. CNAP peak-to-peak amplitude vs. conduction distance for in vivo and model data from Figure 13A,C. The relative decrease in amplitude due to increasing conduction distance (i.e., temporal dispersion) was comparable for model vs. in vivo data and for myelinated vs. unmyelinated data.*
