# Supplementary material for: Computational models of compound nerve action potentials: Efficient filter-based methods to quantify effects of tissue conductivities, conduction distance, and nerve fiber parameters
Source: PLoS Comput Biol. 2024 Mar 1;20(3):e1011833. doi: 10.1371/journal.pcbi.1011833 (PMC10936855; doi:10.1371/journal.pcbi.1011833)
Supplement: S21 Text — (DOCX) [file pcbi.1011833.s021.docx]

S21 Text: Frequency Content of Myelinated and Unmyelinated Fibers

*
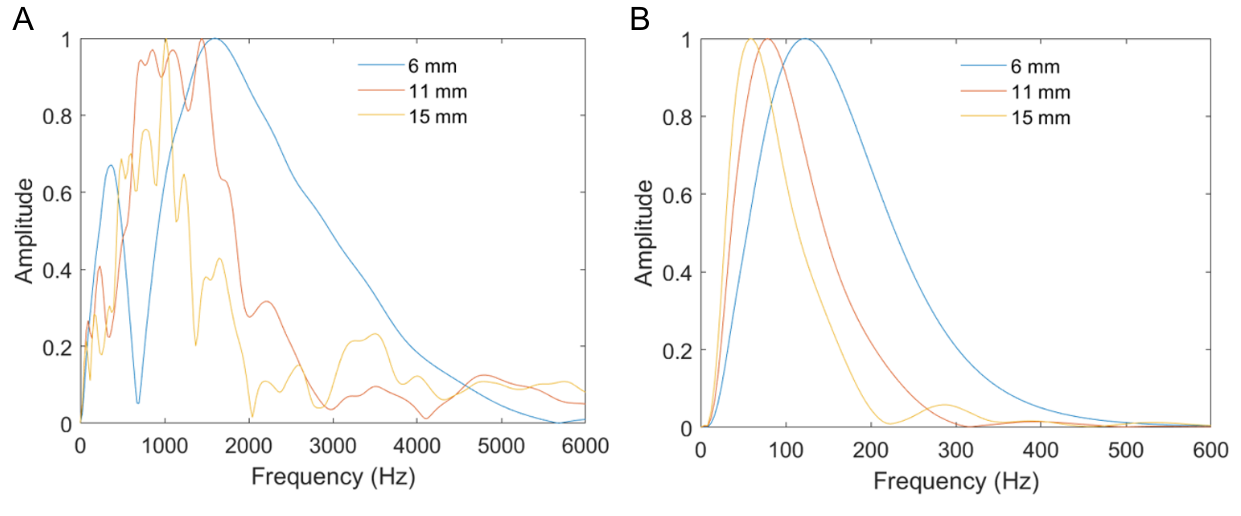
*

*Figure A. Frequency content of modeled CNAPs shown in Figure 13A, normalized to a peak of 1. The modeled signals underlying the spectral analyses were recorded at three conduction distances: 6, 11, 15 mm. Low frequencies comprised a larger proportion of the frequency content at larger conduction distances due to temporal dispersion. The modeled signals underlying the spectral analyses were recorded at three conduction distances: 6, 11, 15 mm. The peak frequency decreased as conduction distance increased due to temporal dispersion.*
