# Supplementary material for: Computational models of compound nerve action potentials: Efficient filter-based methods to quantify effects of tissue conductivities, conduction distance, and nerve fiber parameters
Source: PLoS Comput Biol. 2024 Mar 1;20(3):e1011833. doi: 10.1371/journal.pcbi.1011833 (PMC10936855; doi:10.1371/journal.pcbi.1011833)
Supplement: S22 Text — (DOCX) [file pcbi.1011833.s022.docx]

S22 Text: Analog Filtering


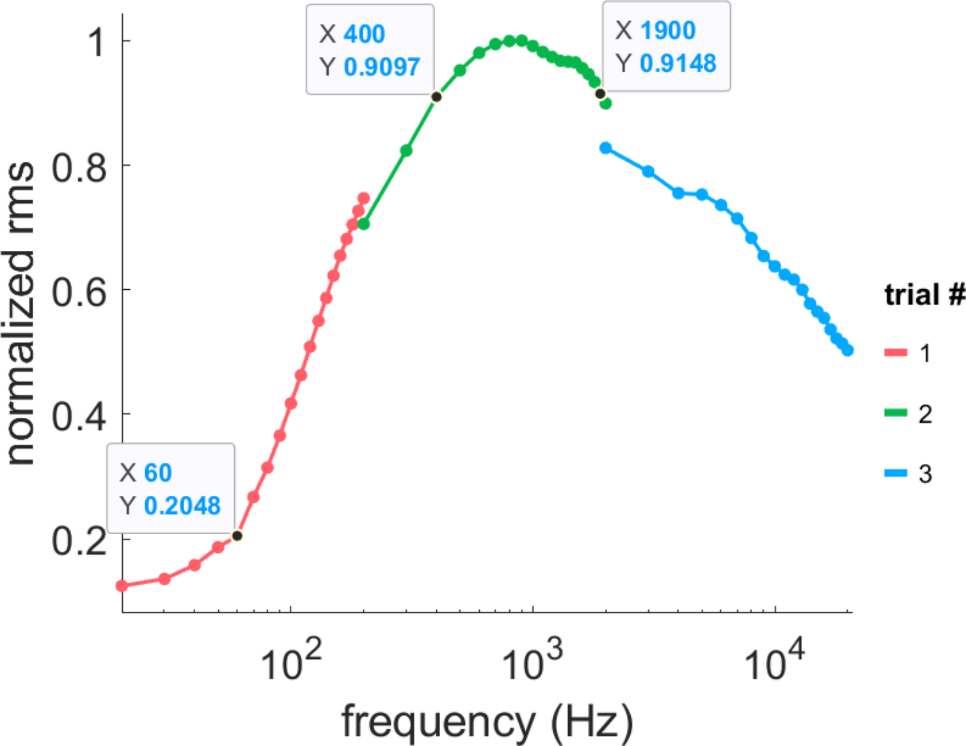


*Figure A. Frequency response of recording setup illustrated in Figure 3. Signals were recorded from the middle contact of the tripolar electrode implanted on a rat cervical vagus nerve (with a nearby muscle as reference). Instead of applying stimulation pulses to evoke CNAPs, we manually applied a 0.1 mA sinusoidal signal between the rostral and caudal contacts of the tripolar electrode across 20 to 200 Hz in 10 Hz increments (trial #1), 200 to 2,000 Hz in 100 Hz increments (trial #2), and 2,000 to 20,000 Hz in 1,000 Hz increments (trial #3). We applied each frequency for 5 seconds and recorded the signal in LabChart. The plots show the rms of each 5 second window (excluding 0.5 seconds on each window edge to avoid edge effects), normalized to the highest rms value recorded. Highlighted data points show powerline frequency (60 Hz) and the frequencies at which the rms was 90% or higher of the highest rms (400 Hz and 1,900 Hz). Discontinuities were apparent between trials at 200 Hz and 2,000 Hz.*
