# Supplementary material for: Computational models of compound nerve action potentials: Efficient filter-based methods to quantify effects of tissue conductivities, conduction distance, and nerve fiber parameters
Source: PLoS Comput Biol. 2024 Mar 1;20(3):e1011833. doi: 10.1371/journal.pcbi.1011833 (PMC10936855; doi:10.1371/journal.pcbi.1011833)
Supplement: S23 Text — (DOCX) [file pcbi.1011833.s023.docx]

S23 Text: No Perineurium


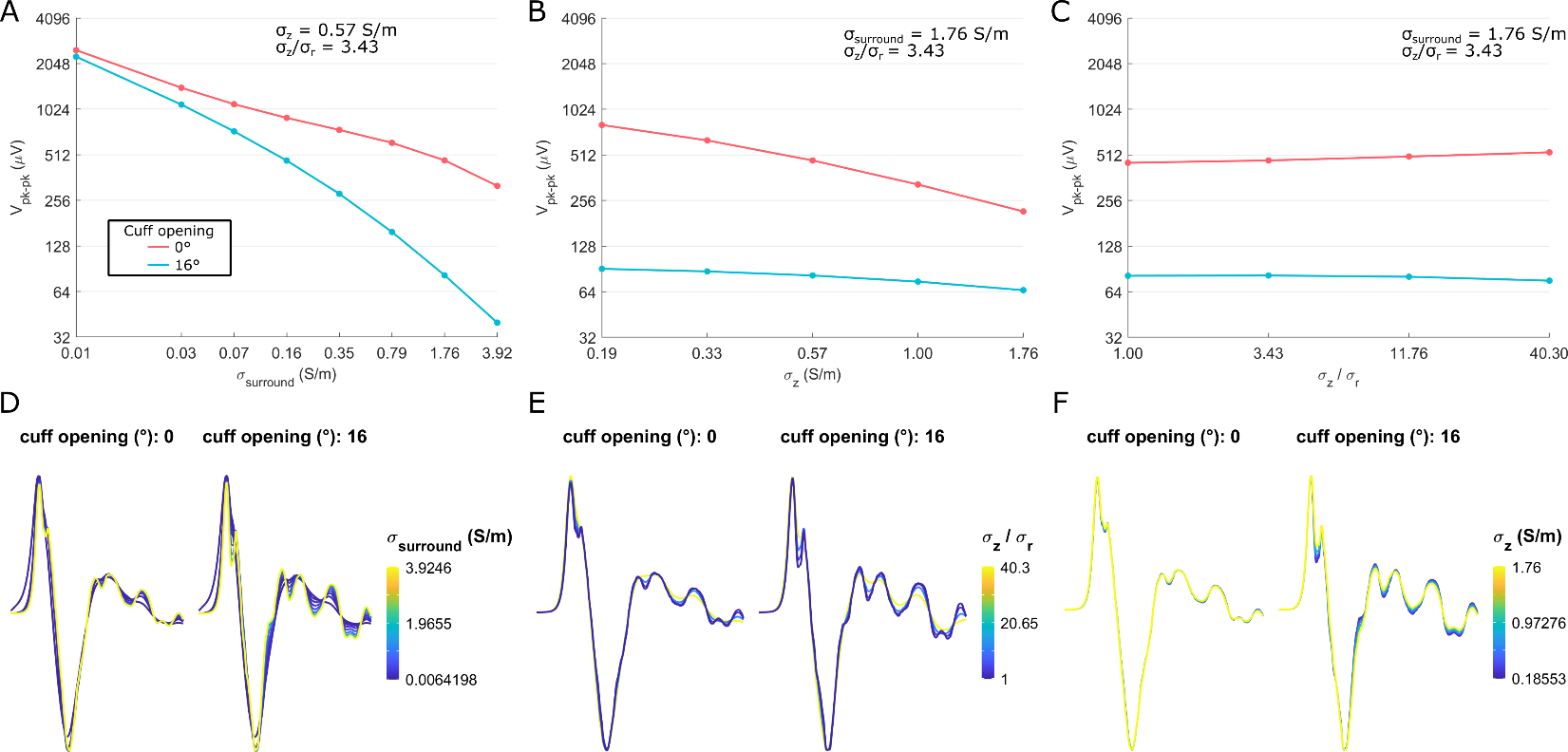


*Figure A. Effects of volume conductor parameters on CNAP amplitude and shape in the absence of perineurium. All model parameters are the same for Figure 6 and Figure 7, except that the perineurium was removed. Removing the perineurium only slightly changed the effects of other tissue parameters on the CNAPs: endoneurial anisotropy had a slightly stronger effect on amplitude, surround conductivity had a slightly stronger effect on shape, and endoneurial anisotropy and endoneurial longitudinal conductivity had a weaker effect on waveform shape.*
